# Global burden, inequality, and frontier gaps of autism spectrum disorder disability in adolescents and young adults, 1990–2021: a systematic analysis of the GBD 2021 study

**DOI:** 10.3389/fpubh.2025.1681565

**Published:** 2025-10-16

**Authors:** Derong Lin, Zhuangtang Shi, Zhen Hao, Xiaohua Xie, Jingya Fang, Mei Li, Weiqing Zhang, Shuxiong Luo, Aiguo Xue

**Affiliations:** ^1^Dongguan Hospital of Guangzhou University of Chinese Medicine, Dongguan, China; ^2^Clinical Medical College of Acupuncture, Moxibustion and Rehabilitation, Guangzhou University of Chinese Medicine, Guangzhou, China; ^3^Zhuhai Chronic Disease Prevention and Treatment Centre, Zhuhai, China; ^4^The People’s Hospital of Longhua, Shenzhen, China

**Keywords:** autism spectrum disorder, adolescents and young adults, disability-adjusted life years, cross-country inequality, frontier analysis

## Abstract

**Background:**

Autism spectrum disorder (ASD) ranks among the leading causes of years lived with disability in adolescence and young adulthood (AYA), yet global assessments still focus on childhood and seldom examine how national development modifies burden.

**Methods:**

We analysed Global Burden of Disease 2021 data for 204 countries and territories from 1990 to 2021. Among AYA aged 15–39 years, we extracted the age-standardised prevalence rate (ASPR) and disability-adjusted life-year rate (ASDR), stratified by sex, five-year age groups and Sociodemographic Index (SDI) quintile. Temporal trends were evaluated using the Estimated Annual Percentage Change (EAPC). Cross-country absolute and relative inequalities were quantified with the Slope Index of Inequality (SII) and Concentration Index (CIX). A half-normal stochastic frontier model defined the minimum attainable ASDR for each SDI level; country-year gaps were calculated as observed minus frontier values.

**Results:**

From 1990 to 2021, prevalent ASD cases increased from 17.52 to 24.13 million and DALYs from 3.30 to 4.55 million. Despite higher counts, global age-standardised rates changed little: in 2021 the ASPR was 811.67 per 100000 (95% UI 683.34–952.87) and ASDR 153.00 (95% UI 103.77–215.64); EAPCs were near zero. Males contributed about two-thirds of the burden (rate ratio ≈2.1). Disability rose most at ages 30–39 (+56%). A persistent SDI gradient was observed: high-SDI settings recorded ASPR 1090.72 and ASDR 205.00 versus 845.15 and 158.57 in low-SDI settings. In 2021, SII was 22.53 (95% UI 12.53–32.53) and CIX 0.04 (95% UI 0.02–0.05). Several high-income economies exceeded the frontier, while Bangladesh, Somalia and Niger lay on or below it—likely reflecting surveillance gaps rather than low burden.

**Conclusion:**

Absolute ASD disability in AYA has risen mainly from population growth and case detection, not higher per capita risk. A sustained male predominance, a renewed peak at ages 30–39, and minimal progress on inequality show that economic gains alone have not reduced burden. Expanding adult screening, vocational support and community-based interventions, alongside stronger surveillance and parent training in low-SDI settings, is required to narrow global gaps.

## 1 Introduction

Autism spectrum disorder (ASD) is a neurodevelopmental condition that emerges in early childhood and is characterised by persistent deficits in social communication alongside restricted, repetitive interests and behaviours. The disorder frequently co-occurs with other psychiatric conditions, persists throughout the life course, and imposes substantial socioeconomic burdens on education, employment and family functioning (1).

Global estimates generated with hierarchical Bayesian models show that from 1990 to 2019 the absolute number of people living with ASD increased by more than 50%, whereas the global age-standardised prevalence rate (ASPR) remained essentially unchanged—a pattern of rising case counts despite stable rates (2). This “latent expansion” suggests that improved diagnostic reach has not been matched by commensurate service provision and that rate-based surveillance alone may underestimate demand.

According to GBD 2021, an estimated 61.8 million people (approximately 1 in 127 globally) were living with ASD in 2021. Adolescents and young adults (AYA; 15–39 years) account for about 40% of this total, and ASD ranks among the leading causes of disability in this age group. While high-SDI regions currently have the highest ASPR and the age-standardised disability-adjusted life-year (DALY) rate (ASDR), middle-SDI regions exhibit the fastest growth in cases. In contrast, low-SDI settings likely underestimate the ASD burden because of underdiagnosis and limited resources (3).

Electronic health record data from twelve large U.S. health systems further reveal a 450% surge in diagnosed ASD among adults aged 25–34 years between 2011 and 2019—the steepest increase observed across all age strata (4). Longitudinal cohort studies likewise point to a second peak of functional loss around age 30, forming an inverted U-shaped trajectory across the life course (5). Together, these findings underscore a dual vulnerability during the AYA period, in which major social role transitions coincide with gaps in service provision. However, the spatiotemporal burden in this subgroup remains poorly quantified. Previous global assessments have focused primarily on children aged 0–14 years, leaving the epidemiological profile and service needs of AYA populations systematically underestimated (6).

To address these evidence deficits, we used fully updated GBD 2021 data and methods to quantify ASD prevalence and DALYs among 15–39-year-olds in 204 countries and territories from 1990 to 2021, assessed trends using the Estimated Annual Percentage Change (EAPC), and evaluated cross-country absolute inequality (Slope Index of Inequality, SII) and relative inequality (Concentration Index, CIX) alongside stochastic frontier analysis (SFA) to compute gaps between observed and frontier-attainable ASDR. By identifying high-risk populations and unexploited efficiency gains, our study aims to inform evidence-based screening, intervention and social protection strategies grounded in lifelong support and neurodiversity inclusion, thereby advancing the Lancet Commission on Autism’s call for disability-inclusive societies by 2030 (1).

## 2 Methods

### 2.1 Data sources

We performed a cross-sectional ecological analysis using data from the Global Burden of Disease Study 2021 (GBD 2021) to assess the burden of ASD among individuals aged 15–39 years across 204 countries and territories between 1990 and 2021 (7). The study followed GATHER and STROBE guidelines and adhered to the GBD 2021 methodological framework for mental disorders. We obtained primary estimates from the Global Health Data Exchange (GHDx) using the GBD Results Tool, downloading 500 posterior draws for each location–year–sex–age–metric to ensure traceability (8).

### 2.2 Case definition and population stratification

In GBD 2021, ASD is identified by cause code 645, corresponding to ICD-10 F84.0–F84.9; cases originally coded under ICD-9 were mapped to ICD-10 using the official crosswalk. Our analysis focused on adolescents and young adults (AYA; 15–39 years), grouped into five-year age groups (15–19, 20–24, …, 35–39). All estimates were stratified by sex and by Sociodemographic Index (SDI) quintile (3).

### 2.3 Estimation framework and metric calculation

GBD 2021 used the Bayesian meta-regression tool DisMod-MR 2.1 to synthesise evidence from multiple sources (peer-reviewed studies, outpatient and community surveys), producing location-, age- and sex-specific estimates of ASD prevalence, incidence and excess mortality. The excess-mortality component ensures internal epidemiological consistency (9). Because ASD is not coded as an underlying cause of death, DALYs equate to years lived with disability (YLDs) alone (10):


YLD=Prevalence×Disability Weight(DW)

All rates were age-standardised to the GBD world standard population to enable temporal and spatial comparability.

### 2.4 Trend analysis and uncertainty quantification

We summarised temporal trends using the estimated annual percentage change (EAPC):


EAPC=100×(exp(β)−1)

where β is the slope of the log-transformed age-standardised rate (ASR) regressed against calendar year; 95% CI were derived analogously (11).

### 2.5 Stratified comparisons and correlation tests

For each SDI quintile we reported total ASD DALYs and the EAPC of the age-standardised rate over 1990–2021. Sex differences were assessed using male-to-female rate ratios and by examining the non-overlap of 95% UIs. To explore macro-level determinants, we used Spearman’s rank correlation to examine associations between age-standardised DALY rates and country-level indicators (SDI and national per capita health expenditure) (12).

### 2.6 Inequality and frontier analyses

We ranked countries annually by SDI (0–1) and quantified cross-country inequality using two summary measures: the Slope Index of Inequality (SII)—the population-weighted regression gap between the lowest- and highest-SDI positions—and the Concentration Index (CIX)—twice the covariance between the metric (e.g., ASDR for ASD) and the fractional SDI rank, divided by the mean. Point estimates were computed on the country-year dataset. Uncertainty for SII/CIX was derived using non-parametric bootstrap with 1,000 population-weighted resamples of countries (with replacement), and 95% UIs were taken as the 2.5th–97.5th percentiles across bootstrap replicates (13, 14).

### 2.7 Visualisation and statistical software

All data processing and visualisation were performed in R (version 4.4.2). Two-sided *p* values < 0.05 were considered statistically significant.

### 2.8 Ethical statement and data sharing

This study used only publicly available, de-identified data and thus did not require any additional ethical approval. Analysis scripts and derived datasets can be obtained from the corresponding author upon reasonable request to facilitate replication and secondary use.

## 3 Results

### 3.1 Global burden among 15–39-year-olds, 1990–2021

**Figure 1:**
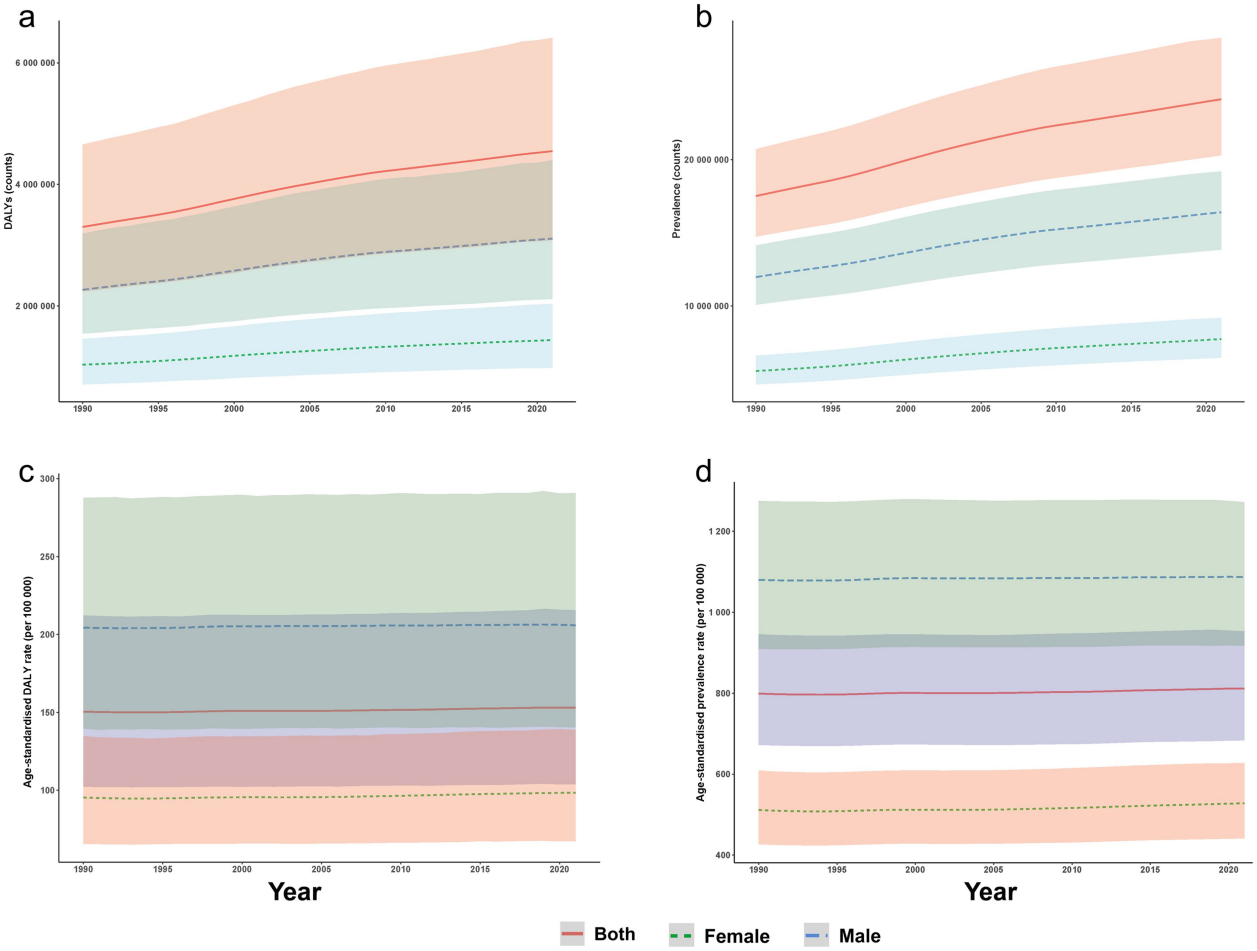
Global burden of ASD among AYA (15–39 years) from 1990 to 2021, stratified by sex. **(a)** Total DALYs cases; **(b)** total prevalent cases; **(c)** age-standardised prevalence rate; and **(d)** age-standardised DALY rate, each displayed separately for males and females. Shaded bands denote 95% uncertainty intervals. ASD, autism spectrum disorders; DALY, disability-adjusted life year; AYA, adolescents and young adults.

From 1990 to 2021, the number of AYA (15–39 years) living with ASD increased and then plateaued, rising from 17.52 million (95% UI 14.74–20.72 million) in 1990 to 24.13 million (95% UI 20.30–28.33 million) in 2021. Over the same period, total DALYs climbed from 3.30 million (95% UI 2.24–4.65 million) to 4.55 million (95% UI 3.08–6.41 million) (Figures 1a,b). Despite this rise in absolute burden, the global ASPR increased only marginally—from 799.04 to 811.67 per 1,00,000—while the ASDR remained essentially stable at approximately 150 per 1,00,000 (150.49 in 1990 and 153.00 in 2021) (Figures 1c,d).

### 3.2 Sex-specific profile in 2021

**Figure 2:**
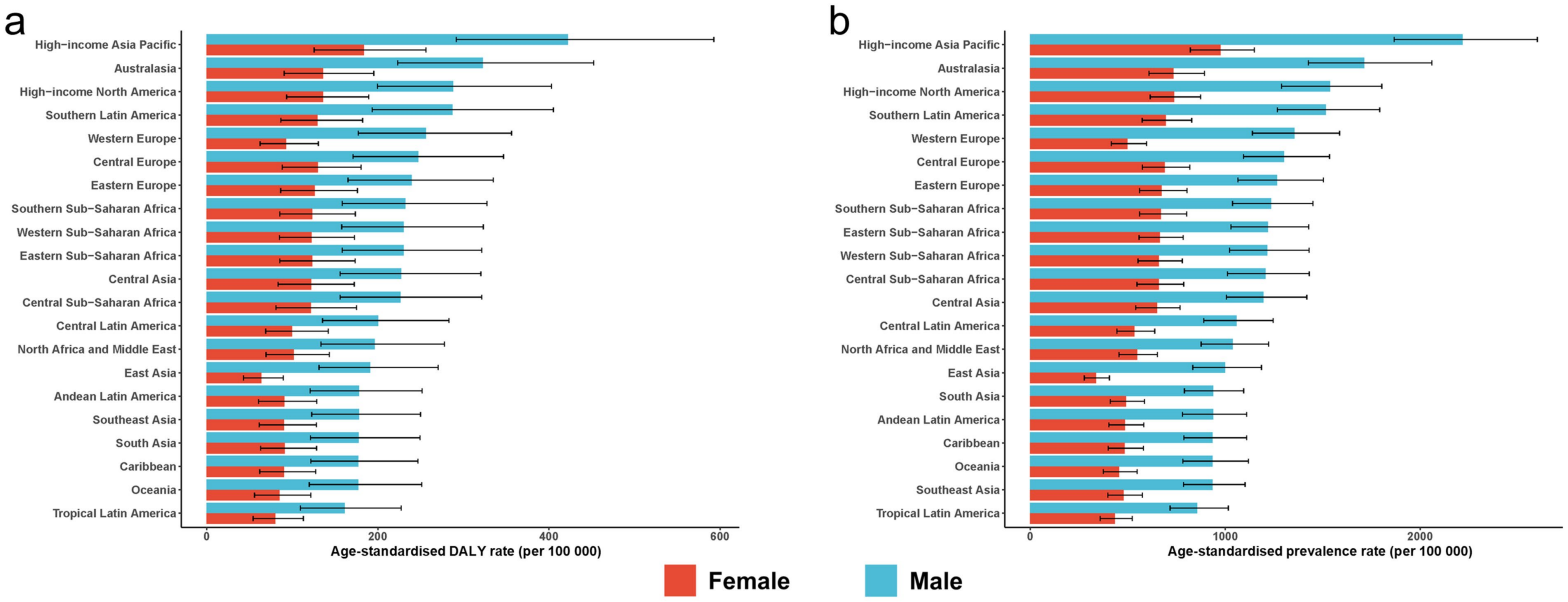
Age-standardised burden of ASD among AYA in 2021. **(a)** Age-standardised DALY rate; **(b)** age-standardised prevalence rate. Error bars denote 95% uncertainty intervals. ASD, autism spectrum disorders; DALYs, disability-adjusted life years; AYA, adolescents and young adults.

In 2021, males accounted for 68% of global ASD cases (16.40 million; 95% UI 13.84–19.20 million) and 69% of ASD DALYs (3.11 million; 95% UI 2.12–4.40 million), representing 2.1 and 2.2 times the female totals, respectively. The male ASPR was 1086.79 per 1,00,000, compared with 528.10 per 1,00,000 in females, and the corresponding ASDRs were 205.98 and 98.40 per 1,00,000, yielding a male-to-female rate ratio of approximately 2.1 for both metrics. Between 1990 and 2021, sex-specific ASPR and ASDR changed only slightly (near-zero EAPCs), indicating a nearly constant male-to-female disparity over time (Supplementary Table S9; Figures 1, 2; Supplementary Table S1).

### 3.3 SDI gradients, 1990–2021

In 2021, the ASD burden showed a clear stepwise gradient across SDI quintiles. High-SDI countries recorded the highest ASPR and ASDR, at 1090.72 per 1,00,000 (95% UI 916.84–1284.41) and 205.00 per 1,00,000 (95% UI 140.90–286.86), respectively. These were followed by the high-middle SDI quintile (ASPR 828.72; ASDR 157.11), middle SDI (735.45; 139.04), and low-middle SDI (748.84; 140.88). The low-SDI group showed a modest uptick, reaching 845.15 per 1,00,000 (95% UI 708.88–993.42) for prevalence and 158.57 per 1,00,000 (95% UI 109.42–222.58) for DALYs. Despite this slight irregularity at the lowest quintile, the overall ordered gradient remained largely intact.

Across 1990–2021, ASPRs were essentially flat. The estimated EAPC in the high-SDI quintile was −0.02% (95% UI − 0.75 to 0.72%), and it ranged from 0.06 to 0.20% per year in the other four quintiles. ASDR declined slightly only in high-SDI regions (EAPC −0.01% per year; 95% UI − 0.02 to −0.01%), while increasing marginally in the other quintiles (0.09–0.19% per year) (Supplementary Table S9).

### 3.4 Regional heterogeneity

Among the 21 GBD first-level regions, High-Income Asia Pacific consistently bore the heaviest burden. In 2021, its ASPR was 1616.19 per 1,00,000 (95% UI 1360.84–1896.81) and its ASDR 306.53 per 1,00,000 (95% UI 210.96–430.00). From 1990 to 2021, this region’s ASDR increased by an average of 0.24% per year (95% CI 0.22–0.26) and its ASPR by 0.12% per year (95% CI 0.09–0.15). In contrast, Tropical Latin America had the lowest rates in 2021 (ASPR 644.25; ASDR 120.56 per 1,00,000) and showed virtually no change over time (EAPC 0.01% for ASPR; 0.04% for ASDR, 95% CI 0.03–0.04). Regional rankings remained stable over the three decades: high-income regions stayed at the top, while sub-Saharan Africa and South Asia persisted at lower to middle positions. In every region, male rates were approximately double those of females (Supplementary Table S9).

### 3.5 National-level burden and change

At the national level in 2021, ASD burden showed a dual pattern of “high rate–low population” versus “low rate–high population”: the largest case counts were concentrated in the most populous countries, whereas the highest age-standardised rates occurred in smaller high-income nations. India had the largest number of cases, with 4.53 million (95% UI 3.81–5.31 million) in 2021, followed by China (3.12 million; 95% UI 2.60–3.72 million) and the United States (1.27 million; 95% UI 1.07–1.49 million). Nigeria and Indonesia were also among the top five countries, ranking fourth and fifth with 0.83 million and 0.77 million cases, respectively. India also led in total DALYs (0.852 million; 95% UI 0.581–1.192 million), with China and the United States ranking second and third, respectively.

**Figure 3:**
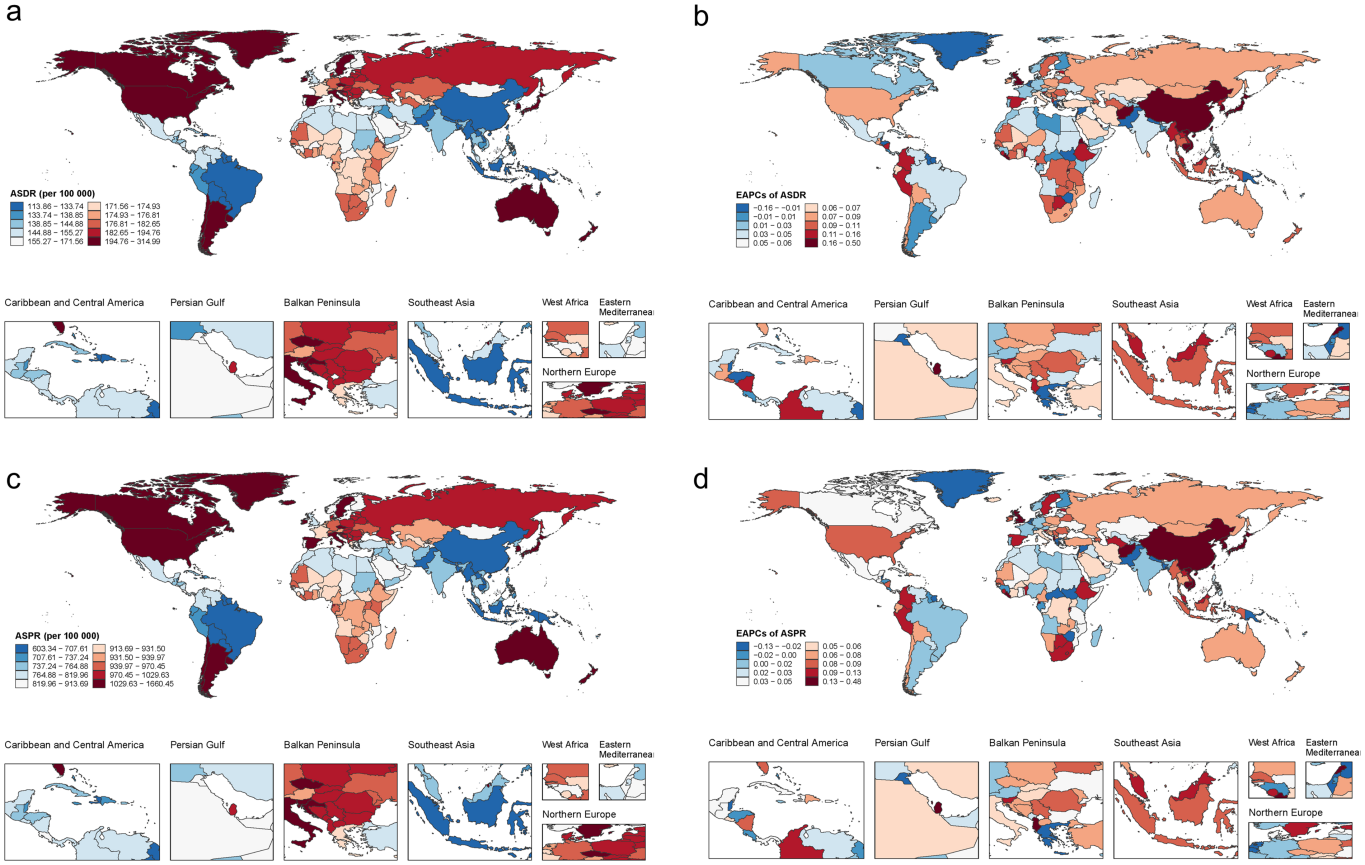
Country-level trends in autism spectrum disorders among AYA, 1990–2021. **(a)** Age-standardised disability-adjusted life-year rate in 2021; **(b)** estimated annual percentage change (EAPC) in ASDR, 1990–2021; **(c)** age-standardised prevalence in 2021; **(d)** EAPC in ASPR, 1990–2021. ASD, autism spectrum disorders; ASPR, age-standardised prevalence rate; ASDR, age-standardised DALY rate; DALYs, disability-adjusted life-years; EAPC, estimated annual percentage change; AYA, adolescents and young adults.

By contrast, Japan recorded the highest age-standardised rates in 2021, with an ASPR of 1644.01 per 1,00,000 (95% UI 1385.28–1930.31) and an ASDR of 311.87 per 1,00,000 (95% UI 215.62–436.99). The Republic of Korea and Singapore had the second- and third-highest rates. At the other extreme, Bangladesh had the world’s lowest ASPR (609.44 per 1,00,000) and ASDR (115.01 per 1,00,000) (Supplementary Tables S2–S5). From 1990 to 2021, more than 90% of countries experienced modest increases in ASPR and ASDR. The steepest declines were observed in the Cook Islands, Kuwait and the Syrian Arab Republic, whereas the Maldives, Equatorial Guinea and Qatar showed the largest increases (Figure 3; Supplementary Tables S2–S6).

### 3.6 Age-specific burden

In 1990, ASD caused 3.30 million DALYs among 15–39-year-olds. Nearly half of this burden was in adolescents aged 15–19 years (0.80 million; 24.1%) and young adults aged 20–24 years (0.74 million; 22.4%), forming an inverted-pyramid age distribution. Prevalence showed a similar pattern, with 17.51 million total cases in 1990 peaking in the 15–24-year age range. By 2021, the absolute burden had increased across all five age groups: DALYs reached 4.55 million and prevalence 24.13 million.

**Figure 4:**
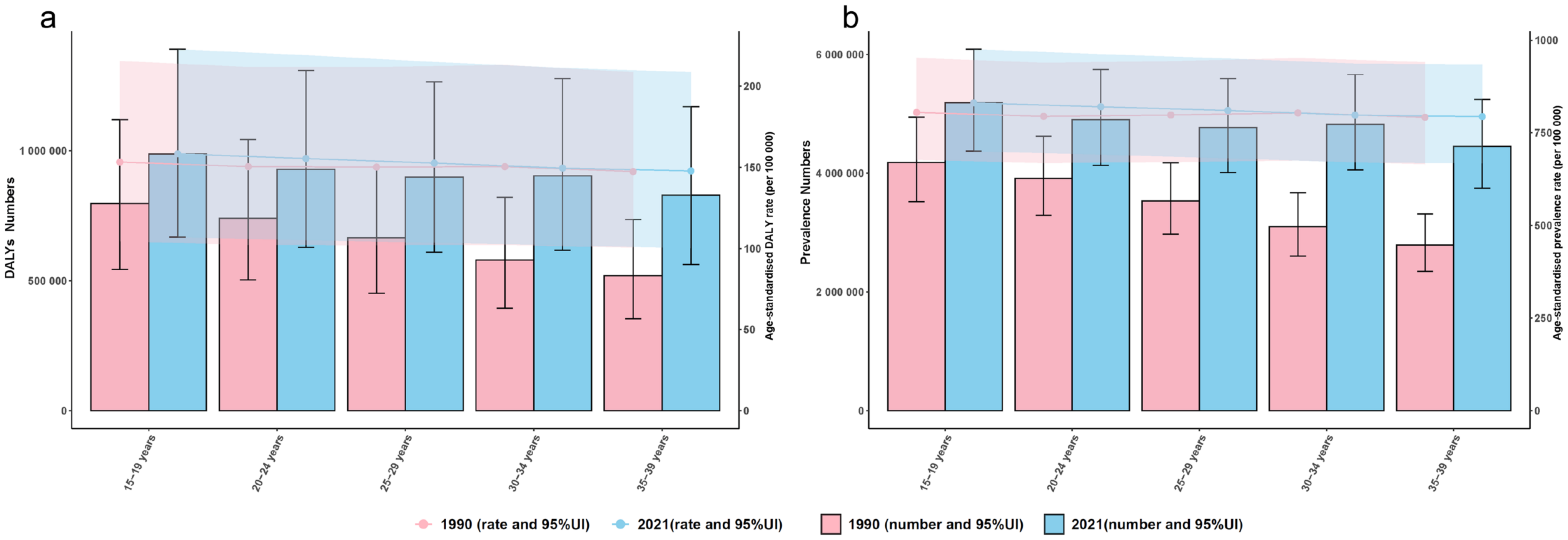
Age-specific burden of ASD among AYA, 1990 vs. 2021. **(a)** Disability-adjusted life years (DALYs, bars) and age-standardised DALY rate (ASDR, lines) by 5-year age group; **(b)** prevalent cases (bars) and age-standardised prevalence rate (ASPR, lines) for the same age strata. Shaded areas and error bars denote 95% uncertainty intervals. ASD, autism spectrum disorders; DALY, disability-adjusted life year; ASDR, age-standardised DALY rate; ASPR, age-standardised prevalence rate; AYA, adolescents and young adults. Shaded bands and error bars represent 95% uncertainty intervals (UI).

The 30–34-year age group recorded the sharpest increase from 1990 to 2021, with prevalence climbing from 3.10 million to 4.82 million (56%) and DALYs from 0.579 million to 0.904 million (56%). The 35–39-year group followed closely, with approximately a 60% increase in both metrics. Although 15–24-year-olds still accounted for the largest share of cases and DALYs in 2021 (15.08 million cases and 1.916 million DALYs), their proportional contribution fell from 46% in 1990 to 42% in 2021, flattening the age distribution. Notably, the age-standardised prevalence and DALY rates changed by less than 3% over the 32-year period (Figure 4).

### 3.7 Evolution of regional age profiles

**Figure 5:**
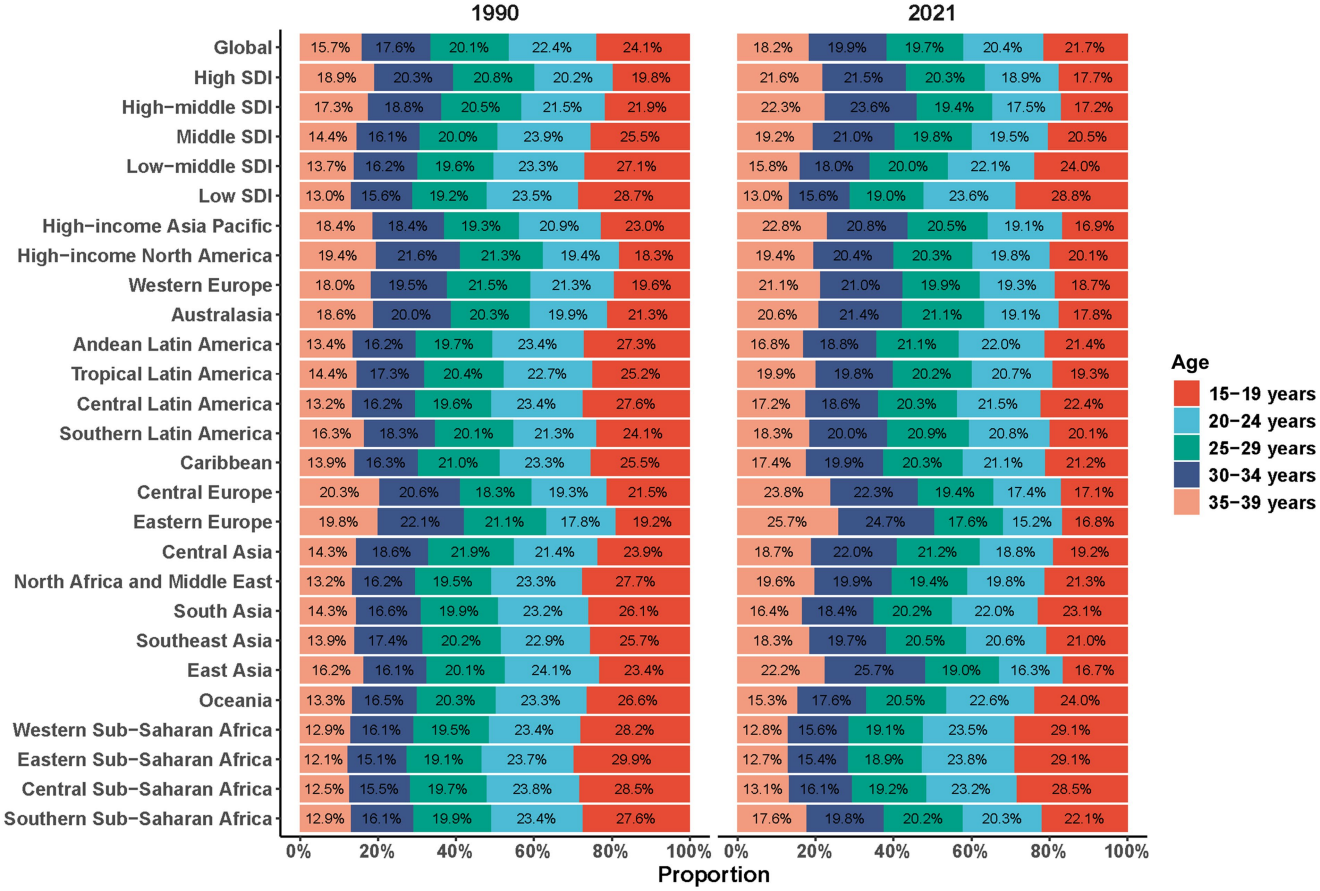
Proportional contribution of disability-adjusted life years by five age groups (15–19, 20–24, 25–29, 30–34, 35–39 years) for ASD among AYA, 1990 vs. 2021. ASD, autism spectrum disorders; AYA, adolescents and young adults.

Between 1990 and 2021, the absolute burden rose in every age group across all regions, yet three typical patterns emerged. High-income North America showed a “platform” pattern, with differences of <3 percentage points between age groups. Tropical Latin America’s age profile remained largely unchanged, with its peak burden consistently at 15–19 years. South Asia shifted from an inverted-pyramid shape in 1990 to a more “trapezoidal” profile by 2021, as the burden in the 30–34-year age group approximately doubled over the period. Despite these divergent regional patterns, the age-standardised DALY rate in 2021 remained within a narrow band of 145–160 per 1,00,000 across all SDI levels, underscoring the stability of per-person risk (Figure 5).

### 3.8 Cross-country inequality analysis

Across 204 countries, adolescent- and young-adult ASD DALY rates remained modestly but consistently concentrated in higher-SDI settings from 1990 to 2021. The SII was 20.30 (95% UI 10.37–30.24) per 1,00,000 in 1990 and 22.53 (95% UI 12.53–32.53) per 1,00,000 in 2021; overlapping UIs indicate that the absolute gap scarcely changed. The CIX likewise held steady at 0.04 (95% UI 0.02–0.05) in both years, signifying stable relative inequality. Overall, the disproportionate concentration of ASD-related DALYs in high-SDI countries has persisted for three decades (Supplementary Figure S1; Supplementary Table S7).

### 3.9 Frontier analysis

Stochastic-frontier assessment of all 204 countries (1990–2021) showed a gently descending frontier of age-standardised DALY rates (ASDR) with rising SDI (0–1). Yearly density shading progresses from light in 1990 to dark in 2021, indicating a gradual downward shift in the best-attainable burden. In 2021, substantial heterogeneity was evident: several high-SDI economies—Japan, the Republic of Korea, Singapore, Brunei, Australia, New Zealand, Canada, Chile, the United States, Uruguay, Ireland, Argentina, Iceland, Sweden and Spain—lay well above the frontier, with ASDRs exceeding levels predicted by their development status. By contrast, low-SDI countries such as Bangladesh, Somalia, Niger, Nepal and Haiti clustered on or below the frontier—a pattern that should be interpreted with caution, as measurement error and under-ascertainment in low-SDI settings may contribute to lower observed rates rather than genuinely lower burden. Overall, although the frontier itself fell slightly over time, many high-SDI nations remained “off-frontier,” indicating that their resource advantages have yet to translate into commensurately lower ASD disability (Supplementary Figure S2; Supplementary Table S8).

## 4 Discussion

ASD has become one of the most closely watched neurodevelopmental conditions worldwide. Although incidence has reportedly plateaued – or risen only modestly – in several high-income countries in recent years (15), geographical disparities remain pronounced (2). For AYA, the demands of education, employment and social integration converge with a high prevalence of psychiatric comorbidity (16–18). By providing a comprehensive description of the AYA ASD burden across 204 countries and territories from 1990 to 2021, our study provides evidence to inform needed to refine clinical management, epidemiological surveillance and public health strategy.

Over the past 32 years, the absolute number of AYA ASD cases and DALYs increased by approximately 38%, whereas the ASPR and ASDR fluctuated by less than 0.10% per year – a typical pattern of rising absolute burden despite a stable underlying risk. In 2021, the male ASPR and ASDR were approximately 2.10-fold higher than those observed in females, mirroring the latest systematic review of clinical diagnoses. This disparity is likely multifactorial, arising from a complex interplay of biological susceptibility and socio-diagnostic factors. On a biological level, emerging experimental evidence suggests that prenatal exposure to endocrine disruptors may heighten male neural vulnerability by suppressing aromatase activity, a pathway that may be partially reversible (19). Conversely, the phenomenon of ‘social camouflaging’—where females with ASD more frequently mask their social communication difficulties—often leads to underdiagnosis or delayed diagnosis, thereby artificially inflating the male-to-female ratio in prevalence estimates (20). Furthermore, historical diagnostic criteria have been predominantly built upon male presentations of ASD, potentially lacking sensitivity to the female phenotype. This underscores the critical need for sex-specific screening tools and support strategies to ensure timely and accurate diagnosis for all individuals.

SDI-stratified analyses revealed a clear gradient in 2021 – high SDI > high-middle SDI > middle SDI ≈ low-middle SDI > low SDI. High SDI settings recorded the highest ASPR (1090.72 per 1,00,000) and ASDR (205.00 per 1,00,000) yet showed virtually no decline over 32 years. Conversely, low SDI regions exhibited the lowest corresponding rates (845.15 and 158.57 per 1,00,000, respectively) but experienced a surge in absolute case counts, driven by large birth cohorts and expanding diagnostic infrastructure. Achieving precision across “sex, place and wealth” therefore demands tailored priorities: high-SDI countries should pivot from expanding detection to enhancing post-school education and employment support; middle- and low-SDI regions should embed community- and school-based screening and evidence-based parent training within primary care systems; and all jurisdictions should adopt a neurodiversity-affirming framework that places quality of life at the centre (21).

By 2021, India (4.53 million cases), China (3.12 million) and the United States (1.27 million) together accounted for more than one-third of global prevalent cases, whereas several high-income, small-population nations – Japan, the Republic of Korea and Singapore – topped the world in age-standardised rates (ASPR > 1,600 per 1,00,000; ASDR > 300 per 1,00,000). More than 90% of countries posted slight increases in these rates, but the pace of change diverged sharply: the Maldives, Equatorial Guinea and Qatar recorded the largest positive EAPCs, whereas the Cook Islands, Kuwait and the Syrian Arab Republic showed declines. The coexistence of “high rate/low population” and “low rate/high population” profiles means that resource allocation must balance per-capita service strain against absolute caseload (22). High-income nations, despite rapid case detection, have not expanded post-school support at a pace sufficient to curb ASDR; middle- and low-income settings, still building diagnostic systems, display “low rates but swelling absolute numbers” (1, 23). Policy responses should therefore differ: high-rate, small-population countries need stronger vocational and social inclusion programmes; populous nations should mainstream community screening and digital parent training; and fast-growing small states should form regional alliances to scale up low-cost early intervention models (24, 25).

Between 1990 and 2021, the peak functional loss attributable to ASD shifted from the 15–24-year age group to the 30–39-year group, echoing meta-analytic findings of increasing overall prevalence amid flat age-standardised rates (26). United States insurance and electronic medical record data show a dramatic rise in diagnoses among 26–34-year-olds between 2011 and 2022 (27); European and North American sentinel sites likewise document a late-youth surge in reporting without a parallel expansion of services, producing a “diagnosis first, support lagging” gap (28). Extending routine screening to include individuals at ages 25 and 39 and building lifelong stepped-care packages – covering vocational training, mental health care and comorbidity management – will be essential to offset the DALY plateau after age 30.

Previous global studies have focused on children or all-age populations, leaving the AYA “second peak” under-examined. Although Zeidan et al. estimated childhood ASD prevalence at approximately 1% and Salari et al. confirmed a rising overall burden, neither study disaggregated outcomes for the 15–39-year age range (29, 30). Leveraging GBD 2021, our analysis is the first to map AYA ASPR, ASDR and EAPC across 204 countries, effectively creating a cross-national map of AYA rates and trends to guide policy (i.e., highlighting the mismatch between “high rate/low population” and “low rate/high population” profiles across settings).

Using both SII and CIX, we found that adolescent- and young-adult ASD DALYs have remained disproportionately concentrated in high-SDI settings since 1990: the absolute gap rose only from 20.30 to 22.53 per 1,00,000 and overlapping UIs indicate no true narrowing, while the CIX stayed at approximately 0.04, confirming a persistent tilt of diagnostic and support systems toward affluent nations—echoing earlier GBD and systematic review evidence across all ages and in children (31). Frontier modelling reinforced this picture: although the “best-attainable” curve shifted slightly downward with increasing SDI, several high-income countries (e.g., Japan, Republic of Korea, Singapore, Australia, Canada, United States) still lay well above the frontier in 2021, whereas low-SDI economies such as Bangladesh and Somalia sat on or below it, a pattern that likely reflects under-ascertainment rather than genuinely lower burden (32, 33). This high-rate/high-resource mismatch implies that economic growth alone will not reduce ASD disability; adult screening, employment support and community interventions must be embedded within universal health coverage, while reinforced primary-level surveillance and parent training in low- and middle-SDI countries are needed to uncover hidden cases and achieve true global convergence. We adopted a half-normal composite-error specification to enforce non-negativity of inefficiency; in sensitivity analyses, exponential and truncated-normal alternatives yielded unchanged qualitative inferences.

Because GBD 2021 does not attribute ASD to specific risk factors, we synthesised high-quality evidence published in the past 5 years. For instance, gestational diabetes is associated with an approximately 40% higher risk of ASD in offspring (34); high PM₂.₅/NO₂ exposure is linked to ~31% higher risk (35); adherence to a Mediterranean diet is associated with about a 23% lower risk (36); and research on the BPA–aromatase–ASD pathway (with partial reversibility) suggests new targets for mitigating chemical exposures (19). However, important limitations of these studies include the concentration of cohorts in high-income countries, heterogeneous exposure windows and doses, and inconsistent case ascertainment. In addition, our outcome is ASD-attributable DALYs: psychiatric comorbidities common in AYA (e.g., anxiety and depression) are assigned to those disorders under GBD attribution rules and thus are not included in ASD DALYs. Consequently, our ASD estimates likely understate real-world burden in adolescents and young adults, consistent with recent evidence documenting high comorbidity rates in autistic populations (37). Precise risk attribution will require establishing longitudinal consortia in the global South that integrate environmental and biological data, enriched with multi-omics markers to support multi-layered prevention.

## 5 Conclusion

Between 1990 and 2021, the global absolute burden of ASD among adolescents and young adults increased by approximately 40%, whereas age-standardised rates showed little change, indicating that this rise reflects demographic growth and improved detection rather than increased individual risk. Notably, frontier-adherent or near-frontier ASD rates observed in some low-SDI settings should be interpreted primarily as signals of under-ascertainment and diagnostic/surveillance gaps rather than truly low underlying burden (23). The persistent male predominance (rate ratio ≈2.1) and pronounced rise in disability in the 30–39 age group highlight ongoing and emerging challenges across the lifespan. Given these disparities and significant cross-national inequalities (SII 22.53; CIX 0.04), context-sensitive strategies are needed. High-SDI countries could integrate adult screening, vocational support, and community-based care into health coverage; low- and middle-SDI regions may prioritize enhanced surveillance, school-community screening, and parent-training to improve identification. Adopting neurodiversity-affirming approaches that emphasize lifelong support and social integration is widely encouraged. Reducing global disparities in ASD care will require more efficient resource use in high-capacity settings and sustained investment in foundational capacity in underserved areas.

## Data Availability

The original contributions presented in the study are included in the article/Supplementary material, further inquiries can be directed to the corresponding author.
