# Supplementary material for: Global burden, inequality, and frontier gaps of autism spectrum disorder disability in adolescents and young adults, 1990–2021: a systematic analysis of the GBD 2021 study
Source: Front Public Health. 2025 Oct 16;13:1681565. doi: 10.3389/fpubh.2025.1681565 (PMC12571915; doi:10.3389/fpubh.2025.1681565)
Supplement: Supplementary file 1 [file Data_Sheet_1.docx]

**Supplementary material**


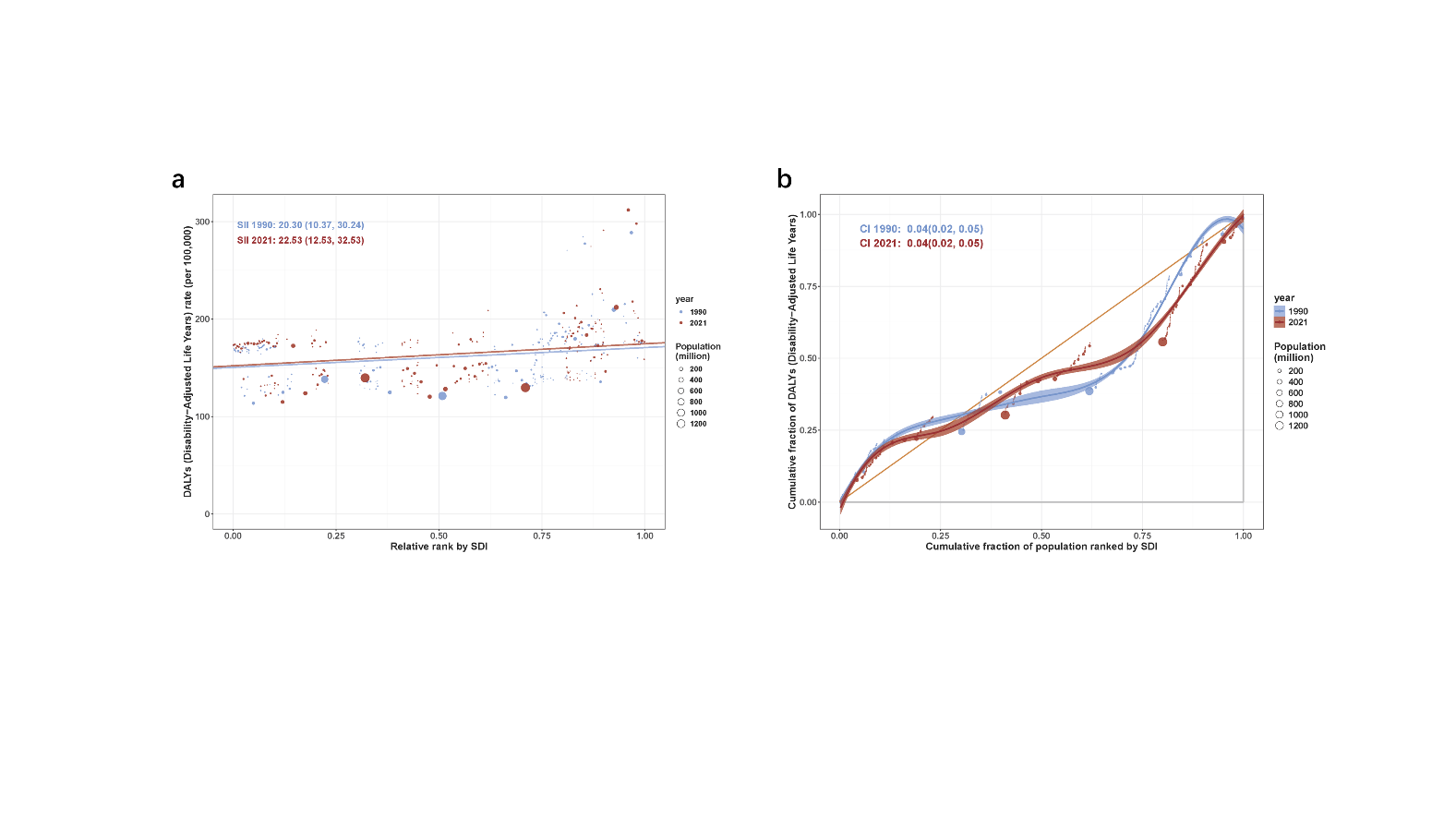


**Supplementary Figure S1.** Population-weighted rank-regression (Slope Index of Inequality, panel A) and concentration curves (panel B) for age-standardised ASD DALY rates in adolescents and young adults by Socio-demographic Index, 1990 and 2021. *DALY = disability-adjusted life-year; SDI = Socio-demographic Index.*


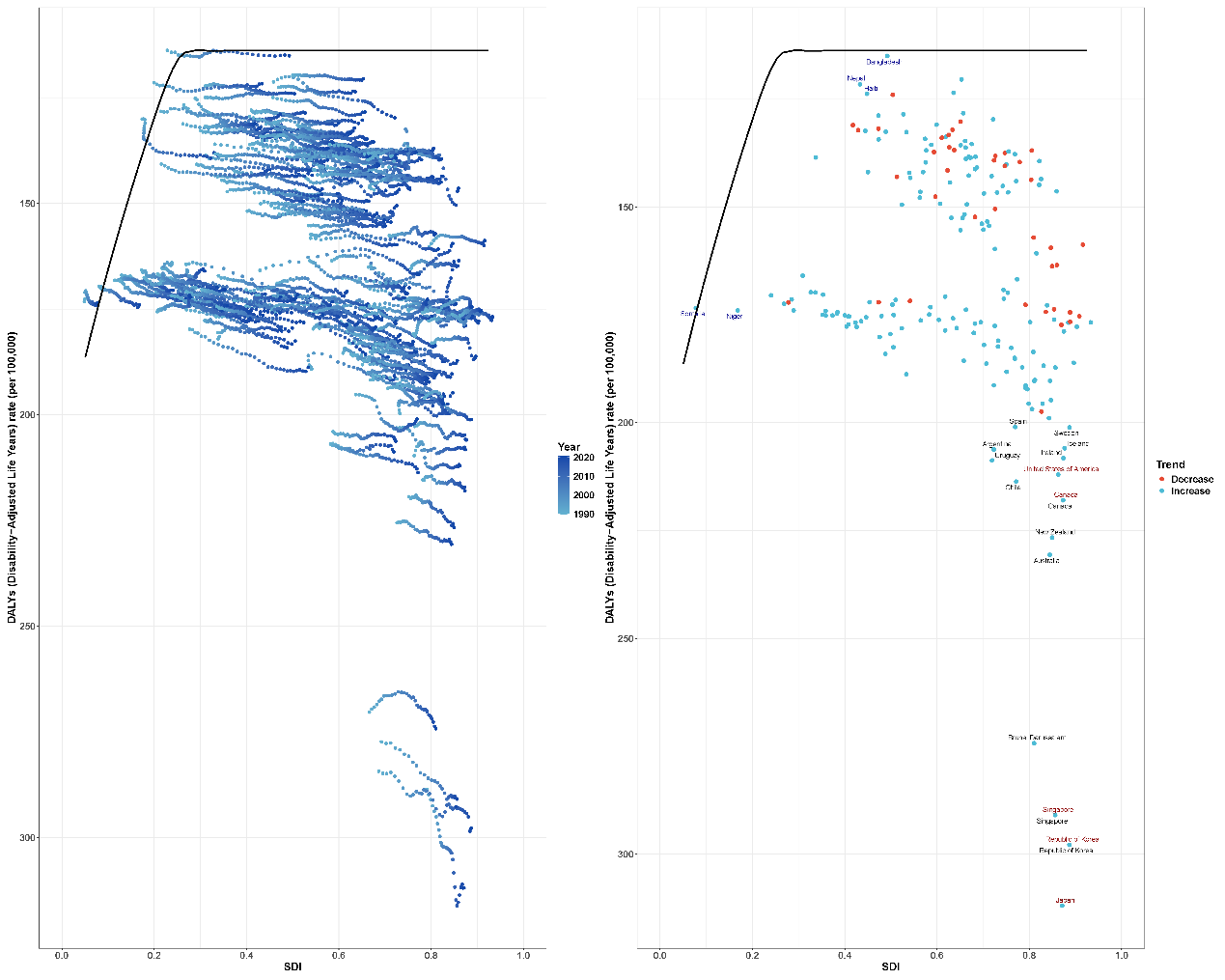
 **Supplementary Figure S2. Stochastic-frontier analysis of age-standardised ASD DALY rates versus SDI.**• Panel A shows the time-series frontier (solid black line) with yearly density shading (light = 1990; dark = 2021), indicating a gradual downward shift in the best-attainable burden.
• Panel B plots each country in 2021; the 15 countries furthest above the frontier are labelled in black. Dots coloured red (high-SDI, large gap) or blue (low-SDI, minimal gap) highlight under- and over-performance relative to development. Arrow colours denote national change in DALY rate between 1990 and 2021 (green = decrease, orange = increase). *DALY = disability-adjusted life-year; SDI = Socio-demographic Index.*

**Table S1.** Global ASD burden among adolescents and young adults, 1990–2021: numbers and age-standardised rates of DALYs and prevalence by sex.

| **Measure** | **Location** | **Sex** | **Age** | **Cause** | **Metric** | **Year** | **Val** | **Upper** | **Lower** |
| --- | --- | --- | --- | --- | --- | --- | --- | --- | --- |
| DALYs | Global | Male | Age-standardized | Autism spectrum disorders | Rate | 1990 | 204.34 | 139.65 | 287.86 |
| DALYs | Global | Female | Age-standardized | Autism spectrum disorders | Rate | 1990 | 95.37 | 65.57 | 134.74 |
| DALYs | Global | Both | Age-standardized | Autism spectrum disorders | Rate | 1990 | 150.49 | 102.43 | 212.28 |
| DALYs | Global | Male | 15-39 years | Autism spectrum disorders | Number | 1990 | 2266549.99 | 3190069.86 | 1548642.32 |
| DALYs | Global | Female | 15-39 years | Autism spectrum disorders | Number | 1990 | 1034226.88 | 1458909.62 | 709973.05 |
| DALYs | Global | Both | 15-39 years | Autism spectrum disorders | Number | 1990 | 3300776.87 | 4654828.48 | 2242510.64 |
| DALYs | Global | Male | Age-standardized | Autism spectrum disorders | Rate | 2021 | 205.98 | 140.38 | 290.83 |
| DALYs | Global | Female | Age-standardized | Autism spectrum disorders | Rate | 2021 | 98.4 | 67.33 | 138.76 |
| DALYs | Global | Both | Age-standardized | Autism spectrum disorders | Rate | 2021 | 153 | 103.77 | 215.64 |
| DALYs | Global | Male | 15-39 years | Autism spectrum disorders | Number | 2021 | 3108150.79 | 4400144.59 | 2115017.9 |
| DALYs | Global | Female | 15-39 years | Autism spectrum disorders | Number | 2021 | 1439310.35 | 2033299.41 | 985289.87 |
| DALYs | Global | Both | 15-39 years | Autism spectrum disorders | Number | 2021 | 4547461.14 | 6410493.83 | 3083617.95 |
| DALYs | Global | Male | 15-39 years | Autism spectrum disorders | Rate | 1990 | 204.48 | 287.79 | 139.71 |
| DALYs | Global | Female | 15-39 years | Autism spectrum disorders | Rate | 1990 | 95.47 | 134.67 | 65.54 |
| DALYs | Global | Both | 15-39 years | Autism spectrum disorders | Rate | 1990 | 150.60 | 212.37 | 102.31 |
| DALYs | Global | Male | 15-39 years | Autism spectrum disorders | Rate | 2021 | 205.88 | 291.46 | 140.09 |
| DALYs | Global | Female | 15-39 years | Autism spectrum disorders | Rate | 2021 | 98.24 | 138.78 | 67.25 |
| DALYs | Global | Both | 15-39 years | Autism spectrum disorders | Rate | 2021 | 152.86 | 215.49 | 103.66 |
| Prevalence | Global | Male | Age-standardized | Autism spectrum disorders | Rate | 1990 | 1079.86 | 909.54 | 1275.04 |
| Prevalence | Global | Female | Age-standardized | Autism spectrum disorders | Rate | 1990 | 511.65 | 426.86 | 608.8 |
| Prevalence | Global | Both | Age-standardized | Autism spectrum disorders | Rate | 1990 | 799.04 | 672.13 | 945.55 |
| Prevalence | Global | Male | 15-39 years | Autism spectrum disorders | Number | 1990 | 11970707.05 | 14129590.8 | 10080976.45 |
| Prevalence | Global | Female | 15-39 years | Autism spectrum disorders | Number | 1990 | 5544427.11 | 6598055.48 | 4627825.75 |
| Prevalence | Global | Both | 15-39 years | Autism spectrum disorders | Number | 1990 | 17515134.16 | 20719335.39 | 14741746.93 |
| Prevalence | Global | Male | Age-standardized | Autism spectrum disorders | Rate | 2021 | 1086.79 | 917.38 | 1271.8 |
| Prevalence | Global | Female | Age-standardized | Autism spectrum disorders | Rate | 2021 | 528.1 | 441.21 | 627.57 |
| Prevalence | Global | Both | Age-standardized | Autism spectrum disorders | Rate | 2021 | 811.67 | 683.34 | 952.87 |
| Prevalence | Global | Male | 15-39 years | Autism spectrum disorders | Number | 2021 | 16403053.8 | 19196491.73 | 13838920.58 |
| Prevalence | Global | Female | 15-39 years | Autism spectrum disorders | Number | 2021 | 7727919.93 | 9180412.06 | 6455282.83 |
| Prevalence | Global | Both | 15-39 years | Autism spectrum disorders | Number | 2021 | 24130973.74 | 28329702.55 | 20300728.74 |
| Prevalence | Global | Male | 15-39 years | Autism spectrum disorders | Rate | 1990 | 1079.94 | 1274.70 | 909.46 |
| Prevalence | Global | Female | 15-39 years | Autism spectrum disorders | Rate | 1990 | 511.79 | 609.05 | 427.18 |
| Prevalence | Global | Both | 15-39 years | Autism spectrum disorders | Rate | 1990 | 799.12 | 945.31 | 672.59 |
| Prevalence | Global | Male | 15-39 years | Autism spectrum disorders | Rate | 2021 | 1086.51 | 1271.54 | 916.66 |
| Prevalence | Global | Female | 15-39 years | Autism spectrum disorders | Rate | 2021 | 527.46 | 626.60 | 440.60 |
| Prevalence | Global | Both | 15-39 years | Autism spectrum disorders | Rate | 2021 | 811.17 | 952.31 | 682.42 |

**Table S2.** Number of ASD DALYs in adolescents and young adults by country, 2021.

| **measure** | **location** | **sex** | **age** | **metric** | **year** | **val** | **upper** | **lower** |
| --- | --- | --- | --- | --- | --- | --- | --- | --- |
| DALYs | Afghanistan | Both | 15-39 years | Number | 2021 | 17063.25 | 24160.09 | 11787.51 |
| DALYs | Albania | Both | 15-39 years | Number | 2021 | 1765.18 | 2473.60 | 1201.33 |
| DALYs | Algeria | Both | 15-39 years | Number | 2021 | 25765.44 | 36375.48 | 17344.05 |
| DALYs | American Samoa | Both | 15-39 years | Number | 2021 | 24.40 | 34.56 | 16.73 |
| DALYs | Andorra | Both | 15-39 years | Number | 2021 | 45.02 | 63.42 | 30.67 |
| DALYs | Angola | Both | 15-39 years | Number | 2021 | 21064.14 | 29157.28 | 14631.94 |
| DALYs | Antigua and Barbuda | Both | 15-39 years | Number | 2021 | 48.11 | 67.31 | 32.76 |
| DALYs | Argentina | Both | 15-39 years | Number | 2021 | 36101.81 | 50435.96 | 24694.03 |
| DALYs | Armenia | Both | 15-39 years | Number | 2021 | 1944.10 | 2742.04 | 1338.08 |
| DALYs | Australia | Both | 15-39 years | Number | 2021 | 19931.65 | 28254.56 | 13882.37 |
| DALYs | Austria | Both | 15-39 years | Number | 2021 | 4956.06 | 6993.99 | 3334.88 |
| DALYs | Azerbaijan | Both | 15-39 years | Number | 2021 | 7452.30 | 10363.55 | 5187.81 |
| DALYs | Bahamas | Both | 15-39 years | Number | 2021 | 211.69 | 299.26 | 145.44 |
| DALYs | Bahrain | Both | 15-39 years | Number | 2021 | 1198.95 | 1658.33 | 834.93 |
| DALYs | Bangladesh | Both | 15-39 years | Number | 2021 | 79181.41 | 112227.38 | 53668.86 |
| DALYs | Barbados | Both | 15-39 years | Number | 2021 | 138.51 | 195.07 | 94.87 |
| DALYs | Belarus | Both | 15-39 years | Number | 2021 | 5456.33 | 7605.80 | 3775.49 |
| DALYs | Belgium | Both | 15-39 years | Number | 2021 | 6070.15 | 8423.82 | 4105.33 |
| DALYs | Belize | Both | 15-39 years | Number | 2021 | 253.46 | 354.89 | 170.30 |
| DALYs | Benin | Both | 15-39 years | Number | 2021 | 9229.64 | 12932.38 | 6384.21 |
| DALYs | Bermuda | Both | 15-39 years | Number | 2021 | 25.29 | 35.53 | 16.84 |
| DALYs | Bhutan | Both | 15-39 years | Number | 2021 | 446.37 | 622.89 | 310.52 |
| DALYs | Bolivia (Plurinational State of) | Both | 15-39 years | Number | 2021 | 6438.90 | 9134.76 | 4456.35 |
| DALYs | Bosnia and Herzegovina | Both | 15-39 years | Number | 2021 | 1920.64 | 2713.56 | 1336.30 |
| DALYs | Botswana | Both | 15-39 years | Number | 2021 | 1893.70 | 2641.27 | 1320.09 |
| DALYs | Brazil | Both | 15-39 years | Number | 2021 | 102412.17 | 143607.48 | 68919.32 |
| DALYs | Brunei Darussalam | Both | 15-39 years | Number | 2021 | 558.57 | 788.17 | 381.88 |
| DALYs | Bulgaria | Both | 15-39 years | Number | 2021 | 3499.51 | 4971.44 | 2419.65 |
| DALYs | Burkina Faso | Both | 15-39 years | Number | 2021 | 14914.09 | 21082.71 | 10336.61 |
| DALYs | Burundi | Both | 15-39 years | Number | 2021 | 9207.98 | 13082.03 | 6249.49 |
| DALYs | Cabo Verde | Both | 15-39 years | Number | 2021 | 472.69 | 667.64 | 321.84 |
| DALYs | Cambodia | Both | 15-39 years | Number | 2021 | 9726.73 | 13761.29 | 6590.35 |
| DALYs | Cameroon | Both | 15-39 years | Number | 2021 | 22706.93 | 31821.19 | 15556.95 |
| DALYs | Canada | Both | 15-39 years | Number | 2021 | 25778.42 | 36692.37 | 17955.90 |
| DALYs | Central African Republic | Both | 15-39 years | Number | 2021 | 3649.22 | 5155.22 | 2461.68 |
| DALYs | Chad | Both | 15-39 years | Number | 2021 | 10827.49 | 15280.67 | 7392.10 |
| DALYs | Chile | Both | 15-39 years | Number | 2021 | 15083.87 | 21500.49 | 10383.39 |
| DALYs | China | Both | 15-39 years | Number | 2021 | 595290.82 | 839048.35 | 401171.36 |
| DALYs | Colombia | Both | 15-39 years | Number | 2021 | 30642.24 | 43416.16 | 20819.46 |
| DALYs | Comoros | Both | 15-39 years | Number | 2021 | 559.10 | 788.91 | 380.53 |
| DALYs | Congo | Both | 15-39 years | Number | 2021 | 3885.77 | 5461.29 | 2685.70 |
| DALYs | Cook Islands | Both | 15-39 years | Number | 2021 | 8.24 | 11.58 | 5.64 |
| DALYs | Costa Rica | Both | 15-39 years | Number | 2021 | 2948.36 | 4195.90 | 1993.55 |
| DALYs | Croatia | Both | 15-39 years | Number | 2021 | 2434.82 | 3472.39 | 1675.79 |
| DALYs | Cuba | Both | 15-39 years | Number | 2021 | 5107.90 | 7146.30 | 3469.77 |
| DALYs | Cyprus | Both | 15-39 years | Number | 2021 | 869.33 | 1222.35 | 579.59 |
| DALYs | Czechia | Both | 15-39 years | Number | 2021 | 5755.96 | 8115.51 | 4015.76 |
| DALYs | Côte d'Ivoire | Both | 15-39 years | Number | 2021 | 19993.43 | 28007.35 | 13977.80 |
| DALYs | Democratic People's Republic of Korea | Both | 15-39 years | Number | 2021 | 14264.97 | 20068.75 | 9892.34 |
| DALYs | Democratic Republic of the Congo | Both | 15-39 years | Number | 2021 | 63352.80 | 90264.34 | 43334.38 |
| DALYs | Denmark | Both | 15-39 years | Number | 2021 | 3392.45 | 4713.52 | 2292.25 |
| DALYs | Djibouti | Both | 15-39 years | Number | 2021 | 994.74 | 1413.22 | 684.48 |
| DALYs | Dominica | Both | 15-39 years | Number | 2021 | 35.61 | 50.63 | 24.97 |
| DALYs | Dominican Republic | Both | 15-39 years | Number | 2021 | 6081.52 | 8379.02 | 4191.85 |
| DALYs | Ecuador | Both | 15-39 years | Number | 2021 | 9842.85 | 13687.66 | 6550.41 |
| DALYs | Egypt | Both | 15-39 years | Number | 2021 | 63056.21 | 89993.25 | 42348.03 |
| DALYs | El Salvador | Both | 15-39 years | Number | 2021 | 3807.62 | 5367.65 | 2567.19 |
| DALYs | Equatorial Guinea | Both | 15-39 years | Number | 2021 | 1301.54 | 1868.25 | 893.93 |
| DALYs | Eritrea | Both | 15-39 years | Number | 2021 | 4990.72 | 7069.82 | 3423.54 |
| DALYs | Estonia | Both | 15-39 years | Number | 2021 | 750.04 | 1033.66 | 511.73 |
| DALYs | Eswatini | Both | 15-39 years | Number | 2021 | 884.99 | 1224.78 | 613.22 |
| DALYs | Ethiopia | Both | 15-39 years | Number | 2021 | 81743.40 | 114047.63 | 57258.37 |
| DALYs | Fiji | Both | 15-39 years | Number | 2021 | 482.93 | 683.93 | 333.68 |
| DALYs | Finland | Both | 15-39 years | Number | 2021 | 2717.02 | 3840.43 | 1867.78 |
| DALYs | France | Both | 15-39 years | Number | 2021 | 34282.18 | 48078.09 | 23469.88 |
| DALYs | Gabon | Both | 15-39 years | Number | 2021 | 1321.07 | 1837.76 | 892.74 |
| DALYs | Gambia | Both | 15-39 years | Number | 2021 | 1763.57 | 2455.63 | 1241.76 |
| DALYs | Georgia | Both | 15-39 years | Number | 2021 | 2044.32 | 2918.09 | 1413.84 |
| DALYs | Germany | Both | 15-39 years | Number | 2021 | 44829.08 | 62817.19 | 30809.34 |
| DALYs | Ghana | Both | 15-39 years | Number | 2021 | 25285.25 | 35448.97 | 17182.76 |
| DALYs | Greece | Both | 15-39 years | Number | 2021 | 4790.28 | 6734.58 | 3289.13 |
| DALYs | Greenland | Both | 15-39 years | Number | 2021 | 40.22 | 56.34 | 27.61 |
| DALYs | Grenada | Both | 15-39 years | Number | 2021 | 56.01 | 78.43 | 38.50 |
| DALYs | Guam | Both | 15-39 years | Number | 2021 | 79.70 | 111.83 | 54.40 |
| DALYs | Guatemala | Both | 15-39 years | Number | 2021 | 9713.16 | 13616.66 | 6686.47 |
| DALYs | Guinea | Both | 15-39 years | Number | 2021 | 8835.03 | 12305.50 | 6132.56 |
| DALYs | Guinea-Bissau | Both | 15-39 years | Number | 2021 | 1444.80 | 2004.63 | 990.54 |
| DALYs | Guyana | Both | 15-39 years | Number | 2021 | 405.74 | 561.71 | 272.82 |
| DALYs | Haiti | Both | 15-39 years | Number | 2021 | 6801.52 | 9584.76 | 4683.53 |
| DALYs | Honduras | Both | 15-39 years | Number | 2021 | 6314.11 | 9001.24 | 4349.53 |
| DALYs | Hungary | Both | 15-39 years | Number | 2021 | 5263.90 | 7275.10 | 3572.04 |
| DALYs | Iceland | Both | 15-39 years | Number | 2021 | 246.25 | 342.73 | 170.69 |
| DALYs | India | Both | 15-39 years | Number | 2021 | 851742.20 | 1192360.05 | 581243.70 |
| DALYs | Indonesia | Both | 15-39 years | Number | 2021 | 145942.30 | 207245.88 | 98153.23 |
| DALYs | Iran (Islamic Republic of) | Both | 15-39 years | Number | 2021 | 53138.92 | 75107.70 | 35915.22 |
| DALYs | Iraq | Both | 15-39 years | Number | 2021 | 24255.65 | 33813.84 | 16659.48 |
| DALYs | Ireland | Both | 15-39 years | Number | 2021 | 3250.04 | 4469.30 | 2237.07 |
| DALYs | Israel | Both | 15-39 years | Number | 2021 | 5220.42 | 7324.62 | 3572.17 |
| DALYs | Italy | Both | 15-39 years | Number | 2021 | 31002.31 | 43453.06 | 21202.00 |
| DALYs | Jamaica | Both | 15-39 years | Number | 2021 | 1650.40 | 2316.92 | 1158.71 |
| DALYs | Japan | Both | 15-39 years | Number | 2021 | 100871.55 | 142013.06 | 69984.77 |
| DALYs | Jordan | Both | 15-39 years | Number | 2021 | 8581.48 | 12009.66 | 5822.40 |
| DALYs | Kazakhstan | Both | 15-39 years | Number | 2021 | 12333.25 | 17705.67 | 8547.85 |
| DALYs | Kenya | Both | 15-39 years | Number | 2021 | 38758.19 | 54397.79 | 26570.35 |
| DALYs | Kiribati | Both | 15-39 years | Number | 2021 | 64.00 | 89.89 | 43.40 |
| DALYs | Kuwait | Both | 15-39 years | Number | 2021 | 3358.98 | 4711.03 | 2249.72 |
| DALYs | Kyrgyzstan | Both | 15-39 years | Number | 2021 | 4791.73 | 6842.45 | 3247.11 |
| DALYs | Lao People's Democratic Republic | Both | 15-39 years | Number | 2021 | 4254.74 | 6077.34 | 2855.76 |
| DALYs | Latvia | Both | 15-39 years | Number | 2021 | 1001.52 | 1415.22 | 692.03 |
| DALYs | Lebanon | Both | 15-39 years | Number | 2021 | 3959.56 | 5548.67 | 2641.44 |
| DALYs | Lesotho | Both | 15-39 years | Number | 2021 | 1433.30 | 2002.01 | 992.04 |
| DALYs | Liberia | Both | 15-39 years | Number | 2021 | 3943.25 | 5510.83 | 2722.10 |
| DALYs | Libya | Both | 15-39 years | Number | 2021 | 4507.95 | 6413.21 | 3029.42 |
| DALYs | Lithuania | Both | 15-39 years | Number | 2021 | 1502.43 | 2103.46 | 1039.35 |
| DALYs | Luxembourg | Both | 15-39 years | Number | 2021 | 388.54 | 539.72 | 259.94 |
| DALYs | Madagascar | Both | 15-39 years | Number | 2021 | 20604.62 | 28715.89 | 14147.79 |
| DALYs | Malawi | Both | 15-39 years | Number | 2021 | 14377.53 | 20180.99 | 9767.43 |
| DALYs | Malaysia | Both | 15-39 years | Number | 2021 | 20168.37 | 28193.72 | 13635.76 |
| DALYs | Maldives | Both | 15-39 years | Number | 2021 | 408.91 | 578.64 | 271.80 |
| DALYs | Mali | Both | 15-39 years | Number | 2021 | 15499.07 | 21707.84 | 10733.88 |
| DALYs | Malta | Both | 15-39 years | Number | 2021 | 235.97 | 333.32 | 156.10 |
| DALYs | Marshall Islands | Both | 15-39 years | Number | 2021 | 31.86 | 44.90 | 22.08 |
| DALYs | Mauritania | Both | 15-39 years | Number | 2021 | 3075.89 | 4347.30 | 2094.53 |
| DALYs | Mauritius | Both | 15-39 years | Number | 2021 | 649.69 | 913.10 | 438.52 |
| DALYs | Mexico | Both | 15-39 years | Number | 2021 | 76955.72 | 108786.30 | 51818.95 |
| DALYs | Micronesia (Federated States of) | Both | 15-39 years | Number | 2021 | 57.76 | 81.10 | 39.45 |
| DALYs | Monaco | Both | 15-39 years | Number | 2021 | 16.29 | 22.67 | 11.04 |
| DALYs | Mongolia | Both | 15-39 years | Number | 2021 | 2147.94 | 3021.14 | 1483.00 |
| DALYs | Montenegro | Both | 15-39 years | Number | 2021 | 394.60 | 555.12 | 269.85 |
| DALYs | Morocco | Both | 15-39 years | Number | 2021 | 21689.14 | 30297.41 | 14767.65 |
| DALYs | Mozambique | Both | 15-39 years | Number | 2021 | 20558.67 | 28965.31 | 14323.94 |
| DALYs | Myanmar | Both | 15-39 years | Number | 2021 | 29856.80 | 42911.43 | 20248.28 |
| DALYs | Namibia | Both | 15-39 years | Number | 2021 | 1871.48 | 2656.31 | 1268.86 |
| DALYs | Nauru | Both | 15-39 years | Number | 2021 | 6.22 | 8.73 | 4.22 |
| DALYs | Nepal | Both | 15-39 years | Number | 2021 | 16381.40 | 23034.46 | 11248.86 |
| DALYs | Netherlands | Both | 15-39 years | Number | 2021 | 9315.06 | 12986.15 | 6434.65 |
| DALYs | New Zealand | Both | 15-39 years | Number | 2021 | 4077.29 | 5762.02 | 2839.93 |
| DALYs | Nicaragua | Both | 15-39 years | Number | 2021 | 4256.97 | 5946.02 | 2849.69 |
| DALYs | Niger | Both | 15-39 years | Number | 2021 | 15621.67 | 21947.77 | 10568.17 |
| DALYs | Nigeria | Both | 15-39 years | Number | 2021 | 156151.14 | 220612.50 | 107715.96 |
| DALYs | Niue | Both | 15-39 years | Number | 2021 | 0.79 | 1.09 | 0.54 |
| DALYs | North Macedonia | Both | 15-39 years | Number | 2021 | 1434.23 | 2013.58 | 996.13 |
| DALYs | Northern Mariana Islands | Both | 15-39 years | Number | 2021 | 23.73 | 32.88 | 16.33 |
| DALYs | Norway | Both | 15-39 years | Number | 2021 | 2812.43 | 3989.22 | 1941.02 |
| DALYs | Oman | Both | 15-39 years | Number | 2021 | 3898.83 | 5429.01 | 2604.45 |
| DALYs | Pakistan | Both | 15-39 years | Number | 2021 | 123046.80 | 172762.64 | 84554.56 |
| DALYs | Palau | Both | 15-39 years | Number | 2021 | 8.63 | 12.22 | 5.81 |
| DALYs | Palestine | Both | 15-39 years | Number | 2021 | 3341.49 | 4706.03 | 2281.54 |
| DALYs | Panama | Both | 15-39 years | Number | 2021 | 2532.87 | 3546.04 | 1733.46 |
| DALYs | Papua New Guinea | Both | 15-39 years | Number | 2021 | 5620.29 | 7902.37 | 3894.44 |
| DALYs | Paraguay | Both | 15-39 years | Number | 2021 | 3780.25 | 5204.38 | 2576.42 |
| DALYs | Peru | Both | 15-39 years | Number | 2021 | 20234.92 | 28334.04 | 13771.69 |
| DALYs | Philippines | Both | 15-39 years | Number | 2021 | 64183.11 | 90310.38 | 43351.86 |
| DALYs | Poland | Both | 15-39 years | Number | 2021 | 22882.26 | 31793.62 | 15886.62 |
| DALYs | Portugal | Both | 15-39 years | Number | 2021 | 4979.93 | 6921.95 | 3384.58 |
| DALYs | Puerto Rico | Both | 15-39 years | Number | 2021 | 1483.00 | 2071.40 | 1013.45 |
| DALYs | Qatar | Both | 15-39 years | Number | 2021 | 3267.47 | 4578.92 | 2234.63 |
| DALYs | Republic of Korea | Both | 15-39 years | Number | 2021 | 47562.54 | 66186.84 | 32667.66 |
| DALYs | Republic of Moldova | Both | 15-39 years | Number | 2021 | 2248.95 | 3203.72 | 1534.10 |
| DALYs | Romania | Both | 15-39 years | Number | 2021 | 9956.45 | 13827.70 | 6863.58 |
| DALYs | Russian Federation | Both | 15-39 years | Number | 2021 | 84734.62 | 119086.14 | 58331.65 |
| DALYs | Rwanda | Both | 15-39 years | Number | 2021 | 10045.60 | 13889.86 | 6861.11 |
| DALYs | Saint Kitts and Nevis | Both | 15-39 years | Number | 2021 | 31.10 | 44.20 | 21.43 |
| DALYs | Saint Lucia | Both | 15-39 years | Number | 2021 | 91.40 | 129.77 | 60.95 |
| DALYs | Saint Vincent and the Grenadines | Both | 15-39 years | Number | 2021 | 56.52 | 79.64 | 38.50 |
| DALYs | Samoa | Both | 15-39 years | Number | 2021 | 110.61 | 154.64 | 75.03 |
| DALYs | San Marino | Both | 15-39 years | Number | 2021 | 15.61 | 21.68 | 10.71 |
| DALYs | Sao Tome and Principe | Both | 15-39 years | Number | 2021 | 166.30 | 236.99 | 112.92 |
| DALYs | Saudi Arabia | Both | 15-39 years | Number | 2021 | 29706.18 | 42254.33 | 20395.36 |
| DALYs | Senegal | Both | 15-39 years | Number | 2021 | 11496.72 | 16066.43 | 8016.59 |
| DALYs | Serbia | Both | 15-39 years | Number | 2021 | 5661.30 | 7849.89 | 3853.44 |
| DALYs | Seychelles | Both | 15-39 years | Number | 2021 | 56.30 | 79.92 | 38.69 |
| DALYs | Sierra Leone | Both | 15-39 years | Number | 2021 | 6520.52 | 9262.84 | 4488.37 |
| DALYs | Singapore | Both | 15-39 years | Number | 2021 | 5564.94 | 7702.08 | 3801.68 |
| DALYs | Slovakia | Both | 15-39 years | Number | 2021 | 3243.30 | 4563.13 | 2243.02 |
| DALYs | Slovenia | Both | 15-39 years | Number | 2021 | 1129.78 | 1571.23 | 760.62 |
| DALYs | Solomon Islands | Both | 15-39 years | Number | 2021 | 363.03 | 510.26 | 243.67 |
| DALYs | Somalia | Both | 15-39 years | Number | 2021 | 14488.19 | 20383.84 | 9995.66 |
| DALYs | South Africa | Both | 15-39 years | Number | 2021 | 43326.27 | 60950.58 | 29611.15 |
| DALYs | South Sudan | Both | 15-39 years | Number | 2021 | 6272.60 | 8817.78 | 4408.06 |
| DALYs | Spain | Both | 15-39 years | Number | 2021 | 24876.81 | 34180.64 | 17153.91 |
| DALYs | Sri Lanka | Both | 15-39 years | Number | 2021 | 11841.75 | 16586.52 | 8099.20 |
| DALYs | Sudan | Both | 15-39 years | Number | 2021 | 26619.14 | 37432.71 | 18035.30 |
| DALYs | Suriname | Both | 15-39 years | Number | 2021 | 283.54 | 395.36 | 191.44 |
| DALYs | Sweden | Both | 15-39 years | Number | 2021 | 6506.30 | 9160.39 | 4476.28 |
| DALYs | Switzerland | Both | 15-39 years | Number | 2021 | 4888.37 | 6855.58 | 3421.35 |
| DALYs | Syrian Arab Republic | Both | 15-39 years | Number | 2021 | 7351.14 | 10509.96 | 5066.37 |
| DALYs | Taiwan (Province of China) | Both | 15-39 years | Number | 2021 | 13446.16 | 18920.77 | 9275.90 |
| DALYs | Tajikistan | Both | 15-39 years | Number | 2021 | 7165.34 | 10138.61 | 4921.13 |
| DALYs | Thailand | Both | 15-39 years | Number | 2021 | 29861.58 | 42461.84 | 20531.38 |
| DALYs | Timor-Leste | Both | 15-39 years | Number | 2021 | 761.62 | 1084.48 | 536.69 |
| DALYs | Togo | Both | 15-39 years | Number | 2021 | 5930.41 | 8232.18 | 4104.10 |
| DALYs | Tokelau | Both | 15-39 years | Number | 2021 | 0.70 | 0.99 | 0.48 |
| DALYs | Tonga | Both | 15-39 years | Number | 2021 | 53.24 | 75.68 | 36.21 |
| DALYs | Trinidad and Tobago | Both | 15-39 years | Number | 2021 | 683.16 | 962.15 | 462.95 |
| DALYs | Tunisia | Both | 15-39 years | Number | 2021 | 6605.66 | 9270.81 | 4455.57 |
| DALYs | Turkmenistan | Both | 15-39 years | Number | 2021 | 3682.05 | 5239.12 | 2509.14 |
| DALYs | Tuvalu | Both | 15-39 years | Number | 2021 | 6.81 | 9.65 | 4.59 |
| DALYs | Turkey | Both | 15-39 years | Number | 2021 | 49129.23 | 68827.17 | 33637.07 |
| DALYs | Uganda | Both | 15-39 years | Number | 2021 | 30668.53 | 43037.02 | 21504.13 |
| DALYs | Ukraine | Both | 15-39 years | Number | 2021 | 24980.82 | 34965.38 | 17027.44 |
| DALYs | United Arab Emirates | Both | 15-39 years | Number | 2021 | 6835.04 | 9612.56 | 4638.03 |
| DALYs | United Kingdom | Both | 15-39 years | Number | 2021 | 31730.73 | 44621.35 | 21473.87 |
| DALYs | United Republic of Tanzania | Both | 15-39 years | Number | 2021 | 41187.15 | 58799.32 | 28172.32 |
| DALYs | United States of America | Both | 15-39 years | Number | 2021 | 235612.30 | 329838.73 | 163458.12 |
| DALYs | United States Virgin Islands | Both | 15-39 years | Number | 2021 | 32.16 | 44.78 | 22.01 |
| DALYs | Uruguay | Both | 15-39 years | Number | 2021 | 2494.42 | 3563.89 | 1709.37 |
| DALYs | Uzbekistan | Both | 15-39 years | Number | 2021 | 23824.15 | 33997.44 | 16053.46 |
| DALYs | Vanuatu | Both | 15-39 years | Number | 2021 | 164.88 | 237.55 | 114.29 |
| DALYs | Venezuela (Bolivarian Republic of) | Both | 15-39 years | Number | 2021 | 13802.27 | 19645.92 | 9420.86 |
| DALYs | Viet Nam | Both | 15-39 years | Number | 2021 | 55260.02 | 77178.54 | 38514.22 |
| DALYs | Yemen | Both | 15-39 years | Number | 2021 | 19580.43 | 27974.54 | 13396.15 |
| DALYs | Zambia | Both | 15-39 years | Number | 2021 | 14230.50 | 19866.96 | 9745.67 |
| DALYs | Zimbabwe | Both | 15-39 years | Number | 2021 | 10966.02 | 15625.38 | 7504.86 |

**Table S3.** Number of prevalent ASD cases in adolescents and young adults by country, 2021.

| **Measure** | **Location** | **Sex** | **Age** | **Metric** | **Year** | **Val** | **Upper** | **Lower** |
| --- | --- | --- | --- | --- | --- | --- | --- | --- |
| Prevalence | Afghanistan | Both | 15-39 years | Number | 2021 | 91403.86 | 108900.75 | 75632.75 |
| Prevalence | Albania | Both | 15-39 years | Number | 2021 | 9336.87 | 11005.22 | 7733.22 |
| Prevalence | Algeria | Both | 15-39 years | Number | 2021 | 136615.68 | 162193.59 | 114196.53 |
| Prevalence | American Samoa | Both | 15-39 years | Number | 2021 | 128.99 | 153.85 | 107.14 |
| Prevalence | Andorra | Both | 15-39 years | Number | 2021 | 239.66 | 285.24 | 199.91 |
| Prevalence | Angola | Both | 15-39 years | Number | 2021 | 112652.28 | 132373.81 | 94515.14 |
| Prevalence | Antigua and Barbuda | Both | 15-39 years | Number | 2021 | 255.53 | 303.33 | 211.75 |
| Prevalence | Argentina | Both | 15-39 years | Number | 2021 | 191675.88 | 226635.33 | 159485.04 |
| Prevalence | Armenia | Both | 15-39 years | Number | 2021 | 10293.23 | 12113.83 | 8596.37 |
| Prevalence | Australia | Both | 15-39 years | Number | 2021 | 106494.83 | 127505.08 | 88902.64 |
| Prevalence | Austria | Both | 15-39 years | Number | 2021 | 26336.32 | 31671.88 | 21833.18 |
| Prevalence | Azerbaijan | Both | 15-39 years | Number | 2021 | 39427.61 | 46344.06 | 32971.33 |
| Prevalence | Bahamas | Both | 15-39 years | Number | 2021 | 1123.33 | 1334.40 | 929.11 |
| Prevalence | Bahrain | Both | 15-39 years | Number | 2021 | 6366.49 | 7557.51 | 5301.70 |
| Prevalence | Bangladesh | Both | 15-39 years | Number | 2021 | 419489.16 | 497090.87 | 347259.86 |
| Prevalence | Barbados | Both | 15-39 years | Number | 2021 | 734.55 | 883.83 | 616.83 |
| Prevalence | Belarus | Both | 15-39 years | Number | 2021 | 28982.43 | 34609.79 | 24073.89 |
| Prevalence | Belgium | Both | 15-39 years | Number | 2021 | 32252.27 | 38307.26 | 26870.86 |
| Prevalence | Belize | Both | 15-39 years | Number | 2021 | 1346.64 | 1618.52 | 1124.26 |
| Prevalence | Benin | Both | 15-39 years | Number | 2021 | 48875.68 | 58049.15 | 40637.55 |
| Prevalence | Bermuda | Both | 15-39 years | Number | 2021 | 133.76 | 161.31 | 111.08 |
| Prevalence | Bhutan | Both | 15-39 years | Number | 2021 | 2363.64 | 2808.86 | 1970.94 |
| Prevalence | Bolivia (Plurinational State of) | Both | 15-39 years | Number | 2021 | 34242.47 | 41038.91 | 28625.48 |
| Prevalence | Bosnia and Herzegovina | Both | 15-39 years | Number | 2021 | 10172.03 | 12036.27 | 8460.48 |
| Prevalence | Botswana | Both | 15-39 years | Number | 2021 | 10146.45 | 12051.51 | 8473.37 |
| Prevalence | Brazil | Both | 15-39 years | Number | 2021 | 547824.66 | 651757.41 | 458556.58 |
| Prevalence | Brunei Darussalam | Both | 15-39 years | Number | 2021 | 2946.79 | 3508.30 | 2482.02 |
| Prevalence | Bulgaria | Both | 15-39 years | Number | 2021 | 18544.55 | 22070.19 | 15480.18 |
| Prevalence | Burkina Faso | Both | 15-39 years | Number | 2021 | 79028.95 | 93335.03 | 66404.81 |
| Prevalence | Burundi | Both | 15-39 years | Number | 2021 | 48941.35 | 57793.28 | 40843.91 |
| Prevalence | Cabo Verde | Both | 15-39 years | Number | 2021 | 2507.03 | 2980.93 | 2086.76 |
| Prevalence | Cambodia | Both | 15-39 years | Number | 2021 | 51239.29 | 61166.56 | 42700.55 |
| Prevalence | Cameroon | Both | 15-39 years | Number | 2021 | 120576.29 | 143146.87 | 101288.74 |
| Prevalence | Canada | Both | 15-39 years | Number | 2021 | 137203.83 | 162149.48 | 114405.82 |
| Prevalence | Central African Republic | Both | 15-39 years | Number | 2021 | 19549.12 | 23679.45 | 16388.12 |
| Prevalence | Chad | Both | 15-39 years | Number | 2021 | 57569.73 | 69100.61 | 47883.84 |
| Prevalence | Chile | Both | 15-39 years | Number | 2021 | 80153.92 | 94403.13 | 67040.63 |
| Prevalence | China | Both | 15-39 years | Number | 2021 | 3123233.96 | 3717148.06 | 2602308.00 |
| Prevalence | Colombia | Both | 15-39 years | Number | 2021 | 162279.14 | 193167.82 | 135046.76 |
| Prevalence | Comoros | Both | 15-39 years | Number | 2021 | 2958.51 | 3508.58 | 2469.84 |
| Prevalence | Congo | Both | 15-39 years | Number | 2021 | 20750.94 | 24587.41 | 17361.70 |
| Prevalence | Cook Islands | Both | 15-39 years | Number | 2021 | 43.47 | 51.83 | 36.27 |
| Prevalence | Costa Rica | Both | 15-39 years | Number | 2021 | 15634.44 | 18700.98 | 12959.05 |
| Prevalence | Croatia | Both | 15-39 years | Number | 2021 | 12875.81 | 15338.54 | 10660.05 |
| Prevalence | Cuba | Both | 15-39 years | Number | 2021 | 27083.76 | 32188.19 | 22613.60 |
| Prevalence | Cyprus | Both | 15-39 years | Number | 2021 | 4619.82 | 5490.55 | 3844.30 |
| Prevalence | Czechia | Both | 15-39 years | Number | 2021 | 30470.96 | 35937.51 | 25288.21 |
| Prevalence | Côte d'Ivoire | Both | 15-39 years | Number | 2021 | 106214.05 | 127021.63 | 88396.27 |
| Prevalence | Democratic People's Republic of Korea | Both | 15-39 years | Number | 2021 | 74648.30 | 89161.49 | 62184.23 |
| Prevalence | Democratic Republic of the Congo | Both | 15-39 years | Number | 2021 | 339053.02 | 404520.14 | 282349.54 |
| Prevalence | Denmark | Both | 15-39 years | Number | 2021 | 18040.62 | 21445.82 | 15230.85 |
| Prevalence | Djibouti | Both | 15-39 years | Number | 2021 | 5270.61 | 6273.35 | 4395.47 |
| Prevalence | Dominica | Both | 15-39 years | Number | 2021 | 188.83 | 225.52 | 158.73 |
| Prevalence | Dominican Republic | Both | 15-39 years | Number | 2021 | 32260.98 | 38427.98 | 26905.56 |
| Prevalence | Ecuador | Both | 15-39 years | Number | 2021 | 52128.01 | 61951.15 | 43301.83 |
| Prevalence | Egypt | Both | 15-39 years | Number | 2021 | 334742.78 | 397323.20 | 280113.02 |
| Prevalence | El Salvador | Both | 15-39 years | Number | 2021 | 20231.73 | 24138.07 | 16894.19 |
| Prevalence | Equatorial Guinea | Both | 15-39 years | Number | 2021 | 6923.04 | 8177.52 | 5761.35 |
| Prevalence | Eritrea | Both | 15-39 years | Number | 2021 | 26476.99 | 31367.11 | 22056.92 |
| Prevalence | Estonia | Both | 15-39 years | Number | 2021 | 3982.35 | 4733.50 | 3353.07 |
| Prevalence | Eswatini | Both | 15-39 years | Number | 2021 | 4753.80 | 5672.81 | 3963.41 |
| Prevalence | Ethiopia | Both | 15-39 years | Number | 2021 | 434448.90 | 510096.25 | 365367.53 |
| Prevalence | Fiji | Both | 15-39 years | Number | 2021 | 2555.69 | 3050.71 | 2118.56 |
| Prevalence | Finland | Both | 15-39 years | Number | 2021 | 14476.82 | 17315.39 | 12045.85 |
| Prevalence | France | Both | 15-39 years | Number | 2021 | 182250.62 | 217391.80 | 152420.96 |
| Prevalence | Gabon | Both | 15-39 years | Number | 2021 | 7063.63 | 8423.37 | 5892.04 |
| Prevalence | Gambia | Both | 15-39 years | Number | 2021 | 9375.77 | 11092.79 | 7872.35 |
| Prevalence | Georgia | Both | 15-39 years | Number | 2021 | 10828.52 | 12852.55 | 9051.70 |
| Prevalence | Germany | Both | 15-39 years | Number | 2021 | 238819.93 | 284962.80 | 196988.27 |
| Prevalence | Ghana | Both | 15-39 years | Number | 2021 | 134180.08 | 159793.09 | 112415.32 |
| Prevalence | Greece | Both | 15-39 years | Number | 2021 | 25560.19 | 30681.82 | 21290.20 |
| Prevalence | Greenland | Both | 15-39 years | Number | 2021 | 215.23 | 253.87 | 180.08 |
| Prevalence | Grenada | Both | 15-39 years | Number | 2021 | 297.11 | 351.94 | 247.09 |
| Prevalence | Guam | Both | 15-39 years | Number | 2021 | 420.30 | 503.42 | 347.20 |
| Prevalence | Guatemala | Both | 15-39 years | Number | 2021 | 51768.67 | 61383.42 | 43137.09 |
| Prevalence | Guinea | Both | 15-39 years | Number | 2021 | 47009.42 | 55896.31 | 39313.22 |
| Prevalence | Guinea-Bissau | Both | 15-39 years | Number | 2021 | 7662.52 | 9190.07 | 6420.86 |
| Prevalence | Guyana | Both | 15-39 years | Number | 2021 | 2170.64 | 2582.20 | 1815.70 |
| Prevalence | Haiti | Both | 15-39 years | Number | 2021 | 36298.96 | 43185.58 | 30250.39 |
| Prevalence | Honduras | Both | 15-39 years | Number | 2021 | 33498.56 | 39753.75 | 27969.12 |
| Prevalence | Hungary | Both | 15-39 years | Number | 2021 | 27809.19 | 32965.49 | 23165.05 |
| Prevalence | Iceland | Both | 15-39 years | Number | 2021 | 1306.57 | 1541.95 | 1094.08 |
| Prevalence | India | Both | 15-39 years | Number | 2021 | 4530467.18 | 5305568.46 | 3807164.09 |
| Prevalence | Indonesia | Both | 15-39 years | Number | 2021 | 768666.59 | 913055.55 | 646410.09 |
| Prevalence | Iran (Islamic Republic of) | Both | 15-39 years | Number | 2021 | 283752.18 | 334750.67 | 237980.66 |
| Prevalence | Iraq | Both | 15-39 years | Number | 2021 | 129167.61 | 155202.07 | 108047.13 |
| Prevalence | Ireland | Both | 15-39 years | Number | 2021 | 17337.91 | 20625.27 | 14480.23 |
| Prevalence | Israel | Both | 15-39 years | Number | 2021 | 27621.18 | 32889.38 | 23112.50 |
| Prevalence | Italy | Both | 15-39 years | Number | 2021 | 164648.02 | 195704.15 | 137987.17 |
| Prevalence | Jamaica | Both | 15-39 years | Number | 2021 | 8752.88 | 10381.25 | 7358.53 |
| Prevalence | Japan | Both | 15-39 years | Number | 2021 | 532197.63 | 624689.21 | 448343.79 |
| Prevalence | Jordan | Both | 15-39 years | Number | 2021 | 45413.90 | 53854.83 | 37865.99 |
| Prevalence | Kazakhstan | Both | 15-39 years | Number | 2021 | 65354.94 | 77486.71 | 54913.62 |
| Prevalence | Kenya | Both | 15-39 years | Number | 2021 | 205670.95 | 240974.09 | 172779.65 |
| Prevalence | Kiribati | Both | 15-39 years | Number | 2021 | 338.52 | 405.24 | 282.32 |
| Prevalence | Kuwait | Both | 15-39 years | Number | 2021 | 17877.08 | 21241.56 | 14797.42 |
| Prevalence | Kyrgyzstan | Both | 15-39 years | Number | 2021 | 25347.40 | 30330.68 | 21289.65 |
| Prevalence | Lao People's Democratic Republic | Both | 15-39 years | Number | 2021 | 22422.79 | 26986.51 | 18737.96 |
| Prevalence | Latvia | Both | 15-39 years | Number | 2021 | 5316.36 | 6293.33 | 4387.51 |
| Prevalence | Lebanon | Both | 15-39 years | Number | 2021 | 21141.33 | 24982.00 | 17631.36 |
| Prevalence | Lesotho | Both | 15-39 years | Number | 2021 | 7715.70 | 9195.71 | 6462.61 |
| Prevalence | Liberia | Both | 15-39 years | Number | 2021 | 21161.15 | 25136.39 | 17643.66 |
| Prevalence | Libya | Both | 15-39 years | Number | 2021 | 24010.44 | 28503.63 | 19942.13 |
| Prevalence | Lithuania | Both | 15-39 years | Number | 2021 | 7975.06 | 9433.82 | 6627.85 |
| Prevalence | Luxembourg | Both | 15-39 years | Number | 2021 | 2064.20 | 2480.92 | 1716.47 |
| Prevalence | Madagascar | Both | 15-39 years | Number | 2021 | 109526.04 | 129893.88 | 91579.46 |
| Prevalence | Malawi | Both | 15-39 years | Number | 2021 | 76317.93 | 90308.51 | 64026.37 |
| Prevalence | Malaysia | Both | 15-39 years | Number | 2021 | 106323.37 | 127285.52 | 88577.29 |
| Prevalence | Maldives | Both | 15-39 years | Number | 2021 | 2155.53 | 2571.60 | 1773.83 |
| Prevalence | Mali | Both | 15-39 years | Number | 2021 | 82302.58 | 98051.79 | 68803.00 |
| Prevalence | Malta | Both | 15-39 years | Number | 2021 | 1252.80 | 1490.54 | 1048.97 |
| Prevalence | Marshall Islands | Both | 15-39 years | Number | 2021 | 168.59 | 201.24 | 140.50 |
| Prevalence | Mauritania | Both | 15-39 years | Number | 2021 | 16258.78 | 19234.51 | 13644.53 |
| Prevalence | Mauritius | Both | 15-39 years | Number | 2021 | 3448.12 | 4089.17 | 2852.87 |
| Prevalence | Mexico | Both | 15-39 years | Number | 2021 | 408120.34 | 485099.88 | 344951.53 |
| Prevalence | Micronesia (Federated States of) | Both | 15-39 years | Number | 2021 | 305.14 | 360.18 | 256.22 |
| Prevalence | Monaco | Both | 15-39 years | Number | 2021 | 86.65 | 103.69 | 72.49 |
| Prevalence | Mongolia | Both | 15-39 years | Number | 2021 | 11408.91 | 13644.79 | 9604.92 |
| Prevalence | Montenegro | Both | 15-39 years | Number | 2021 | 2086.54 | 2452.85 | 1743.94 |
| Prevalence | Morocco | Both | 15-39 years | Number | 2021 | 115707.76 | 137035.86 | 97127.28 |
| Prevalence | Mozambique | Both | 15-39 years | Number | 2021 | 110406.57 | 131903.31 | 92264.11 |
| Prevalence | Myanmar | Both | 15-39 years | Number | 2021 | 157234.24 | 188665.95 | 131042.99 |
| Prevalence | Namibia | Both | 15-39 years | Number | 2021 | 9966.59 | 11888.34 | 8341.02 |
| Prevalence | Nauru | Both | 15-39 years | Number | 2021 | 32.87 | 39.30 | 27.38 |
| Prevalence | Nepal | Both | 15-39 years | Number | 2021 | 87236.51 | 104964.02 | 72575.13 |
| Prevalence | Netherlands | Both | 15-39 years | Number | 2021 | 49421.99 | 59030.46 | 40654.35 |
| Prevalence | New Zealand | Both | 15-39 years | Number | 2021 | 21833.31 | 26232.39 | 18075.53 |
| Prevalence | Nicaragua | Both | 15-39 years | Number | 2021 | 22561.24 | 26634.00 | 18698.17 |
| Prevalence | Niger | Both | 15-39 years | Number | 2021 | 82513.48 | 98266.29 | 68768.99 |
| Prevalence | Nigeria | Both | 15-39 years | Number | 2021 | 829254.54 | 974779.18 | 699152.84 |
| Prevalence | Niue | Both | 15-39 years | Number | 2021 | 4.16 | 4.89 | 3.48 |
| Prevalence | North Macedonia | Both | 15-39 years | Number | 2021 | 7603.93 | 9034.68 | 6368.17 |
| Prevalence | Northern Mariana Islands | Both | 15-39 years | Number | 2021 | 125.23 | 148.82 | 104.43 |
| Prevalence | Norway | Both | 15-39 years | Number | 2021 | 14939.18 | 17820.67 | 12556.40 |
| Prevalence | Oman | Both | 15-39 years | Number | 2021 | 20709.95 | 24848.67 | 17147.40 |
| Prevalence | Pakistan | Both | 15-39 years | Number | 2021 | 654270.16 | 772356.48 | 546512.30 |
| Prevalence | Palau | Both | 15-39 years | Number | 2021 | 45.65 | 53.76 | 37.92 |
| Prevalence | Palestine | Both | 15-39 years | Number | 2021 | 17813.23 | 21258.86 | 14838.36 |
| Prevalence | Panama | Both | 15-39 years | Number | 2021 | 13407.38 | 15894.51 | 11243.94 |
| Prevalence | Papua New Guinea | Both | 15-39 years | Number | 2021 | 29826.30 | 35705.57 | 25041.90 |
| Prevalence | Paraguay | Both | 15-39 years | Number | 2021 | 20134.59 | 24028.94 | 16725.74 |
| Prevalence | Peru | Both | 15-39 years | Number | 2021 | 107058.25 | 127757.33 | 89539.18 |
| Prevalence | Philippines | Both | 15-39 years | Number | 2021 | 338829.38 | 400298.11 | 284593.33 |
| Prevalence | Poland | Both | 15-39 years | Number | 2021 | 121163.90 | 143394.30 | 101410.87 |
| Prevalence | Portugal | Both | 15-39 years | Number | 2021 | 26568.48 | 31490.45 | 22194.51 |
| Prevalence | Puerto Rico | Both | 15-39 years | Number | 2021 | 7862.68 | 9415.31 | 6473.19 |
| Prevalence | Qatar | Both | 15-39 years | Number | 2021 | 17347.22 | 20588.89 | 14371.07 |
| Prevalence | Republic of Korea | Both | 15-39 years | Number | 2021 | 251263.70 | 296781.63 | 210176.31 |
| Prevalence | Republic of Moldova | Both | 15-39 years | Number | 2021 | 11904.52 | 14077.24 | 9967.38 |
| Prevalence | Romania | Both | 15-39 years | Number | 2021 | 52661.67 | 62100.43 | 44246.56 |
| Prevalence | Russian Federation | Both | 15-39 years | Number | 2021 | 450954.02 | 532608.35 | 378423.55 |
| Prevalence | Rwanda | Both | 15-39 years | Number | 2021 | 53436.78 | 63384.83 | 44812.19 |
| Prevalence | Saint Kitts and Nevis | Both | 15-39 years | Number | 2021 | 165.02 | 195.13 | 138.33 |
| Prevalence | Saint Lucia | Both | 15-39 years | Number | 2021 | 486.26 | 580.06 | 403.57 |
| Prevalence | Saint Vincent and the Grenadines | Both | 15-39 years | Number | 2021 | 299.92 | 356.27 | 249.31 |
| Prevalence | Samoa | Both | 15-39 years | Number | 2021 | 583.18 | 693.21 | 486.21 |
| Prevalence | San Marino | Both | 15-39 years | Number | 2021 | 82.83 | 99.07 | 68.98 |
| Prevalence | Sao Tome and Principe | Both | 15-39 years | Number | 2021 | 878.45 | 1035.12 | 731.92 |
| Prevalence | Saudi Arabia | Both | 15-39 years | Number | 2021 | 158203.87 | 188058.05 | 130839.79 |
| Prevalence | Senegal | Both | 15-39 years | Number | 2021 | 61044.91 | 72343.99 | 50991.13 |
| Prevalence | Serbia | Both | 15-39 years | Number | 2021 | 29900.78 | 35673.85 | 24814.71 |
| Prevalence | Seychelles | Both | 15-39 years | Number | 2021 | 296.76 | 353.24 | 245.88 |
| Prevalence | Sierra Leone | Both | 15-39 years | Number | 2021 | 34614.68 | 41295.12 | 28893.43 |
| Prevalence | Singapore | Both | 15-39 years | Number | 2021 | 29335.26 | 34762.32 | 24783.83 |
| Prevalence | Slovakia | Both | 15-39 years | Number | 2021 | 17188.82 | 20475.84 | 14448.22 |
| Prevalence | Slovenia | Both | 15-39 years | Number | 2021 | 5987.93 | 7182.43 | 4978.78 |
| Prevalence | Solomon Islands | Both | 15-39 years | Number | 2021 | 1919.04 | 2284.19 | 1610.66 |
| Prevalence | Somalia | Both | 15-39 years | Number | 2021 | 77128.30 | 90769.90 | 64772.63 |
| Prevalence | South Africa | Both | 15-39 years | Number | 2021 | 232692.75 | 276426.47 | 195630.48 |
| Prevalence | South Sudan | Both | 15-39 years | Number | 2021 | 33481.23 | 39876.76 | 28088.33 |
| Prevalence | Spain | Both | 15-39 years | Number | 2021 | 132001.79 | 154890.39 | 111536.88 |
| Prevalence | Sri Lanka | Both | 15-39 years | Number | 2021 | 62522.65 | 74595.55 | 51602.87 |
| Prevalence | Sudan | Both | 15-39 years | Number | 2021 | 141783.95 | 169155.40 | 117967.89 |
| Prevalence | Suriname | Both | 15-39 years | Number | 2021 | 1516.19 | 1804.43 | 1265.54 |
| Prevalence | Sweden | Both | 15-39 years | Number | 2021 | 34500.92 | 41190.51 | 28570.00 |
| Prevalence | Switzerland | Both | 15-39 years | Number | 2021 | 26049.73 | 31106.75 | 21630.83 |
| Prevalence | Syrian Arab Republic | Both | 15-39 years | Number | 2021 | 39361.91 | 47235.05 | 32619.35 |
| Prevalence | Taiwan (Province of China) | Both | 15-39 years | Number | 2021 | 70668.32 | 83998.26 | 58252.77 |
| Prevalence | Tajikistan | Both | 15-39 years | Number | 2021 | 37821.63 | 44531.92 | 31682.96 |
| Prevalence | Thailand | Both | 15-39 years | Number | 2021 | 157563.72 | 188319.74 | 130989.84 |
| Prevalence | Timor-Leste | Both | 15-39 years | Number | 2021 | 4022.33 | 4777.03 | 3352.11 |
| Prevalence | Togo | Both | 15-39 years | Number | 2021 | 31376.79 | 37433.13 | 26387.40 |
| Prevalence | Tokelau | Both | 15-39 years | Number | 2021 | 3.69 | 4.42 | 3.10 |
| Prevalence | Tonga | Both | 15-39 years | Number | 2021 | 280.52 | 335.14 | 233.03 |
| Prevalence | Trinidad and Tobago | Both | 15-39 years | Number | 2021 | 3634.62 | 4335.98 | 3057.66 |
| Prevalence | Tunisia | Both | 15-39 years | Number | 2021 | 35236.14 | 41703.03 | 29213.10 |
| Prevalence | Turkmenistan | Both | 15-39 years | Number | 2021 | 19460.00 | 22917.00 | 16299.50 |
| Prevalence | Tuvalu | Both | 15-39 years | Number | 2021 | 35.83 | 42.85 | 29.68 |
| Prevalence | Turkey | Both | 15-39 years | Number | 2021 | 260450.73 | 307546.42 | 216300.16 |
| Prevalence | Uganda | Both | 15-39 years | Number | 2021 | 163202.64 | 193410.01 | 136627.29 |
| Prevalence | Ukraine | Both | 15-39 years | Number | 2021 | 133191.10 | 158816.65 | 111442.33 |
| Prevalence | United Arab Emirates | Both | 15-39 years | Number | 2021 | 36452.97 | 43354.15 | 30529.57 |
| Prevalence | United Kingdom | Both | 15-39 years | Number | 2021 | 169429.54 | 200200.25 | 142433.36 |
| Prevalence | United Republic of Tanzania | Both | 15-39 years | Number | 2021 | 218996.13 | 259417.71 | 183035.77 |
| Prevalence | United States of America | Both | 15-39 years | Number | 2021 | 1267373.43 | 1489566.22 | 1069095.92 |
| Prevalence | United States Virgin Islands | Both | 15-39 years | Number | 2021 | 170.44 | 203.83 | 141.37 |
| Prevalence | Uruguay | Both | 15-39 years | Number | 2021 | 13234.20 | 15865.15 | 11012.35 |
| Prevalence | Uzbekistan | Both | 15-39 years | Number | 2021 | 126253.20 | 150126.34 | 105213.37 |
| Prevalence | Vanuatu | Both | 15-39 years | Number | 2021 | 872.41 | 1045.46 | 722.08 |
| Prevalence | Venezuela (Bolivarian Republic of) | Both | 15-39 years | Number | 2021 | 73167.15 | 86787.96 | 61394.67 |
| Prevalence | Viet Nam | Both | 15-39 years | Number | 2021 | 290204.24 | 346527.18 | 243038.80 |
| Prevalence | Yemen | Both | 15-39 years | Number | 2021 | 104696.53 | 124605.52 | 88015.93 |
| Prevalence | Zambia | Both | 15-39 years | Number | 2021 | 75917.42 | 90093.07 | 63206.09 |
| Prevalence | Zimbabwe | Both | 15-39 years | Number | 2021 | 58437.82 | 69729.41 | 48891.32 |

**Table S4.** Age-standardised DALY rates for autism spectrum disorder among adolescents and young adults (15–39 years), by country, 2021

| **Measure** | **Location** | **Sex** | **Age** | **Metric** | **Year** | **Val** | **Lower** | **Upper** |
| --- | --- | --- | --- | --- | --- | --- | --- | --- |
| DALYs | Afghanistan | Both | Age-standardized | Rate | 2021 | 138.54 | 94.18 | 198.63 |
| DALYs | Albania | Both | Age-standardized | Rate | 2021 | 186.30 | 125.32 | 261.19 |
| DALYs | Algeria | Both | Age-standardized | Rate | 2021 | 151.76 | 101.98 | 214.02 |
| DALYs | American Samoa | Both | Age-standardized | Rate | 2021 | 139.19 | 94.90 | 198.24 |
| DALYs | Andorra | Both | Age-standardized | Rate | 2021 | 177.34 | 118.93 | 249.17 |
| DALYs | Angola | Both | Age-standardized | Rate | 2021 | 172.06 | 117.25 | 240.90 |
| DALYs | Antigua and Barbuda | Both | Age-standardized | Rate | 2021 | 140.16 | 94.51 | 199.12 |
| DALYs | Argentina | Both | Age-standardized | Rate | 2021 | 206.23 | 139.26 | 290.00 |
| DALYs | Armenia | Both | Age-standardized | Rate | 2021 | 181.97 | 124.49 | 259.26 |
| DALYs | Australia | Both | Age-standardized | Rate | 2021 | 230.62 | 158.82 | 325.37 |
| DALYs | Austria | Both | Age-standardized | Rate | 2021 | 176.10 | 118.20 | 247.34 |
| DALYs | Azerbaijan | Both | Age-standardized | Rate | 2021 | 176.71 | 119.58 | 247.21 |
| DALYs | Bahamas | Both | Age-standardized | Rate | 2021 | 136.90 | 92.85 | 193.91 |
| DALYs | Bahrain | Both | Age-standardized | Rate | 2021 | 169.89 | 114.21 | 239.33 |
| DALYs | Bangladesh | Both | Age-standardized | Rate | 2021 | 115.01 | 77.64 | 163.76 |
| DALYs | Barbados | Both | Age-standardized | Rate | 2021 | 140.48 | 95.05 | 199.40 |
| DALYs | Belarus | Both | Age-standardized | Rate | 2021 | 187.17 | 126.56 | 262.60 |
| DALYs | Belgium | Both | Age-standardized | Rate | 2021 | 173.72 | 116.69 | 243.65 |
| DALYs | Belize | Both | Age-standardized | Rate | 2021 | 133.96 | 90.53 | 189.39 |
| DALYs | Benin | Both | Age-standardized | Rate | 2021 | 175.15 | 116.71 | 248.02 |
| DALYs | Bermuda | Both | Age-standardized | Rate | 2021 | 144.88 | 96.43 | 204.45 |
| DALYs | Bhutan | Both | Age-standardized | Rate | 2021 | 128.82 | 87.66 | 182.47 |
| DALYs | Bolivia (Plurinational State of) | Both | Age-standardized | Rate | 2021 | 130.94 | 89.14 | 184.50 |
| DALYs | Bosnia and Herzegovina | Both | Age-standardized | Rate | 2021 | 191.32 | 131.27 | 271.57 |
| DALYs | Botswana | Both | Age-standardized | Rate | 2021 | 178.06 | 122.01 | 249.65 |
| DALYs | Brazil | Both | Age-standardized | Rate | 2021 | 120.45 | 81.44 | 169.41 |
| DALYs | Brunei Darussalam | Both | Age-standardized | Rate | 2021 | 274.28 | 186.43 | 383.61 |
| DALYs | Bulgaria | Both | Age-standardized | Rate | 2021 | 184.98 | 125.70 | 262.50 |
| DALYs | Burkina Faso | Both | Age-standardized | Rate | 2021 | 171.43 | 115.33 | 241.44 |
| DALYs | Burundi | Both | Age-standardized | Rate | 2021 | 173.98 | 117.75 | 247.53 |
| DALYs | Cabo Verde | Both | Age-standardized | Rate | 2021 | 188.76 | 127.73 | 266.22 |
| DALYs | Cambodia | Both | Age-standardized | Rate | 2021 | 134.33 | 89.92 | 190.87 |
| DALYs | Cameroon | Both | Age-standardized | Rate | 2021 | 175.30 | 119.95 | 248.30 |
| DALYs | Canada | Both | Age-standardized | Rate | 2021 | 217.97 | 149.47 | 309.40 |
| DALYs | Central African Republic | Both | Age-standardized | Rate | 2021 | 165.94 | 113.10 | 236.84 |
| DALYs | Chad | Both | Age-standardized | Rate | 2021 | 170.48 | 116.10 | 242.03 |
| DALYs | Chile | Both | Age-standardized | Rate | 2021 | 213.65 | 145.28 | 301.82 |
| DALYs | China | Both | Age-standardized | Rate | 2021 | 129.69 | 88.19 | 183.74 |
| DALYs | Colombia | Both | Age-standardized | Rate | 2021 | 152.55 | 102.79 | 217.58 |
| DALYs | Comoros | Both | Age-standardized | Rate | 2021 | 180.14 | 123.35 | 256.98 |
| DALYs | Congo | Both | Age-standardized | Rate | 2021 | 174.93 | 119.02 | 247.03 |
| DALYs | Cook Islands | Both | Age-standardized | Rate | 2021 | 139.59 | 94.81 | 198.42 |
| DALYs | Costa Rica | Both | Age-standardized | Rate | 2021 | 155.27 | 104.03 | 221.59 |
| DALYs | Croatia | Both | Age-standardized | Rate | 2021 | 195.58 | 133.02 | 279.62 |
| DALYs | Cuba | Both | Age-standardized | Rate | 2021 | 142.74 | 97.43 | 199.15 |
| DALYs | Cyprus | Both | Age-standardized | Rate | 2021 | 174.32 | 116.46 | 246.58 |
| DALYs | Czechia | Both | Age-standardized | Rate | 2021 | 195.59 | 134.22 | 276.85 |
| DALYs | Côte d'Ivoire | Both | Age-standardized | Rate | 2021 | 177.80 | 119.48 | 251.56 |
| DALYs | Democratic People's Republic of Korea | Both | Age-standardized | Rate | 2021 | 141.93 | 96.32 | 201.28 |
| DALYs | Democratic Republic of the Congo | Both | Age-standardized | Rate | 2021 | 174.69 | 118.82 | 251.70 |
| DALYs | Denmark | Both | Age-standardized | Rate | 2021 | 186.14 | 126.36 | 261.10 |
| DALYs | Djibouti | Both | Age-standardized | Rate | 2021 | 184.07 | 126.22 | 262.52 |
| DALYs | Dominica | Both | Age-standardized | Rate | 2021 | 137.52 | 94.26 | 197.30 |
| DALYs | Dominican Republic | Both | Age-standardized | Rate | 2021 | 133.74 | 90.60 | 187.13 |
| DALYs | Ecuador | Both | Age-standardized | Rate | 2021 | 134.69 | 90.74 | 191.50 |
| DALYs | Egypt | Both | Age-standardized | Rate | 2021 | 149.28 | 100.16 | 213.51 |
| DALYs | El Salvador | Both | Age-standardized | Rate | 2021 | 146.53 | 99.08 | 207.94 |
| DALYs | Equatorial Guinea | Both | Age-standardized | Rate | 2021 | 185.65 | 125.20 | 265.99 |
| DALYs | Eritrea | Both | Age-standardized | Rate | 2021 | 177.71 | 121.46 | 250.14 |
| DALYs | Estonia | Both | Age-standardized | Rate | 2021 | 190.30 | 128.99 | 266.91 |
| DALYs | Eswatini | Both | Age-standardized | Rate | 2021 | 173.23 | 117.67 | 242.57 |
| DALYs | Ethiopia | Both | Age-standardized | Rate | 2021 | 175.14 | 121.88 | 244.78 |
| DALYs | Fiji | Both | Age-standardized | Rate | 2021 | 135.40 | 91.94 | 191.49 |
| DALYs | Finland | Both | Age-standardized | Rate | 2021 | 163.49 | 110.50 | 234.41 |
| DALYs | France | Both | Age-standardized | Rate | 2021 | 172.80 | 116.90 | 244.56 |
| DALYs | Gabon | Both | Age-standardized | Rate | 2021 | 175.80 | 118.02 | 249.42 |
| DALYs | Gambia | Both | Age-standardized | Rate | 2021 | 175.38 | 121.04 | 247.77 |
| DALYs | Georgia | Both | Age-standardized | Rate | 2021 | 181.01 | 124.24 | 257.27 |
| DALYs | Germany | Both | Age-standardized | Rate | 2021 | 177.77 | 120.81 | 249.29 |
| DALYs | Ghana | Both | Age-standardized | Rate | 2021 | 176.30 | 118.54 | 247.00 |
| DALYs | Greece | Both | Age-standardized | Rate | 2021 | 172.68 | 116.69 | 245.24 |
| DALYs | Greenland | Both | Age-standardized | Rate | 2021 | 197.45 | 133.55 | 278.48 |
| DALYs | Grenada | Both | Age-standardized | Rate | 2021 | 138.40 | 92.73 | 196.11 |
| DALYs | Guam | Both | Age-standardized | Rate | 2021 | 143.70 | 97.83 | 203.89 |
| DALYs | Guatemala | Both | Age-standardized | Rate | 2021 | 142.08 | 96.10 | 201.01 |
| DALYs | Guinea | Both | Age-standardized | Rate | 2021 | 169.82 | 115.69 | 239.69 |
| DALYs | Guinea-Bissau | Both | Age-standardized | Rate | 2021 | 170.31 | 116.84 | 239.16 |
| DALYs | Guyana | Both | Age-standardized | Rate | 2021 | 130.22 | 87.84 | 183.48 |
| DALYs | Haiti | Both | Age-standardized | Rate | 2021 | 123.78 | 83.91 | 175.42 |
| DALYs | Honduras | Both | Age-standardized | Rate | 2021 | 143.03 | 95.87 | 203.44 |
| DALYs | Hungary | Both | Age-standardized | Rate | 2021 | 191.89 | 128.81 | 267.83 |
| DALYs | Iceland | Both | Age-standardized | Rate | 2021 | 205.93 | 140.92 | 287.32 |
| DALYs | India | Both | Age-standardized | Rate | 2021 | 139.68 | 95.37 | 196.71 |
| DALYs | Indonesia | Both | Age-standardized | Rate | 2021 | 128.29 | 86.73 | 181.43 |
| DALYs | Iran (Islamic Republic of) | Both | Age-standardized | Rate | 2021 | 153.90 | 104.85 | 216.65 |
| DALYs | Iraq | Both | Age-standardized | Rate | 2021 | 138.85 | 93.96 | 198.75 |
| DALYs | Ireland | Both | Age-standardized | Rate | 2021 | 208.22 | 140.99 | 288.60 |
| DALYs | Israel | Both | Age-standardized | Rate | 2021 | 157.09 | 106.63 | 220.99 |
| DALYs | Italy | Both | Age-standardized | Rate | 2021 | 196.85 | 134.87 | 275.84 |
| DALYs | Jamaica | Both | Age-standardized | Rate | 2021 | 138.34 | 94.32 | 195.55 |
| DALYs | Japan | Both | Age-standardized | Rate | 2021 | 311.87 | 215.62 | 436.99 |
| DALYs | Jordan | Both | Age-standardized | Rate | 2021 | 159.74 | 106.80 | 224.60 |
| DALYs | Kazakhstan | Both | Age-standardized | Rate | 2021 | 177.57 | 119.99 | 253.27 |
| DALYs | Kenya | Both | Age-standardized | Rate | 2021 | 178.13 | 122.28 | 249.79 |
| DALYs | Kiribati | Both | Age-standardized | Rate | 2021 | 128.53 | 86.50 | 182.50 |
| DALYs | Kuwait | Both | Age-standardized | Rate | 2021 | 159.46 | 107.23 | 224.08 |
| DALYs | Kyrgyzstan | Both | Age-standardized | Rate | 2021 | 176.16 | 118.20 | 249.05 |
| DALYs | Lao People's Democratic Republic | Both | Age-standardized | Rate | 2021 | 132.57 | 88.24 | 189.43 |
| DALYs | Latvia | Both | Age-standardized | Rate | 2021 | 186.79 | 127.00 | 263.89 |
| DALYs | Lebanon | Both | Age-standardized | Rate | 2021 | 171.28 | 116.00 | 241.08 |
| DALYs | Lesotho | Both | Age-standardized | Rate | 2021 | 171.56 | 116.56 | 240.84 |
| DALYs | Liberia | Both | Age-standardized | Rate | 2021 | 175.00 | 118.88 | 244.89 |
| DALYs | Libya | Both | Age-standardized | Rate | 2021 | 150.46 | 100.70 | 212.91 |
| DALYs | Lithuania | Both | Age-standardized | Rate | 2021 | 187.21 | 126.70 | 262.98 |
| DALYs | Luxembourg | Both | Age-standardized | Rate | 2021 | 176.82 | 120.47 | 249.81 |
| DALYs | Madagascar | Both | Age-standardized | Rate | 2021 | 175.10 | 120.43 | 247.15 |
| DALYs | Malawi | Both | Age-standardized | Rate | 2021 | 174.35 | 118.54 | 246.40 |
| DALYs | Malaysia | Both | Age-standardized | Rate | 2021 | 145.14 | 97.29 | 203.55 |
| DALYs | Maldives | Both | Age-standardized | Rate | 2021 | 155.40 | 102.50 | 220.42 |
| DALYs | Mali | Both | Age-standardized | Rate | 2021 | 172.48 | 118.00 | 244.95 |
| DALYs | Malta | Both | Age-standardized | Rate | 2021 | 176.69 | 118.76 | 249.60 |
| DALYs | Marshall Islands | Both | Age-standardized | Rate | 2021 | 134.22 | 91.07 | 191.58 |
| DALYs | Mauritania | Both | Age-standardized | Rate | 2021 | 179.45 | 120.79 | 253.23 |
| DALYs | Mauritius | Both | Age-standardized | Rate | 2021 | 142.84 | 96.45 | 203.21 |
| DALYs | Mexico | Both | Age-standardized | Rate | 2021 | 149.41 | 101.19 | 210.82 |
| DALYs | Micronesia (Federated States of) | Both | Age-standardized | Rate | 2021 | 135.60 | 90.53 | 192.51 |
| DALYs | Monaco | Both | Age-standardized | Rate | 2021 | 175.33 | 117.08 | 247.20 |
| DALYs | Mongolia | Both | Age-standardized | Rate | 2021 | 170.80 | 115.55 | 240.34 |
| DALYs | Montenegro | Both | Age-standardized | Rate | 2021 | 192.23 | 129.65 | 270.08 |
| DALYs | Morocco | Both | Age-standardized | Rate | 2021 | 147.82 | 98.38 | 208.19 |
| DALYs | Mozambique | Both | Age-standardized | Rate | 2021 | 169.75 | 116.68 | 241.94 |
| DALYs | Myanmar | Both | Age-standardized | Rate | 2021 | 132.67 | 88.41 | 188.96 |
| DALYs | Namibia | Both | Age-standardized | Rate | 2021 | 178.63 | 120.47 | 252.93 |
| DALYs | Nauru | Both | Age-standardized | Rate | 2021 | 133.32 | 89.67 | 189.25 |
| DALYs | Nepal | Both | Age-standardized | Rate | 2021 | 121.58 | 82.79 | 172.02 |
| DALYs | Netherlands | Both | Age-standardized | Rate | 2021 | 176.61 | 121.11 | 249.77 |
| DALYs | New Zealand | Both | Age-standardized | Rate | 2021 | 226.63 | 155.97 | 318.09 |
| DALYs | Nicaragua | Both | Age-standardized | Rate | 2021 | 149.49 | 100.47 | 210.34 |
| DALYs | Niger | Both | Age-standardized | Rate | 2021 | 173.98 | 117.79 | 249.33 |
| DALYs | Nigeria | Both | Age-standardized | Rate | 2021 | 172.62 | 119.28 | 243.14 |
| DALYs | Niue | Both | Age-standardized | Rate | 2021 | 138.18 | 93.15 | 195.47 |
| DALYs | North Macedonia | Both | Age-standardized | Rate | 2021 | 188.19 | 128.82 | 267.11 |
| DALYs | Northern Mariana Islands | Both | Age-standardized | Rate | 2021 | 144.02 | 96.71 | 202.24 |
| DALYs | Norway | Both | Age-standardized | Rate | 2021 | 158.75 | 109.06 | 223.50 |
| DALYs | Oman | Both | Age-standardized | Rate | 2021 | 166.76 | 112.57 | 234.47 |
| DALYs | Pakistan | Both | Age-standardized | Rate | 2021 | 124.01 | 84.90 | 173.88 |
| DALYs | Palau | Both | Age-standardized | Rate | 2021 | 146.59 | 96.98 | 208.81 |
| DALYs | Palestine | Both | Age-standardized | Rate | 2021 | 152.52 | 102.87 | 214.22 |
| DALYs | Panama | Both | Age-standardized | Rate | 2021 | 153.40 | 103.85 | 216.42 |
| DALYs | Papua New Guinea | Both | Age-standardized | Rate | 2021 | 131.06 | 88.93 | 186.63 |
| DALYs | Paraguay | Both | Age-standardized | Rate | 2021 | 123.53 | 83.23 | 172.32 |
| DALYs | Peru | Both | Age-standardized | Rate | 2021 | 136.25 | 91.52 | 192.33 |
| DALYs | Philippines | Both | Age-standardized | Rate | 2021 | 135.69 | 92.12 | 190.72 |
| DALYs | Poland | Both | Age-standardized | Rate | 2021 | 190.10 | 131.16 | 265.39 |
| DALYs | Portugal | Both | Age-standardized | Rate | 2021 | 169.22 | 115.11 | 238.81 |
| DALYs | Puerto Rico | Both | Age-standardized | Rate | 2021 | 143.53 | 97.17 | 202.46 |
| DALYs | Qatar | Both | Age-standardized | Rate | 2021 | 194.76 | 131.66 | 274.90 |
| DALYs | Republic of Korea | Both | Age-standardized | Rate | 2021 | 297.78 | 203.07 | 415.54 |
| DALYs | Republic of Moldova | Both | Age-standardized | Rate | 2021 | 182.34 | 123.82 | 258.25 |
| DALYs | Romania | Both | Age-standardized | Rate | 2021 | 185.19 | 125.65 | 259.57 |
| DALYs | Russian Federation | Both | Age-standardized | Rate | 2021 | 183.61 | 126.38 | 258.43 |
| DALYs | Rwanda | Both | Age-standardized | Rate | 2021 | 176.49 | 119.95 | 248.49 |
| DALYs | Saint Kitts and Nevis | Both | Age-standardized | Rate | 2021 | 137.13 | 92.73 | 196.37 |
| DALYs | Saint Lucia | Both | Age-standardized | Rate | 2021 | 138.60 | 92.26 | 198.02 |
| DALYs | Saint Vincent and the Grenadines | Both | Age-standardized | Rate | 2021 | 136.81 | 92.58 | 193.90 |
| DALYs | Samoa | Both | Age-standardized | Rate | 2021 | 137.25 | 92.48 | 193.86 |
| DALYs | San Marino | Both | Age-standardized | Rate | 2021 | 174.43 | 118.02 | 245.44 |
| DALYs | Sao Tome and Principe | Both | Age-standardized | Rate | 2021 | 182.53 | 124.19 | 260.71 |
| DALYs | Saudi Arabia | Both | Age-standardized | Rate | 2021 | 160.73 | 107.79 | 229.05 |
| DALYs | Senegal | Both | Age-standardized | Rate | 2021 | 177.23 | 120.95 | 249.82 |
| DALYs | Serbia | Both | Age-standardized | Rate | 2021 | 191.42 | 128.93 | 266.92 |
| DALYs | Seychelles | Both | Age-standardized | Rate | 2021 | 146.66 | 98.46 | 208.08 |
| DALYs | Sierra Leone | Both | Age-standardized | Rate | 2021 | 174.10 | 118.17 | 247.26 |
| DALYs | Singapore | Both | Age-standardized | Rate | 2021 | 290.95 | 197.82 | 407.24 |
| DALYs | Slovakia | Both | Age-standardized | Rate | 2021 | 190.31 | 129.58 | 268.14 |
| DALYs | Slovenia | Both | Age-standardized | Rate | 2021 | 198.94 | 134.79 | 281.81 |
| DALYs | Solomon Islands | Both | Age-standardized | Rate | 2021 | 132.22 | 89.20 | 186.88 |
| DALYs | Somalia | Both | Age-standardized | Rate | 2021 | 173.41 | 117.68 | 245.83 |
| DALYs | South Africa | Both | Age-standardized | Rate | 2021 | 179.20 | 122.54 | 250.77 |
| DALYs | South Sudan | Both | Age-standardized | Rate | 2021 | 172.12 | 117.59 | 242.97 |
| DALYs | Spain | Both | Age-standardized | Rate | 2021 | 200.98 | 137.92 | 278.66 |
| DALYs | Sri Lanka | Both | Age-standardized | Rate | 2021 | 146.88 | 100.05 | 207.95 |
| DALYs | Sudan | Both | Age-standardized | Rate | 2021 | 143.26 | 96.33 | 203.89 |
| DALYs | Suriname | Both | Age-standardized | Rate | 2021 | 132.12 | 89.08 | 187.26 |
| DALYs | Sweden | Both | Age-standardized | Rate | 2021 | 201.08 | 137.47 | 282.53 |
| DALYs | Switzerland | Both | Age-standardized | Rate | 2021 | 176.75 | 122.12 | 250.22 |
| DALYs | Syrian Arab Republic | Both | Age-standardized | Rate | 2021 | 141.51 | 94.88 | 200.24 |
| DALYs | Taiwan (Province of China) | Both | Age-standardized | Rate | 2021 | 178.84 | 120.50 | 253.97 |
| DALYs | Tajikistan | Both | Age-standardized | Rate | 2021 | 171.77 | 117.37 | 243.58 |
| DALYs | Thailand | Both | Age-standardized | Rate | 2021 | 141.24 | 96.70 | 198.96 |
| DALYs | Timor-Leste | Both | Age-standardized | Rate | 2021 | 132.37 | 89.91 | 188.90 |
| DALYs | Togo | Both | Age-standardized | Rate | 2021 | 175.38 | 118.16 | 245.61 |
| DALYs | Tokelau | Both | Age-standardized | Rate | 2021 | 140.86 | 94.44 | 201.96 |
| DALYs | Tonga | Both | Age-standardized | Rate | 2021 | 136.18 | 92.55 | 194.01 |
| DALYs | Trinidad and Tobago | Both | Age-standardized | Rate | 2021 | 137.78 | 93.01 | 195.85 |
| DALYs | Tunisia | Both | Age-standardized | Rate | 2021 | 152.32 | 101.35 | 215.38 |
| DALYs | Turkmenistan | Both | Age-standardized | Rate | 2021 | 177.05 | 121.43 | 250.86 |
| DALYs | Tuvalu | Both | Age-standardized | Rate | 2021 | 136.90 | 91.93 | 194.25 |
| DALYs | Turkey | Both | Age-standardized | Rate | 2021 | 154.38 | 104.94 | 217.69 |
| DALYs | Uganda | Both | Age-standardized | Rate | 2021 | 176.81 | 120.76 | 248.77 |
| DALYs | Ukraine | Both | Age-standardized | Rate | 2021 | 182.65 | 123.01 | 257.04 |
| DALYs | United Arab Emirates | Both | Age-standardized | Rate | 2021 | 163.73 | 109.64 | 231.46 |
| DALYs | United Kingdom | Both | Age-standardized | Rate | 2021 | 146.38 | 99.36 | 205.64 |
| DALYs | United Republic of Tanzania | Both | Age-standardized | Rate | 2021 | 175.59 | 118.75 | 247.24 |
| DALYs | United States of America | Both | Age-standardized | Rate | 2021 | 212.04 | 146.73 | 296.71 |
| DALYs | United States Virgin Islands | Both | Age-standardized | Rate | 2021 | 139.38 | 94.49 | 197.11 |
| DALYs | Uruguay | Both | Age-standardized | Rate | 2021 | 208.73 | 140.71 | 298.18 |
| DALYs | Uzbekistan | Both | Age-standardized | Rate | 2021 | 173.82 | 116.04 | 246.64 |
| DALYs | Vanuatu | Both | Age-standardized | Rate | 2021 | 131.87 | 89.18 | 188.68 |
| DALYs | Venezuela (Bolivarian Republic of) | Both | Age-standardized | Rate | 2021 | 147.61 | 99.83 | 208.98 |
| DALYs | Viet Nam | Both | Age-standardized | Rate | 2021 | 144.34 | 98.97 | 202.61 |
| DALYs | Yemen | Both | Age-standardized | Rate | 2021 | 141.93 | 95.53 | 202.92 |
| DALYs | Zambia | Both | Age-standardized | Rate | 2021 | 175.04 | 119.61 | 248.79 |
| DALYs | Zimbabwe | Both | Age-standardized | Rate | 2021 | 172.08 | 116.96 | 244.94 |

**Table S5.** Age-standardised prevalence rates for autism spectrum disorder among adolescents and young adults (15–39 years), by country, 2021

| **Measure** | **Location** | **Sex** | **Age** | **Cause** | **Metric** | **Year** | **Val** | **Lower** | **Upper** |
| --- | --- | --- | --- | --- | --- | --- | --- | --- | --- |
| Prevalence | Afghanistan | Both | Age-standardized | Autism spectrum disorders | Rate | 2021 | 744.0447 | 615.7895 | 886.0198 |
| Prevalence | Albania | Both | Age-standardized | Autism spectrum disorders | Rate | 2021 | 985.0142 | 815.9569 | 1163.71 |
| Prevalence | Algeria | Both | Age-standardized | Autism spectrum disorders | Rate | 2021 | 803.6981 | 671.4073 | 954.5194 |
| Prevalence | American Samoa | Both | Age-standardized | Autism spectrum disorders | Rate | 2021 | 736.7107 | 612.0429 | 879.088 |
| Prevalence | Andorra | Both | Age-standardized | Autism spectrum disorders | Rate | 2021 | 942.835 | 786.5616 | 1122.347 |
| Prevalence | Angola | Both | Age-standardized | Autism spectrum disorders | Rate | 2021 | 921.8611 | 774.358 | 1082.863 |
| Prevalence | Antigua and Barbuda | Both | Age-standardized | Autism spectrum disorders | Rate | 2021 | 743.8352 | 616.6828 | 882.6911 |
| Prevalence | Argentina | Both | Age-standardized | Autism spectrum disorders | Rate | 2021 | 1094.571 | 911.3184 | 1294.597 |
| Prevalence | Armenia | Both | Age-standardized | Autism spectrum disorders | Rate | 2021 | 962.0058 | 803.2973 | 1132.034 |
| Prevalence | Australia | Both | Age-standardized | Autism spectrum disorders | Rate | 2021 | 1230.848 | 1027.484 | 1473.809 |
| Prevalence | Austria | Both | Age-standardized | Autism spectrum disorders | Rate | 2021 | 935.1475 | 775.0663 | 1124.39 |
| Prevalence | Azerbaijan | Both | Age-standardized | Autism spectrum disorders | Rate | 2021 | 933.6568 | 780.8461 | 1099.105 |
| Prevalence | Bahamas | Both | Age-standardized | Autism spectrum disorders | Rate | 2021 | 726.4193 | 600.832 | 864.264 |
| Prevalence | Bahrain | Both | Age-standardized | Autism spectrum disorders | Rate | 2021 | 901.0796 | 750.218 | 1071.029 |
| Prevalence | Bangladesh | Both | Age-standardized | Autism spectrum disorders | Rate | 2021 | 609.4382 | 504.5784 | 722.6936 |
| Prevalence | Barbados | Both | Age-standardized | Autism spectrum disorders | Rate | 2021 | 744.702 | 625.0226 | 895.9331 |
| Prevalence | Belarus | Both | Age-standardized | Autism spectrum disorders | Rate | 2021 | 991.4526 | 824.8253 | 1183.838 |
| Prevalence | Belgium | Both | Age-standardized | Autism spectrum disorders | Rate | 2021 | 922.3151 | 768.1827 | 1095.509 |
| Prevalence | Belize | Both | Age-standardized | Autism spectrum disorders | Rate | 2021 | 712.2514 | 593.5047 | 856.0293 |
| Prevalence | Benin | Both | Age-standardized | Autism spectrum disorders | Rate | 2021 | 928.8992 | 770.5485 | 1104.772 |
| Prevalence | Bermuda | Both | Age-standardized | Autism spectrum disorders | Rate | 2021 | 765.6289 | 635.5018 | 922.5849 |
| Prevalence | Bhutan | Both | Age-standardized | Autism spectrum disorders | Rate | 2021 | 681.9683 | 568.5162 | 810.317 |
| Prevalence | Bolivia (Plurinational State of) | Both | Age-standardized | Autism spectrum disorders | Rate | 2021 | 696.5413 | 583.0245 | 835.2142 |
| Prevalence | Bosnia and Herzegovina | Both | Age-standardized | Autism spectrum disorders | Rate | 2021 | 1012.128 | 841.3559 | 1197.631 |
| Prevalence | Botswana | Both | Age-standardized | Autism spectrum disorders | Rate | 2021 | 953.3353 | 795.8747 | 1132.091 |
| Prevalence | Brazil | Both | Age-standardized | Autism spectrum disorders | Rate | 2021 | 643.7685 | 538.4455 | 766.0765 |
| Prevalence | Brunei Darussalam | Both | Age-standardized | Autism spectrum disorders | Rate | 2021 | 1445.647 | 1216.801 | 1720.765 |
| Prevalence | Bulgaria | Both | Age-standardized | Autism spectrum disorders | Rate | 2021 | 978.468 | 816.545 | 1164.77 |
| Prevalence | Burkina Faso | Both | Age-standardized | Autism spectrum disorders | Rate | 2021 | 909.5954 | 763.6902 | 1075.177 |
| Prevalence | Burundi | Both | Age-standardized | Autism spectrum disorders | Rate | 2021 | 926.4794 | 772.4888 | 1094.717 |
| Prevalence | Cabo Verde | Both | Age-standardized | Autism spectrum disorders | Rate | 2021 | 1000.627 | 832.5218 | 1191.284 |
| Prevalence | Cambodia | Both | Age-standardized | Autism spectrum disorders | Rate | 2021 | 707.6064 | 589.572 | 845.0113 |
| Prevalence | Cameroon | Both | Age-standardized | Autism spectrum disorders | Rate | 2021 | 932.409 | 781.8742 | 1106.868 |
| Prevalence | Canada | Both | Age-standardized | Autism spectrum disorders | Rate | 2021 | 1158.876 | 966.0668 | 1369.282 |
| Prevalence | Central African Republic | Both | Age-standardized | Autism spectrum disorders | Rate | 2021 | 890.6213 | 745.9862 | 1077.288 |
| Prevalence | Chad | Both | Age-standardized | Autism spectrum disorders | Rate | 2021 | 908.3533 | 755.5116 | 1091.478 |
| Prevalence | Chile | Both | Age-standardized | Autism spectrum disorders | Rate | 2021 | 1134.087 | 948.3883 | 1337.644 |
| Prevalence | China | Both | Age-standardized | Autism spectrum disorders | Rate | 2021 | 679.1406 | 565.9833 | 808.3182 |
| Prevalence | Colombia | Both | Age-standardized | Autism spectrum disorders | Rate | 2021 | 807.7954 | 672.7664 | 962.651 |
| Prevalence | Comoros | Both | Age-standardized | Autism spectrum disorders | Rate | 2021 | 953.7212 | 797.3617 | 1130.329 |
| Prevalence | Congo | Both | Age-standardized | Autism spectrum disorders | Rate | 2021 | 934.984 | 782.1628 | 1108.084 |
| Prevalence | Cook Islands | Both | Age-standardized | Autism spectrum disorders | Rate | 2021 | 737.2424 | 614.7648 | 879.2876 |
| Prevalence | Costa Rica | Both | Age-standardized | Autism spectrum disorders | Rate | 2021 | 822.5747 | 681.8124 | 984.3897 |
| Prevalence | Croatia | Both | Age-standardized | Autism spectrum disorders | Rate | 2021 | 1032.976 | 854.5973 | 1230.641 |
| Prevalence | Cuba | Both | Age-standardized | Autism spectrum disorders | Rate | 2021 | 756.3011 | 631.0172 | 898.3908 |
| Prevalence | Cyprus | Both | Age-standardized | Autism spectrum disorders | Rate | 2021 | 925.1856 | 769.7062 | 1103.761 |
| Prevalence | Czechia | Both | Age-standardized | Autism spectrum disorders | Rate | 2021 | 1033.44 | 857.8899 | 1220.122 |
| Prevalence | Côte d'Ivoire | Both | Age-standardized | Autism spectrum disorders | Rate | 2021 | 945.4322 | 786.7778 | 1130.112 |
| Prevalence | Democratic People's Republic of Korea | Both | Age-standardized | Autism spectrum disorders | Rate | 2021 | 742.1869 | 617.9615 | 884.0278 |
| Prevalence | Democratic Republic of the Congo | Both | Age-standardized | Autism spectrum disorders | Rate | 2021 | 936.4756 | 779.9374 | 1117.379 |
| Prevalence | Denmark | Both | Age-standardized | Autism spectrum disorders | Rate | 2021 | 989.5806 | 835.4303 | 1176.18 |
| Prevalence | Djibouti | Both | Age-standardized | Autism spectrum disorders | Rate | 2021 | 975.0873 | 812.7723 | 1160.38 |
| Prevalence | Dominica | Both | Age-standardized | Autism spectrum disorders | Rate | 2021 | 729.1968 | 612.7285 | 870.8499 |
| Prevalence | Dominican Republic | Both | Age-standardized | Autism spectrum disorders | Rate | 2021 | 709.4408 | 591.9418 | 845.3968 |
| Prevalence | Ecuador | Both | Age-standardized | Autism spectrum disorders | Rate | 2021 | 713.3812 | 592.8214 | 848.5204 |
| Prevalence | Egypt | Both | Age-standardized | Autism spectrum disorders | Rate | 2021 | 792.6738 | 663.1334 | 940.8448 |
| Prevalence | El Salvador | Both | Age-standardized | Autism spectrum disorders | Rate | 2021 | 779.1334 | 650.4375 | 930.0164 |
| Prevalence | Equatorial Guinea | Both | Age-standardized | Autism spectrum disorders | Rate | 2021 | 989.7056 | 824.918 | 1166.998 |
| Prevalence | Eritrea | Both | Age-standardized | Autism spectrum disorders | Rate | 2021 | 943.5017 | 785.9256 | 1119.374 |
| Prevalence | Estonia | Both | Age-standardized | Autism spectrum disorders | Rate | 2021 | 1008.444 | 847.6747 | 1198.901 |
| Prevalence | Eswatini | Both | Age-standardized | Autism spectrum disorders | Rate | 2021 | 931.7214 | 777.4884 | 1111.698 |
| Prevalence | Ethiopia | Both | Age-standardized | Autism spectrum disorders | Rate | 2021 | 932.5059 | 784.2422 | 1095.464 |
| Prevalence | Fiji | Both | Age-standardized | Autism spectrum disorders | Rate | 2021 | 716.5076 | 594.3532 | 856.0376 |
| Prevalence | Finland | Both | Age-standardized | Autism spectrum disorders | Rate | 2021 | 870.0903 | 723.5079 | 1041.342 |
| Prevalence | France | Both | Age-standardized | Autism spectrum disorders | Rate | 2021 | 918.3637 | 767.8877 | 1095.137 |
| Prevalence | Gabon | Both | Age-standardized | Autism spectrum disorders | Rate | 2021 | 940.8755 | 786.7835 | 1121.388 |
| Prevalence | Gambia | Both | Age-standardized | Autism spectrum disorders | Rate | 2021 | 933.9436 | 783.1551 | 1105.774 |
| Prevalence | Georgia | Both | Age-standardized | Autism spectrum disorders | Rate | 2021 | 957.6478 | 800.133 | 1136.748 |
| Prevalence | Germany | Both | Age-standardized | Autism spectrum disorders | Rate | 2021 | 945.776 | 780.2163 | 1128.289 |
| Prevalence | Ghana | Both | Age-standardized | Autism spectrum disorders | Rate | 2021 | 936.2032 | 783.7182 | 1114.031 |
| Prevalence | Greece | Both | Age-standardized | Autism spectrum disorders | Rate | 2021 | 920.3658 | 766.7092 | 1104.483 |
| Prevalence | Greenland | Both | Age-standardized | Autism spectrum disorders | Rate | 2021 | 1055.755 | 883.7518 | 1246.351 |
| Prevalence | Grenada | Both | Age-standardized | Autism spectrum disorders | Rate | 2021 | 734.4221 | 611.953 | 869.6227 |
| Prevalence | Guam | Both | Age-standardized | Autism spectrum disorders | Rate | 2021 | 757.9425 | 625.6155 | 907.0543 |
| Prevalence | Guatemala | Both | Age-standardized | Autism spectrum disorders | Rate | 2021 | 758.3156 | 631.2477 | 899.6923 |
| Prevalence | Guinea | Both | Age-standardized | Autism spectrum disorders | Rate | 2021 | 905.1089 | 756.0673 | 1077.115 |
| Prevalence | Guinea-Bissau | Both | Age-standardized | Autism spectrum disorders | Rate | 2021 | 904.307 | 757.2248 | 1084.225 |
| Prevalence | Guyana | Both | Age-standardized | Autism spectrum disorders | Rate | 2021 | 697.1041 | 583.4217 | 829.8134 |
| Prevalence | Haiti | Both | Age-standardized | Autism spectrum disorders | Rate | 2021 | 660.7291 | 550.8932 | 787.2813 |
| Prevalence | Honduras | Both | Age-standardized | Autism spectrum disorders | Rate | 2021 | 759.7657 | 634.1378 | 901.4774 |
| Prevalence | Hungary | Both | Age-standardized | Autism spectrum disorders | Rate | 2021 | 1012.349 | 842.8682 | 1199.963 |
| Prevalence | Iceland | Both | Age-standardized | Autism spectrum disorders | Rate | 2021 | 1091.85 | 914.7311 | 1288.557 |
| Prevalence | India | Both | Age-standardized | Autism spectrum disorders | Rate | 2021 | 743.1348 | 624.9035 | 870.8884 |
| Prevalence | Indonesia | Both | Age-standardized | Autism spectrum disorders | Rate | 2021 | 675.4668 | 567.5342 | 802.1567 |
| Prevalence | Iran (Islamic Republic of) | Both | Age-standardized | Autism spectrum disorders | Rate | 2021 | 819.9601 | 687.6263 | 967.5085 |
| Prevalence | Iraq | Both | Age-standardized | Autism spectrum disorders | Rate | 2021 | 740.1656 | 619.0746 | 889.4425 |
| Prevalence | Ireland | Both | Age-standardized | Autism spectrum disorders | Rate | 2021 | 1110.457 | 927.5478 | 1321.095 |
| Prevalence | Israel | Both | Age-standardized | Autism spectrum disorders | Rate | 2021 | 831.1958 | 695.5057 | 989.4579 |
| Prevalence | Italy | Both | Age-standardized | Autism spectrum disorders | Rate | 2021 | 1044.463 | 874.8524 | 1241.233 |
| Prevalence | Jamaica | Both | Age-standardized | Autism spectrum disorders | Rate | 2021 | 733.6046 | 616.6916 | 870.0273 |
| Prevalence | Japan | Both | Age-standardized | Autism spectrum disorders | Rate | 2021 | 1644.006 | 1385.281 | 1930.307 |
| Prevalence | Jordan | Both | Age-standardized | Autism spectrum disorders | Rate | 2021 | 845.9224 | 705.1415 | 1003.393 |
| Prevalence | Kazakhstan | Both | Age-standardized | Autism spectrum disorders | Rate | 2021 | 939.7131 | 788.956 | 1114.139 |
| Prevalence | Kenya | Both | Age-standardized | Autism spectrum disorders | Rate | 2021 | 946.6138 | 795.597 | 1109.612 |
| Prevalence | Kiribati | Both | Age-standardized | Autism spectrum disorders | Rate | 2021 | 680.1304 | 566.9649 | 813.6359 |
| Prevalence | Kuwait | Both | Age-standardized | Autism spectrum disorders | Rate | 2021 | 845.6866 | 700.4506 | 1004.45 |
| Prevalence | Kyrgyzstan | Both | Age-standardized | Autism spectrum disorders | Rate | 2021 | 931.6874 | 782.9956 | 1114.061 |
| Prevalence | Lao People's Democratic Republic | Both | Age-standardized | Autism spectrum disorders | Rate | 2021 | 698.7637 | 583.7742 | 840.0271 |
| Prevalence | Latvia | Both | Age-standardized | Autism spectrum disorders | Rate | 2021 | 989.5035 | 816.7824 | 1171.707 |
| Prevalence | Lebanon | Both | Age-standardized | Autism spectrum disorders | Rate | 2021 | 913.1732 | 761.4441 | 1079.954 |
| Prevalence | Lesotho | Both | Age-standardized | Autism spectrum disorders | Rate | 2021 | 925.4275 | 774.0376 | 1102.62 |
| Prevalence | Liberia | Both | Age-standardized | Autism spectrum disorders | Rate | 2021 | 940.1056 | 782.66 | 1118.05 |
| Prevalence | Libya | Both | Age-standardized | Autism spectrum disorders | Rate | 2021 | 800.6854 | 665.7473 | 950.6765 |
| Prevalence | Lithuania | Both | Age-standardized | Autism spectrum disorders | Rate | 2021 | 992.0994 | 824.9123 | 1172.28 |
| Prevalence | Luxembourg | Both | Age-standardized | Autism spectrum disorders | Rate | 2021 | 938.1047 | 781.1758 | 1127.188 |
| Prevalence | Madagascar | Both | Age-standardized | Autism spectrum disorders | Rate | 2021 | 931.8011 | 778.3409 | 1105.624 |
| Prevalence | Malawi | Both | Age-standardized | Autism spectrum disorders | Rate | 2021 | 927.3441 | 777.749 | 1097.303 |
| Prevalence | Malaysia | Both | Age-standardized | Autism spectrum disorders | Rate | 2021 | 764.8817 | 636.9541 | 915.7326 |
| Prevalence | Maldives | Both | Age-standardized | Autism spectrum disorders | Rate | 2021 | 818.155 | 673.442 | 976.3281 |
| Prevalence | Mali | Both | Age-standardized | Autism spectrum disorders | Rate | 2021 | 917.6314 | 766.7685 | 1093.473 |
| Prevalence | Malta | Both | Age-standardized | Autism spectrum disorders | Rate | 2021 | 937.1097 | 785.1498 | 1116.168 |
| Prevalence | Marshall Islands | Both | Age-standardized | Autism spectrum disorders | Rate | 2021 | 710.4564 | 591.1826 | 848.7586 |
| Prevalence | Mauritania | Both | Age-standardized | Autism spectrum disorders | Rate | 2021 | 949.2784 | 795.6109 | 1124.075 |
| Prevalence | Mauritius | Both | Age-standardized | Autism spectrum disorders | Rate | 2021 | 757.8284 | 627.0126 | 898.7269 |
| Prevalence | Mexico | Both | Age-standardized | Autism spectrum disorders | Rate | 2021 | 792.3439 | 669.187 | 941.2153 |
| Prevalence | Micronesia (Federated States of) | Both | Age-standardized | Autism spectrum disorders | Rate | 2021 | 717.324 | 602.4214 | 846.6302 |
| Prevalence | Monaco | Both | Age-standardized | Autism spectrum disorders | Rate | 2021 | 931.4969 | 777.7444 | 1113.721 |
| Prevalence | Mongolia | Both | Age-standardized | Autism spectrum disorders | Rate | 2021 | 905.7275 | 761.9942 | 1082.983 |
| Prevalence | Montenegro | Both | Age-standardized | Autism spectrum disorders | Rate | 2021 | 1015.511 | 847.8467 | 1193.429 |
| Prevalence | Morocco | Both | Age-standardized | Autism spectrum disorders | Rate | 2021 | 788.4012 | 661.5483 | 934.1895 |
| Prevalence | Mozambique | Both | Age-standardized | Autism spectrum disorders | Rate | 2021 | 913.689 | 763.3613 | 1090.961 |
| Prevalence | Myanmar | Both | Age-standardized | Autism spectrum disorders | Rate | 2021 | 698.8592 | 582.0931 | 837.9334 |
| Prevalence | Namibia | Both | Age-standardized | Autism spectrum disorders | Rate | 2021 | 952.1857 | 797.0019 | 1135.236 |
| Prevalence | Nauru | Both | Age-standardized | Autism spectrum disorders | Rate | 2021 | 704.9532 | 586.9825 | 842.6141 |
| Prevalence | Nepal | Both | Age-standardized | Autism spectrum disorders | Rate | 2021 | 648.2887 | 538.7909 | 779.8023 |
| Prevalence | Netherlands | Both | Age-standardized | Autism spectrum disorders | Rate | 2021 | 936.6374 | 771.0107 | 1118.268 |
| Prevalence | New Zealand | Both | Age-standardized | Autism spectrum disorders | Rate | 2021 | 1212.71 | 1005.06 | 1457.546 |
| Prevalence | Nicaragua | Both | Age-standardized | Autism spectrum disorders | Rate | 2021 | 792.3729 | 656.624 | 935.1216 |
| Prevalence | Niger | Both | Age-standardized | Autism spectrum disorders | Rate | 2021 | 920.8669 | 767.3624 | 1097.303 |
| Prevalence | Nigeria | Both | Age-standardized | Autism spectrum disorders | Rate | 2021 | 918.2995 | 773.7671 | 1079.886 |
| Prevalence | Niue | Both | Age-standardized | Autism spectrum disorders | Rate | 2021 | 730.8533 | 611.3401 | 861.789 |
| Prevalence | North Macedonia | Both | Age-standardized | Autism spectrum disorders | Rate | 2021 | 995.8895 | 834.3688 | 1183.049 |
| Prevalence | Northern Mariana Islands | Both | Age-standardized | Autism spectrum disorders | Rate | 2021 | 759.9193 | 633.6118 | 903.2991 |
| Prevalence | Norway | Both | Age-standardized | Autism spectrum disorders | Rate | 2021 | 842.6782 | 708.5026 | 1004.84 |
| Prevalence | Oman | Both | Age-standardized | Autism spectrum disorders | Rate | 2021 | 883.6463 | 731.6057 | 1058.875 |
| Prevalence | Pakistan | Both | Age-standardized | Autism spectrum disorders | Rate | 2021 | 659.9533 | 551.3794 | 779.4677 |
| Prevalence | Palau | Both | Age-standardized | Autism spectrum disorders | Rate | 2021 | 774.8113 | 642.7367 | 913.5505 |
| Prevalence | Palestine | Both | Age-standardized | Autism spectrum disorders | Rate | 2021 | 814.3793 | 678.3785 | 972.4996 |
| Prevalence | Panama | Both | Age-standardized | Autism spectrum disorders | Rate | 2021 | 812.1214 | 680.2614 | 961.2685 |
| Prevalence | Papua New Guinea | Both | Age-standardized | Autism spectrum disorders | Rate | 2021 | 695.8208 | 583.6322 | 833.7739 |
| Prevalence | Paraguay | Both | Age-standardized | Autism spectrum disorders | Rate | 2021 | 657.9914 | 546.4565 | 785.0666 |
| Prevalence | Peru | Both | Age-standardized | Autism spectrum disorders | Rate | 2021 | 720.7237 | 602.3294 | 859.7486 |
| Prevalence | Philippines | Both | Age-standardized | Autism spectrum disorders | Rate | 2021 | 716.5934 | 601.971 | 846.2606 |
| Prevalence | Poland | Both | Age-standardized | Autism spectrum disorders | Rate | 2021 | 1004.35 | 841.1455 | 1188.789 |
| Prevalence | Portugal | Both | Age-standardized | Autism spectrum disorders | Rate | 2021 | 902.1935 | 753.9319 | 1069.582 |
| Prevalence | Puerto Rico | Both | Age-standardized | Autism spectrum disorders | Rate | 2021 | 760.796 | 626.4125 | 911.1094 |
| Prevalence | Qatar | Both | Age-standardized | Autism spectrum disorders | Rate | 2021 | 1029.631 | 854.2912 | 1221.74 |
| Prevalence | Republic of Korea | Both | Age-standardized | Autism spectrum disorders | Rate | 2021 | 1571.191 | 1314.127 | 1856.423 |
| Prevalence | Republic of Moldova | Both | Age-standardized | Autism spectrum disorders | Rate | 2021 | 963.2022 | 805.1863 | 1139.352 |
| Prevalence | Romania | Both | Age-standardized | Autism spectrum disorders | Rate | 2021 | 978.5455 | 822.0035 | 1154.015 |
| Prevalence | Russian Federation | Both | Age-standardized | Autism spectrum disorders | Rate | 2021 | 974.2032 | 817.4026 | 1150.648 |
| Prevalence | Rwanda | Both | Age-standardized | Autism spectrum disorders | Rate | 2021 | 939.9715 | 786.4882 | 1112.85 |
| Prevalence | Saint Kitts and Nevis | Both | Age-standardized | Autism spectrum disorders | Rate | 2021 | 726.9005 | 609.104 | 859.9568 |
| Prevalence | Saint Lucia | Both | Age-standardized | Autism spectrum disorders | Rate | 2021 | 736.679 | 611.3017 | 878.8312 |
| Prevalence | Saint Vincent and the Grenadines | Both | Age-standardized | Autism spectrum disorders | Rate | 2021 | 725.9286 | 603.7879 | 862.8298 |
| Prevalence | Samoa | Both | Age-standardized | Autism spectrum disorders | Rate | 2021 | 724.4555 | 604.114 | 860.8941 |
| Prevalence | San Marino | Both | Age-standardized | Autism spectrum disorders | Rate | 2021 | 925.3099 | 770.2321 | 1106.378 |
| Prevalence | Sao Tome and Principe | Both | Age-standardized | Autism spectrum disorders | Rate | 2021 | 965.0938 | 803.9961 | 1137.122 |
| Prevalence | Saudi Arabia | Both | Age-standardized | Autism spectrum disorders | Rate | 2021 | 853.6724 | 705.8995 | 1014.45 |
| Prevalence | Senegal | Both | Age-standardized | Autism spectrum disorders | Rate | 2021 | 942.2244 | 786.4008 | 1117.205 |
| Prevalence | Serbia | Both | Age-standardized | Autism spectrum disorders | Rate | 2021 | 1009.674 | 837.8611 | 1204.605 |
| Prevalence | Seychelles | Both | Age-standardized | Autism spectrum disorders | Rate | 2021 | 772.447 | 640.0238 | 919.3213 |
| Prevalence | Sierra Leone | Both | Age-standardized | Autism spectrum disorders | Rate | 2021 | 925.3656 | 772.7112 | 1104.202 |
| Prevalence | Singapore | Both | Age-standardized | Autism spectrum disorders | Rate | 2021 | 1530.949 | 1292.511 | 1815.02 |
| Prevalence | Slovakia | Both | Age-standardized | Autism spectrum disorders | Rate | 2021 | 1006.485 | 844.8188 | 1199.481 |
| Prevalence | Slovenia | Both | Age-standardized | Autism spectrum disorders | Rate | 2021 | 1052.754 | 875.3892 | 1262.501 |
| Prevalence | Solomon Islands | Both | Age-standardized | Autism spectrum disorders | Rate | 2021 | 699.5864 | 586.5285 | 832.0892 |
| Prevalence | Somalia | Both | Age-standardized | Autism spectrum disorders | Rate | 2021 | 924.8254 | 775.3433 | 1089.661 |
| Prevalence | South Africa | Both | Age-standardized | Autism spectrum disorders | Rate | 2021 | 961.1329 | 808.1895 | 1140.466 |
| Prevalence | South Sudan | Both | Age-standardized | Autism spectrum disorders | Rate | 2021 | 920.8675 | 770.9577 | 1097.09 |
| Prevalence | Spain | Both | Age-standardized | Autism spectrum disorders | Rate | 2021 | 1065.73 | 899.6125 | 1250.058 |
| Prevalence | Sri Lanka | Both | Age-standardized | Autism spectrum disorders | Rate | 2021 | 775.4059 | 640.705 | 924.5262 |
| Prevalence | Sudan | Both | Age-standardized | Autism spectrum disorders | Rate | 2021 | 764.1305 | 635.9839 | 911.063 |
| Prevalence | Suriname | Both | Age-standardized | Autism spectrum disorders | Rate | 2021 | 706.4123 | 589.6736 | 840.0773 |
| Prevalence | Sweden | Both | Age-standardized | Autism spectrum disorders | Rate | 2021 | 1065.467 | 882.4646 | 1271.693 |
| Prevalence | Switzerland | Both | Age-standardized | Autism spectrum disorders | Rate | 2021 | 940.5454 | 781.4904 | 1122.965 |
| Prevalence | Syrian Arab Republic | Both | Age-standardized | Autism spectrum disorders | Rate | 2021 | 761.5053 | 632.1252 | 913.0565 |
| Prevalence | Taiwan (Province of China) | Both | Age-standardized | Autism spectrum disorders | Rate | 2021 | 938.8558 | 774.0122 | 1116.235 |
| Prevalence | Tajikistan | Both | Age-standardized | Autism spectrum disorders | Rate | 2021 | 906.6964 | 759.5185 | 1067.613 |
| Prevalence | Thailand | Both | Age-standardized | Autism spectrum disorders | Rate | 2021 | 744.5112 | 618.513 | 890.4007 |
| Prevalence | Timor-Leste | Both | Age-standardized | Autism spectrum disorders | Rate | 2021 | 700.3215 | 584.009 | 833.0237 |
| Prevalence | Togo | Both | Age-standardized | Autism spectrum disorders | Rate | 2021 | 928.7183 | 780.5402 | 1107.565 |
| Prevalence | Tokelau | Both | Age-standardized | Autism spectrum disorders | Rate | 2021 | 744.4352 | 625.6162 | 891.2776 |
| Prevalence | Tonga | Both | Age-standardized | Autism spectrum disorders | Rate | 2021 | 718.1847 | 596.5988 | 858.1225 |
| Prevalence | Trinidad and Tobago | Both | Age-standardized | Autism spectrum disorders | Rate | 2021 | 731.9942 | 615.8514 | 872.0729 |
| Prevalence | Tunisia | Both | Age-standardized | Autism spectrum disorders | Rate | 2021 | 811.4241 | 672.5859 | 960.8699 |
| Prevalence | Turkmenistan | Both | Age-standardized | Autism spectrum disorders | Rate | 2021 | 935.6382 | 784.2804 | 1102.714 |
| Prevalence | Tuvalu | Both | Age-standardized | Autism spectrum disorders | Rate | 2021 | 720.5669 | 596.7581 | 861.967 |
| Prevalence | Turkey | Both | Age-standardized | Autism spectrum disorders | Rate | 2021 | 818.0908 | 679.0054 | 966.9464 |
| Prevalence | Uganda | Both | Age-standardized | Autism spectrum disorders | Rate | 2021 | 942.7658 | 788.2477 | 1116.946 |
| Prevalence | Ukraine | Both | Age-standardized | Autism spectrum disorders | Rate | 2021 | 970.4483 | 810.8493 | 1156.867 |
| Prevalence | United Arab Emirates | Both | Age-standardized | Autism spectrum disorders | Rate | 2021 | 868.631 | 727.6465 | 1032.508 |
| Prevalence | United Kingdom | Both | Age-standardized | Autism spectrum disorders | Rate | 2021 | 780.7638 | 655.8804 | 922.4644 |
| Prevalence | United Republic of Tanzania | Both | Age-standardized | Autism spectrum disorders | Rate | 2021 | 934.9685 | 782.4105 | 1108.469 |
| Prevalence | United States of America | Both | Age-standardized | Autism spectrum disorders | Rate | 2021 | 1139.791 | 960.6093 | 1340.113 |
| Prevalence | United States Virgin Islands | Both | Age-standardized | Autism spectrum disorders | Rate | 2021 | 737.9297 | 611.5252 | 884.083 |
| Prevalence | Uruguay | Both | Age-standardized | Autism spectrum disorders | Rate | 2021 | 1106.995 | 921.4015 | 1325.794 |
| Prevalence | Uzbekistan | Both | Age-standardized | Autism spectrum disorders | Rate | 2021 | 920.3787 | 767.0717 | 1093.391 |
| Prevalence | Vanuatu | Both | Age-standardized | Autism spectrum disorders | Rate | 2021 | 698.2938 | 578.1059 | 836.7243 |
| Prevalence | Venezuela (Bolivarian Republic of) | Both | Age-standardized | Autism spectrum disorders | Rate | 2021 | 781.8691 | 655.3466 | 927.7629 |
| Prevalence | Viet Nam | Both | Age-standardized | Autism spectrum disorders | Rate | 2021 | 757.2843 | 633.9774 | 903.4519 |
| Prevalence | Yemen | Both | Age-standardized | Autism spectrum disorders | Rate | 2021 | 759.2717 | 638.0047 | 903.3978 |
| Prevalence | Zambia | Both | Age-standardized | Autism spectrum disorders | Rate | 2021 | 935.224 | 779.6426 | 1110.275 |
| Prevalence | Zimbabwe | Both | Age-standardized | Autism spectrum disorders | Rate | 2021 | 918.4707 | 768.4213 | 1093.866 |

**Table S6.** Estimated annual percentage change (EAPC) in age-standardised prevalence and DALY rates for autism spectrum disorder among adolescents and young adults, by country, 1990–2021

| **Location** | **EAPCs of ASPR (95%CI)** | **EAPCs of ASDR (95%CI)** |
| --- | --- | --- |
| Afghanistan | 0.16(0.14,0.19) | 0.23(0.20,0.26) |
| Albania | 0.15(0.13,0.16) | 0.16(0.14,0.18) |
| Algeria | 0.03(0.03,0.04) | 0.04(0.03,0.04) |
| American Samoa | -0.01(-0.02,-0.00) | -0.01(-0.02,-0.00) |
| Andorra | -0.05(-0.08,-0.03) | -0.05(-0.07,-0.03) |
| Angola | 0.01(-0.00,0.03) | 0.04(0.02,0.06) |
| Antigua and Barbuda | 0.05(0.03,0.07) | 0.05(0.03,0.08) |
| Argentina | 0.01(-0.00,0.02) | 0.01(0.00,0.02) |
| Armenia | 0.05(0.04,0.07) | 0.07(0.05,0.08) |
| Australia | 0.07(0.06,0.07) | 0.08(0.07,0.08) |
| Austria | 0.01(0.00,0.03) | 0.02(0.00,0.03) |
| Azerbaijan | 0.09(0.07,0.10) | 0.10(0.09,0.12) |
| Bahamas | -0.00(-0.01,0.00) | -0.00(-0.01,0.01) |
| Bahrain | 0.20(0.17,0.22) | 0.20(0.17,0.23) |
| Bangladesh | -0.02(-0.03,-0.00) | 0.01(-0.01,0.02) |
| Barbados | 0.00(-0.00,0.01) | 0.01(0.00,0.02) |
| Belarus | 0.09(0.08,0.10) | 0.10(0.09,0.11) |
| Belgium | -0.02(-0.03,-0.01) | -0.02(-0.03,-0.01) |
| Belize | -0.02(-0.03,-0.01) | -0.02(-0.03,-0.01) |
| Benin | 0.10(0.10,0.11) | 0.13(0.12,0.14) |
| Bermuda | 0.02(0.02,0.03) | 0.02(0.02,0.03) |
| Bhutan | 0.04(0.03,0.05) | 0.05(0.04,0.07) |
| Bolivia (Plurinational State of) | 0.08(0.08,0.08) | 0.09(0.08,0.10) |
| Bosnia and Herzegovina | 0.07(0.05,0.09) | 0.09(0.06,0.11) |
| Botswana | 0.12(0.11,0.13) | 0.14(0.12,0.15) |
| Brazil | 0.02(0.02,0.03) | 0.04(0.03,0.04) |
| Brunei Darussalam | 0.05(0.02,0.08) | 0.06(0.03,0.09) |
| Bulgaria | 0.04(0.03,0.05) | 0.05(0.04,0.06) |
| Burkina Faso | 0.06(0.06,0.07) | 0.11(0.10,0.12) |
| Burundi | 0.09(0.07,0.10) | 0.09(0.07,0.11) |
| Cabo Verde | 0.19(0.17,0.20) | 0.19(0.17,0.21) |
| Cambodia | 0.18(0.16,0.19) | 0.23(0.21,0.25) |
| Cameroon | 0.08(0.07,0.09) | 0.11(0.10,0.13) |
| Canada | 0.04(0.03,0.04) | 0.03(0.03,0.04) |
| Central African Republic | -0.02(-0.03,-0.00) | 0.01(-0.00,0.03) |
| Chad | 0.03(0.02,0.04) | 0.05(0.04,0.06) |
| Chile | 0.08(0.07,0.09) | 0.08(0.07,0.09) |
| China | 0.23(0.20,0.25) | 0.24(0.22,0.26) |
| Colombia | 0.10(0.10,0.11) | 0.12(0.11,0.12) |
| Comoros | 0.08(0.07,0.09) | 0.10(0.09,0.11) |
| Congo | 0.04(0.04,0.05) | 0.06(0.05,0.07) |
| Cook Islands | -0.08(-0.10,-0.07) | -0.09(-0.11,-0.08) |
| Costa Rica | -0.00(-0.00,0.00) | -0.00(-0.01,0.00) |
| Croatia | 0.07(0.07,0.08) | 0.09(0.08,0.09) |
| Cuba | 0.04(0.03,0.04) | 0.05(0.04,0.06) |
| Cyprus | 0.01(0.01,0.02) | 0.02(0.01,0.02) |
| Czechia | 0.07(0.06,0.08) | 0.08(0.07,0.09) |
| Côte d'Ivoire | 0.06(0.05,0.07) | 0.10(0.09,0.10) |
| Democratic People's Republic of Korea | 0.15(0.13,0.17) | 0.16(0.14,0.19) |
| Democratic Republic of the Congo | 0.06(0.06,0.07) | 0.10(0.09,0.11) |
| Denmark | 0.04(0.01,0.07) | 0.05(0.02,0.07) |
| Djibouti | 0.04(0.02,0.06) | 0.05(0.03,0.08) |
| Dominica | -0.03(-0.04,-0.02) | -0.03(-0.04,-0.01) |
| Dominican Republic | 0.08(0.07,0.09) | 0.09(0.07,0.10) |
| Ecuador | 0.07(0.06,0.07) | 0.07(0.07,0.08) |
| Egypt | 0.03(0.02,0.03) | 0.05(0.04,0.05) |
| El Salvador | 0.06(0.05,0.07) | 0.11(0.10,0.11) |
| Equatorial Guinea | 0.40(0.38,0.42) | 0.46(0.44,0.49) |
| Eritrea | 0.11(0.10,0.12) | 0.20(0.19,0.21) |
| Estonia | 0.12(0.11,0.14) | 0.14(0.13,0.15) |
| Eswatini | 0.10(0.08,0.11) | 0.06(0.04,0.09) |
| Ethiopia | 0.12(0.12,0.13) | 0.16(0.15,0.16) |
| Fiji | -0.01(-0.01,-0.00) | 0.01(-0.00,0.02) |
| Finland | 0.00(-0.01,0.01) | 0.00(-0.01,0.01) |
| France | 0.01(0.01,0.02) | 0.02(0.01,0.03) |
| Gabon | 0.00(-0.01,0.01) | 0.02(0.01,0.03) |
| Gambia | 0.03(0.03,0.04) | 0.03(0.02,0.04) |
| Georgia | 0.04(0.03,0.05) | 0.03(0.02,0.04) |
| Germany | 0.02(-0.00,0.04) | 0.02(-0.00,0.04) |
| Ghana | 0.05(0.04,0.05) | 0.07(0.07,0.08) |
| Greece | -0.03(-0.05,-0.01) | -0.03(-0.05,-0.01) |
| Greenland | -0.03(-0.04,-0.02) | -0.02(-0.03,-0.01) |
| Grenada | 0.03(0.03,0.04) | 0.04(0.04,0.05) |
| Guam | -0.05(-0.06,-0.03) | -0.04(-0.06,-0.02) |
| Guatemala | 0.05(0.05,0.05) | 0.09(0.08,0.09) |
| Guinea | 0.00(-0.01,0.02) | 0.02(0.01,0.03) |
| Guinea-Bissau | 0.04(0.04,0.05) | 0.06(0.06,0.07) |
| Guyana | -0.01(-0.02,0.00) | -0.01(-0.02,0.00) |
| Haiti | 0.04(0.03,0.05) | 0.06(0.05,0.07) |
| Honduras | -0.01(-0.02,-0.01) | -0.01(-0.01,-0.00) |
| Hungary | 0.06(0.05,0.07) | 0.08(0.07,0.09) |
| Iceland | 0.06(0.05,0.07) | 0.06(0.05,0.07) |
| India | 0.02(0.01,0.02) | 0.04(0.03,0.05) |
| Indonesia | 0.09(0.08,0.11) | 0.11(0.10,0.12) |
| Iran (Islamic Republic of) | 0.06(0.05,0.07) | 0.07(0.06,0.09) |
| Iraq | 0.03(0.02,0.03) | 0.06(0.05,0.07) |
| Ireland | 0.09(0.06,0.11) | 0.08(0.06,0.11) |
| Israel | -0.02(-0.03,-0.00) | -0.01(-0.02,0.00) |
| Italy | 0.06(0.04,0.07) | 0.07(0.05,0.08) |
| Jamaica | 0.02(0.02,0.02) | 0.02(0.02,0.02) |
| Japan | 0.26(0.22,0.29) | 0.26(0.23,0.29) |
| Jordan | 0.07(0.05,0.09) | 0.07(0.05,0.09) |
| Kazakhstan | 0.05(0.03,0.07) | 0.07(0.05,0.09) |
| Kenya | 0.01(-0.00,0.01) | 0.03(0.02,0.04) |
| Kiribati | -0.00(-0.01,0.00) | 0.02(0.01,0.03) |
| Kuwait | -0.11(-0.16,-0.07) | -0.12(-0.16,-0.07) |
| Kyrgyzstan | 0.04(0.03,0.04) | 0.06(0.05,0.06) |
| Lao People's Democratic Republic | 0.13(0.12,0.15) | 0.16(0.14,0.17) |
| Latvia | 0.05(0.05,0.06) | 0.07(0.06,0.08) |
| Lebanon | 0.15(0.13,0.17) | 0.18(0.16,0.20) |
| Lesotho | 0.12(0.12,0.13) | 0.10(0.09,0.10) |
| Liberia | 0.19(0.16,0.21) | 0.24(0.21,0.26) |
| Libya | 0.01(-0.01,0.03) | 0.01(-0.01,0.03) |
| Lithuania | 0.03(0.03,0.04) | 0.05(0.04,0.05) |
| Luxembourg | 0.01(0.01,0.02) | 0.03(0.02,0.03) |
| Madagascar | 0.02(0.01,0.02) | 0.05(0.05,0.06) |
| Malawi | 0.03(0.02,0.03) | 0.08(0.06,0.09) |
| Malaysia | 0.11(0.10,0.12) | 0.12(0.11,0.13) |
| Maldives | 0.48(0.41,0.54) | 0.50(0.44,0.56) |
| Mali | 0.05(0.04,0.06) | 0.07(0.06,0.08) |
| Malta | 0.02(0.01,0.03) | 0.02(0.01,0.03) |
| Marshall Islands | 0.02(0.01,0.02) | 0.01(0.00,0.02) |
| Mauritania | 0.08(0.07,0.08) | 0.10(0.09,0.10) |
| Mauritius | 0.02(0.01,0.03) | 0.02(0.01,0.03) |
| Mexico | 0.05(0.05,0.06) | 0.05(0.04,0.06) |
| Micronesia (Federated States of) | 0.05(0.04,0.06) | 0.06(0.04,0.07) |
| Monaco | -0.04(-0.06,-0.03) | -0.05(-0.06,-0.03) |
| Mongolia | 0.07(0.06,0.08) | 0.08(0.06,0.09) |
| Montenegro | 0.03(0.02,0.05) | 0.04(0.03,0.06) |
| Morocco | 0.03(0.02,0.04) | 0.03(0.02,0.05) |
| Mozambique | 0.05(0.04,0.06) | 0.08(0.07,0.09) |
| Myanmar | 0.09(0.08,0.10) | 0.12(0.10,0.13) |
| Namibia | 0.08(0.08,0.09) | 0.10(0.09,0.11) |
| Nauru | 0.01(-0.00,0.01) | 0.01(-0.00,0.02) |
| Nepal | -0.04(-0.06,-0.03) | -0.01(-0.03,0.01) |
| Netherlands | -0.02(-0.02,-0.01) | -0.02(-0.02,-0.01) |
| New Zealand | 0.08(0.07,0.10) | 0.10(0.09,0.11) |
| Nicaragua | 0.09(0.08,0.10) | 0.13(0.12,0.14) |
| Niger | 0.06(0.05,0.06) | 0.09(0.08,0.10) |
| Nigeria | 0.01(0.01,0.02) | 0.05(0.04,0.06) |
| Niue | -0.01(-0.02,0.01) | -0.00(-0.02,0.01) |
| North Macedonia | 0.08(0.07,0.09) | 0.09(0.08,0.10) |
| Northern Mariana Islands | 0.11(0.04,0.18) | 0.11(0.04,0.19) |
| Norway | 0.01(-0.00,0.03) | 0.02(0.00,0.04) |
| Oman | 0.05(0.01,0.10) | 0.06(0.01,0.10) |
| Pakistan | -0.04(-0.05,-0.03) | -0.04(-0.04,-0.03) |
| Palau | 0.15(0.11,0.19) | 0.14(0.10,0.18) |
| Palestine | -0.00(-0.01,0.01) | -0.00(-0.02,0.01) |
| Panama | 0.04(0.03,0.04) | 0.04(0.03,0.05) |
| Papua New Guinea | -0.03(-0.03,-0.02) | -0.02(-0.02,-0.01) |
| Paraguay | 0.03(0.03,0.04) | 0.04(0.03,0.05) |
| Peru | 0.11(0.10,0.11) | 0.12(0.11,0.12) |
| Philippines | 0.03(0.02,0.04) | 0.05(0.04,0.06) |
| Poland | 0.07(0.06,0.07) | 0.08(0.08,0.09) |
| Portugal | 0.02(0.01,0.03) | 0.03(0.02,0.05) |
| Puerto Rico | 0.05(0.04,0.06) | 0.06(0.05,0.07) |
| Qatar | 0.30(0.23,0.37) | 0.30(0.22,0.38) |
| Republic of Korea | 0.22(0.21,0.24) | 0.23(0.22,0.25) |
| Republic of Moldova | 0.07(0.06,0.07) | 0.08(0.08,0.09) |
| Romania | 0.09(0.09,0.10) | 0.11(0.10,0.12) |
| Russian Federation | 0.07(0.06,0.09) | 0.09(0.08,0.11) |
| Rwanda | 0.14(0.11,0.18) | 0.17(0.12,0.22) |
| Saint Kitts and Nevis | 0.03(0.02,0.04) | 0.03(0.02,0.04) |
| Saint Lucia | 0.06(0.05,0.06) | 0.06(0.05,0.07) |
| Saint Vincent and the Grenadines | -0.01(-0.02,-0.00) | -0.01(-0.02,-0.01) |
| Samoa | -0.04(-0.05,-0.03) | -0.04(-0.05,-0.03) |
| San Marino | -0.00(-0.02,0.02) | -0.01(-0.03,0.02) |
| Sao Tome and Principe | 0.10(0.09,0.11) | 0.10(0.09,0.12) |
| Saudi Arabia | 0.06(0.04,0.08) | 0.07(0.05,0.08) |
| Senegal | 0.09(0.08,0.09) | 0.11(0.10,0.11) |
| Serbia | 0.09(0.08,0.10) | 0.10(0.08,0.11) |
| Seychelles | 0.16(0.14,0.18) | 0.17(0.15,0.19) |
| Sierra Leone | 0.10(0.09,0.12) | 0.13(0.11,0.16) |
| Singapore | 0.07(0.04,0.09) | 0.07(0.04,0.10) |
| Slovakia | 0.06(0.05,0.06) | 0.07(0.06,0.08) |
| Slovenia | 0.12(0.12,0.13) | 0.13(0.12,0.15) |
| Solomon Islands | -0.02(-0.03,-0.02) | -0.01(-0.02,-0.01) |
| Somalia | 0.04(0.03,0.05) | 0.05(0.04,0.07) |
| South Africa | 0.10(0.08,0.12) | 0.09(0.07,0.12) |
| South Sudan | -0.06(-0.07,-0.05) | -0.05(-0.07,-0.04) |
| Spain | 0.12(0.08,0.16) | 0.12(0.08,0.16) |
| Sri Lanka | 0.07(0.05,0.08) | 0.08(0.06,0.10) |
| Sudan | 0.03(0.02,0.04) | 0.04(0.03,0.05) |
| Suriname | -0.04(-0.04,-0.03) | -0.04(-0.05,-0.03) |
| Sweden | 0.11(0.10,0.13) | 0.11(0.09,0.13) |
| Switzerland | 0.00(-0.01,0.01) | 0.02(0.01,0.03) |
| Syrian Arab Republic | -0.13(-0.20,-0.06) | -0.16(-0.24,-0.07) |
| Taiwan (Province of China) | 0.17(0.14,0.20) | 0.17(0.13,0.20) |
| Tajikistan | 0.01(-0.00,0.01) | 0.02(0.01,0.03) |
| Thailand | 0.08(0.07,0.09) | 0.10(0.09,0.11) |
| Timor-Leste | 0.00(-0.01,0.02) | 0.06(0.04,0.08) |
| Togo | 0.05(0.04,0.06) | 0.07(0.06,0.09) |
| Tokelau | 0.07(0.04,0.10) | 0.07(0.04,0.10) |
| Tonga | -0.05(-0.07,-0.03) | -0.05(-0.07,-0.03) |
| Trinidad and Tobago | 0.02(0.02,0.03) | 0.02(0.01,0.02) |
| Tunisia | 0.01(0.00,0.01) | 0.01(0.00,0.01) |
| Turkmenistan | 0.10(0.09,0.10) | 0.10(0.10,0.11) |
| Tuvalu | 0.19(0.17,0.21) | 0.20(0.19,0.22) |
| Turkey | 0.07(0.06,0.08) | 0.07(0.06,0.08) |
| Uganda | 0.06(0.05,0.06) | 0.11(0.10,0.12) |
| Ukraine | 0.04(0.03,0.04) | 0.04(0.03,0.05) |
| United Arab Emirates | 0.03(-0.04,0.09) | 0.03(-0.04,0.10) |
| United Kingdom | 0.30(0.23,0.36) | 0.30(0.23,0.36) |
| United Republic of Tanzania | 0.04(0.04,0.05) | 0.11(0.10,0.11) |
| United States Virgin Islands | 0.03(0.02,0.05) | 0.03(0.02,0.05) |
| United States of America | 0.09(0.07,0.12) | 0.08(0.05,0.11) |
| Uruguay | 0.01(-0.00,0.02) | 0.00(-0.01,0.01) |
| Uzbekistan | 0.03(0.02,0.04) | 0.04(0.03,0.05) |
| Vanuatu | -0.02(-0.03,-0.01) | -0.01(-0.02,0.00) |
| Venezuela (Bolivarian Republic of) | 0.03(0.01,0.05) | 0.04(0.02,0.07) |
| Viet Nam | 0.16(0.14,0.17) | 0.17(0.16,0.19) |
| Yemen | 0.01(-0.00,0.02) | 0.02(0.00,0.04) |
| Zambia | 0.08(0.07,0.08) | 0.11(0.09,0.12) |
| Zimbabwe | -0.06(-0.07,-0.05) | -0.05(-0.06,-0.04) |

**Table S7**. Cross-country absolute (slope index of inequality) and relative (concentration index) inequality in autism spectrum disorder DALY rates among adolescents and young adults, 1990 and 2021

| **Metric** | **Year** | **Atopic dermatitis** |
| --- | --- | --- |
| **All included** | | |
| **Slope index (SII)** | 1990 | 20.30 (10.37, 30.24) |
|  | 2021 | 22.53 (12.53, 32.53) |
| **Concentration index (CII)** | 1990 | 0.04 (0.02, 0.05) |
|  | 2021 | 0.04 (0.02, 0.05) |

**Table S8**. Frontier analysis of autism spectrum disorder DALY rates among adolescents and young adults relative to socio-demographic index across 204 countries and territories, 2021

| **Location** | **SDI** | **Rate of Incidence (95% CI)** | **Frontier Incidence** | **Effective difference** |
| --- | --- | --- | --- | --- |
| Afghanistan | 0.337199998 | 138.54 (94.18, 198.63) | 113.81 | 24.73447252 |
| Albania | 0.706849791 | 186.30 (125.32, 261.19) | 113.76 | 72.54031325 |
| Algeria | 0.659500924 | 151.76 (101.98, 214.02) | 113.78 | 37.97507094 |
| American Samoa | 0.723727533 | 139.19 (94.90, 198.24) | 113.75 | 25.4365638 |
| Andorra | 0.869444113 | 177.34 (118.93, 249.17) | 113.76 | 63.57988098 |
| Angola | 0.453721949 | 172.06 (117.25, 240.90) | 113.76 | 58.30293507 |
| Antigua and Barbuda | 0.749886887 | 140.16 (94.51, 199.12) | 113.76 | 26.39782857 |
| Argentina | 0.723122973 | 206.23 (139.26, 290.00) | 113.76 | 92.47424809 |
| Armenia | 0.701833194 | 181.97 (124.49, 259.26) | 113.76 | 68.21480208 |
| Australia | 0.844252814 | 230.62 (158.82, 325.37) | 113.77 | 116.8469183 |
| Austria | 0.853837004 | 176.10 (118.20, 247.34) | 113.77 | 62.33362634 |
| Azerbaijan | 0.694851274 | 176.71 (119.58, 247.21) | 113.76 | 62.9496924 |
| Bahamas | 0.805020668 | 136.90 (92.85, 193.91) | 113.77 | 23.13200361 |
| Bahrain | 0.753043204 | 169.89 (114.21, 239.33) | 113.76 | 56.12761473 |
| Bangladesh | 0.492420885 | 115.01 (77.64, 163.76) | 113.78 | 1.232536509 |
| Barbados | 0.746748764 | 140.48 (95.05, 199.40) | 113.77 | 26.70612288 |
| Belarus | 0.784484711 | 187.17 (126.56, 262.60) | 113.77 | 73.40068795 |
| Belgium | 0.853654016 | 173.72 (116.69, 243.65) | 113.76 | 59.96422853 |
| Belize | 0.610229002 | 133.96 (90.53, 189.39) | 113.77 | 20.18728171 |
| Benin | 0.373486574 | 175.15 (116.71, 248.02) | 113.78 | 61.37149657 |
| Bermuda | 0.821365422 | 144.88 (96.43, 204.45) | 113.78 | 31.09627955 |
| Bhutan | 0.473062378 | 128.82 (87.66, 182.47) | 113.75 | 15.06608181 |
| Bolivia (Plurinational State of) | 0.599010799 | 130.94 (89.14, 184.50) | 113.77 | 17.17424711 |
| Bosnia and Herzegovina | 0.723077893 | 191.32 (131.27, 271.57) | 113.78 | 77.53820091 |
| Botswana | 0.642721629 | 178.06 (122.01, 249.65) | 113.77 | 64.29631829 |
| Brazil | 0.653043887 | 120.45 (81.44, 169.41) | 113.76 | 6.696347297 |
| Brunei Darussalam | 0.810234367 | 274.28 (186.43, 383.61) | 113.77 | 160.5123954 |
| Bulgaria | 0.768150939 | 184.98 (125.70, 262.50) | 113.77 | 71.20496984 |
| Burkina Faso | 0.285118402 | 171.43 (115.33, 241.44) | 113.96 | 57.47193058 |
| Burundi | 0.289374365 | 173.98 (117.75, 247.53) | 113.98 | 59.99720822 |
| Cabo Verde | 0.533534539 | 188.76 (127.73, 266.22) | 113.78 | 74.98374277 |
| Cambodia | 0.473621491 | 134.33 (89.92, 190.87) | 113.77 | 20.5680377 |
| Cameroon | 0.479691223 | 175.30 (119.95, 248.30) | 113.76 | 61.53327033 |
| Canada | 0.87317068 | 217.97 (149.47, 309.40) | 113.78 | 104.1971773 |
| Central African Republic | 0.30916769 | 165.94 (113.10, 236.84) | 113.95 | 51.9876589 |
| Chad | 0.240436019 | 170.48 (116.10, 242.03) | 115.00 | 55.48215409 |
| Chile | 0.771514716 | 213.65 (145.28, 301.82) | 113.77 | 99.88106369 |
| China | 0.72162976 | 129.69 (88.19, 183.74) | 113.78 | 15.90756711 |
| Colombia | 0.655442913 | 152.55 (102.79, 217.58) | 113.76 | 38.78775344 |
| Comoros | 0.475978688 | 180.14 (123.35, 256.98) | 113.76 | 66.37769573 |
| Congo | 0.583075236 | 174.93 (119.02, 247.03) | 113.76 | 61.17005977 |
| Cook Islands | 0.779109955 | 139.59 (94.81, 198.42) | 113.78 | 25.81797332 |
| Costa Rica | 0.700340477 | 155.27 (104.03, 221.59) | 113.77 | 41.50170243 |
| Croatia | 0.798341027 | 195.58 (133.02, 279.62) | 113.76 | 81.82023882 |
| Cuba | 0.668729864 | 142.74 (97.43, 199.15) | 113.76 | 28.98678217 |
| Cyprus | 0.835630545 | 174.32 (116.46, 246.58) | 113.78 | 60.54197594 |
| Czechia | 0.828450433 | 195.59 (134.22, 276.85) | 113.77 | 81.8236664 |
| C么te d'Ivoire | 0.425941883 | 177.80 (119.48, 251.56) | 113.75 | 64.05304301 |
| Democratic People's Republic of Korea | 0.569854634 | 141.93 (96.32, 201.28) | 113.76 | 28.17009798 |
| Democratic Republic of the Congo | 0.383179849 | 174.69 (118.82, 251.70) | 113.78 | 60.90991862 |
| Denmark | 0.896424204 | 186.14 (126.36, 261.10) | 113.77 | 72.37586239 |
| Djibouti | 0.487958371 | 184.07 (126.22, 262.52) | 113.76 | 70.30892539 |
| Dominica | 0.746967185 | 137.52 (94.26, 197.30) | 113.78 | 23.74213519 |
| Dominican Republic | 0.619388201 | 133.74 (90.60, 187.13) | 113.77 | 19.9672843 |
| Ecuador | 0.661017053 | 134.69 (90.74, 191.50) | 113.76 | 20.92789385 |
| Egypt | 0.606787094 | 149.28 (100.16, 213.51) | 113.76 | 35.51812017 |
| El Salvador | 0.563775188 | 146.53 (99.08, 207.94) | 113.76 | 32.76902629 |
| Equatorial Guinea | 0.657857456 | 185.65 (125.20, 265.99) | 113.76 | 71.89432146 |
| Eritrea | 0.403863943 | 177.71 (121.46, 250.14) | 113.77 | 63.93206459 |
| Estonia | 0.844917787 | 190.30 (128.99, 266.91) | 113.78 | 76.52042162 |
| Eswatini | 0.585459713 | 173.23 (117.67, 242.57) | 113.75 | 59.47141646 |
| Ethiopia | 0.358823295 | 175.14 (121.88, 244.78) | 113.77 | 61.36599069 |
| Fiji | 0.675051631 | 135.40 (91.94, 191.49) | 113.75 | 21.64940789 |
| Finland | 0.859831368 | 163.49 (110.50, 234.41) | 113.75 | 49.73703958 |
| France | 0.838364875 | 172.80 (116.90, 244.56) | 113.77 | 59.03088961 |
| Gabon | 0.634691393 | 175.80 (118.02, 249.42) | 113.77 | 62.03195329 |
| Gambia | 0.40971416 | 175.38 (121.04, 247.77) | 113.78 | 61.60197377 |
| Georgia | 0.732473604 | 181.01 (124.24, 257.27) | 113.77 | 67.23887008 |
| Germany | 0.902957091 | 177.77 (120.81, 249.29) | 113.77 | 63.99889473 |
| Ghana | 0.56493039 | 176.30 (118.54, 247.00) | 113.78 | 62.52011355 |
| Greece | 0.791854408 | 172.68 (116.69, 245.24) | 113.76 | 58.91596745 |
| Greenland | 0.826210336 | 197.45 (133.55, 278.48) | 113.77 | 83.68146757 |
| Grenada | 0.668993028 | 138.40 (92.73, 196.11) | 113.76 | 24.63255791 |
| Guam | 0.803982203 | 143.70 (97.83, 203.89) | 113.77 | 29.93901022 |
| Guatemala | 0.539972424 | 142.08 (96.10, 201.01) | 113.77 | 28.30711119 |
| Guinea | 0.336401293 | 169.82 (115.69, 239.69) | 113.77 | 56.04927612 |
| Guinea-Bissau | 0.353109621 | 170.31 (116.84, 239.16) | 113.78 | 56.53457268 |
| Guyana | 0.650812335 | 130.22 (87.84, 183.48) | 113.76 | 16.46077282 |
| Haiti | 0.448278285 | 123.78 (83.91, 175.42) | 113.76 | 10.01875535 |
| Honduras | 0.513037248 | 143.03 (95.87, 203.44) | 113.77 | 29.26342648 |
| Hungary | 0.790754768 | 191.89 (128.81, 267.83) | 113.76 | 78.12972149 |
| Iceland | 0.87636168 | 205.93 (140.92, 287.32) | 113.77 | 92.16827746 |
| India | 0.575401649 | 139.68 (95.37, 196.71) | 113.76 | 25.91985679 |
| Indonesia | 0.656868336 | 128.29 (86.73, 181.43) | 113.78 | 14.51071742 |
| Iran (Islamic Republic of) | 0.697207398 | 153.90 (104.85, 216.65) | 113.77 | 40.13480945 |
| Iraq | 0.662626231 | 138.85 (93.96, 198.75) | 113.76 | 25.08864685 |
| Ireland | 0.87375385 | 208.22 (140.99, 288.60) | 113.76 | 94.45695506 |
| Israel | 0.809011652 | 157.09 (106.63, 220.99) | 113.77 | 43.32506395 |
| Italy | 0.805773534 | 196.85 (134.87, 275.84) | 113.79 | 83.06536255 |
| Jamaica | 0.683263064 | 138.34 (94.32, 195.55) | 113.77 | 24.57416213 |
| Japan | 0.871241813 | 311.87 (215.62, 436.99) | 113.77 | 198.103008 |
| Jordan | 0.725307227 | 159.74 (106.80, 224.60) | 113.78 | 45.95933194 |
| Kazakhstan | 0.725144495 | 177.57 (119.99, 253.27) | 113.77 | 63.79974284 |
| Kenya | 0.523768077 | 178.13 (122.28, 249.79) | 113.75 | 64.3816052 |
| Kiribati | 0.527186583 | 128.53 (86.50, 182.50) | 113.77 | 14.76518068 |
| Kuwait | 0.846651055 | 159.46 (107.23, 224.08) | 113.77 | 45.69716892 |
| Kyrgyzstan | 0.603979328 | 176.16 (118.20, 249.05) | 113.76 | 62.39958336 |
| Lao People's Democratic Republic | 0.489136091 | 132.57 (88.24, 189.43) | 113.77 | 18.79939735 |
| Latvia | 0.830663516 | 186.79 (127.00, 263.89) | 113.76 | 73.02844537 |
| Lebanon | 0.744746351 | 171.28 (116.00, 241.08) | 113.77 | 57.51360396 |
| Lesotho | 0.510393066 | 171.56 (116.56, 240.84) | 113.77 | 57.78593963 |
| Liberia | 0.352442452 | 175.00 (118.88, 244.89) | 113.77 | 61.23297995 |
| Libya | 0.725771399 | 150.46 (100.70, 212.91) | 113.77 | 36.68516749 |
| Lithuania | 0.856484049 | 187.21 (126.70, 262.98) | 113.76 | 73.44920524 |
| Luxembourg | 0.884428955 | 176.82 (120.47, 249.81) | 113.78 | 63.04627153 |
| Madagascar | 0.400246943 | 175.10 (120.43, 247.15) | 113.79 | 61.31397626 |
| Malawi | 0.384553634 | 174.35 (118.54, 246.40) | 113.76 | 60.58717412 |
| Malaysia | 0.742523828 | 145.14 (97.29, 203.55) | 113.75 | 31.38577696 |
| Maldives | 0.650886627 | 155.40 (102.50, 220.42) | 113.77 | 41.62860564 |
| Mali | 0.268579941 | 172.48 (118.00, 244.95) | 114.16 | 58.32283741 |
| Malta | 0.801585034 | 176.69 (118.76, 249.60) | 113.76 | 62.92902285 |
| Marshall Islands | 0.574091128 | 134.22 (91.07, 191.58) | 113.76 | 20.45973826 |
| Mauritania | 0.4989451 | 179.45 (120.79, 253.23) | 113.77 | 65.67634397 |
| Mauritius | 0.718260446 | 142.84 (96.45, 203.21) | 113.77 | 29.07124669 |
| Mexico | 0.664575304 | 149.41 (101.19, 210.82) | 113.76 | 35.64767894 |
| Micronesia (Federated States of) | 0.587534967 | 135.60 (90.53, 192.51) | 113.76 | 21.83648948 |
| Monaco | 0.908262831 | 175.33 (117.08, 247.20) | 113.77 | 61.5580276 |
| Mongolia | 0.617621565 | 170.80 (115.55, 240.34) | 113.76 | 57.03710922 |
| Montenegro | 0.795800584 | 192.23 (129.65, 270.08) | 113.78 | 78.45270495 |
| Morocco | 0.562698301 | 147.82 (98.38, 208.19) | 113.77 | 34.05272946 |
| Mozambique | 0.326462614 | 169.75 (116.68, 241.94) | 113.91 | 55.84178397 |
| Myanmar | 0.53390084 | 132.67 (88.41, 188.96) | 113.77 | 18.90244021 |
| Namibia | 0.617564872 | 178.63 (120.47, 252.93) | 113.77 | 64.86029755 |
| Nauru | 0.625177834 | 133.32 (89.67, 189.25) | 113.77 | 19.55095953 |
| Nepal | 0.433174635 | 121.58 (82.79, 172.02) | 113.76 | 7.826083731 |
| Netherlands | 0.888464256 | 176.61 (121.11, 249.77) | 113.76 | 62.85013931 |
| New Zealand | 0.849442499 | 226.63 (155.97, 318.09) | 113.77 | 112.8655184 |
| Nicaragua | 0.523958472 | 149.49 (100.47, 210.34) | 113.77 | 35.71567233 |
| Niger | 0.168072774 | 173.98 (117.79, 249.33) | 166.98 | 7.005140685 |
| Nigeria | 0.503390833 | 172.62 (119.28, 243.14) | 113.77 | 58.85157054 |
| Niue | 0.72622205 | 138.18 (93.15, 195.47) | 113.77 | 24.40351995 |
| North Macedonia | 0.750629703 | 188.19 (128.82, 267.11) | 113.77 | 74.42833121 |
| Northern Mariana Islands | 0.771535213 | 144.02 (96.71, 202.24) | 113.77 | 30.2512378 |
| Norway | 0.91613281 | 158.75 (109.06, 223.50) | 113.76 | 44.9863614 |
| Oman | 0.773391602 | 166.76 (112.57, 234.47) | 113.78 | 52.97704077 |
| Pakistan | 0.504028689 | 124.01 (84.90, 173.88) | 113.76 | 10.24951172 |
| Palau | 0.754046931 | 146.59 (96.98, 208.81) | 113.76 | 32.83350587 |
| Palestine | 0.631011665 | 152.52 (102.87, 214.22) | 113.78 | 38.74421265 |
| Panama | 0.708864828 | 153.40 (103.85, 216.42) | 113.78 | 39.61831457 |
| Papua New Guinea | 0.417797443 | 131.06 (88.93, 186.63) | 113.75 | 17.30053509 |
| Paraguay | 0.635718099 | 123.53 (83.23, 172.32) | 113.76 | 9.768730974 |
| Peru | 0.662054037 | 136.25 (91.52, 192.33) | 113.78 | 22.47063353 |
| Philippines | 0.651219329 | 135.69 (92.12, 190.72) | 113.77 | 21.91803231 |
| Poland | 0.812042809 | 190.10 (131.16, 265.39) | 113.76 | 76.33773452 |
| Portugal | 0.744151851 | 169.22 (115.11, 238.81) | 113.78 | 55.4420277 |
| Puerto Rico | 0.825525847 | 143.53 (97.17, 202.46) | 113.78 | 29.75266306 |
| Qatar | 0.846860584 | 194.76 (131.66, 274.90) | 113.77 | 80.99403579 |
| Republic of Korea | 0.886675267 | 297.78 (203.07, 415.54) | 113.77 | 184.0131477 |
| Republic of Moldova | 0.732214875 | 182.34 (123.82, 258.25) | 113.76 | 68.57650551 |
| Romania | 0.768453864 | 185.19 (125.65, 259.57) | 113.77 | 71.42126135 |
| Russian Federation | 0.808536005 | 183.61 (126.38, 258.43) | 113.76 | 69.84935968 |
| Rwanda | 0.435588706 | 176.49 (119.95, 248.49) | 113.76 | 62.72975084 |
| Saint Kitts and Nevis | 0.754987055 | 137.13 (92.73, 196.37) | 113.76 | 23.36914463 |
| Saint Lucia | 0.672509735 | 138.60 (92.26, 198.02) | 113.77 | 24.82692041 |
| Saint Vincent and the Grenadines | 0.637195963 | 136.81 (92.58, 193.90) | 113.77 | 23.04332051 |
| Samoa | 0.593392769 | 137.25 (92.48, 193.86) | 113.78 | 23.47902519 |
| San Marino | 0.888005474 | 174.43 (118.02, 245.44) | 113.78 | 60.65505551 |
| Sao Tome and Principe | 0.505413747 | 182.53 (124.19, 260.71) | 113.76 | 68.76068032 |
| Saudi Arabia | 0.815143493 | 160.73 (107.79, 229.05) | 113.76 | 46.97789477 |
| Senegal | 0.408054193 | 177.23 (120.95, 249.82) | 113.76 | 63.46526349 |
| Serbia | 0.792416294 | 191.42 (128.93, 266.92) | 113.76 | 77.65877579 |
| Seychelles | 0.730150775 | 146.66 (98.46, 208.08) | 113.77 | 32.88461768 |
| Sierra Leone | 0.358665881 | 174.10 (118.17, 247.26) | 113.77 | 60.32307271 |
| Singapore | 0.856097766 | 290.95 (197.82, 407.24) | 113.76 | 177.1899278 |
| Slovakia | 0.81061053 | 190.31 (129.58, 268.14) | 113.76 | 76.5520966 |
| Slovenia | 0.842430731 | 198.94 (134.79, 281.81) | 113.77 | 85.16736445 |
| Solomon Islands | 0.429360316 | 132.22 (89.20, 186.88) | 113.76 | 18.46644334 |
| Somalia | 0.077688109 | 173.41 (117.68, 245.83) | 170.80 | 2.60774057 |
| South Africa | 0.679626598 | 179.20 (122.54, 250.77) | 113.76 | 65.43111972 |
| South Sudan | 0.278371125 | 172.12 (117.59, 242.97) | 114.00 | 58.12068441 |
| Spain | 0.769283698 | 200.98 (137.92, 278.66) | 113.75 | 87.2304674 |
| Sri Lanka | 0.701534935 | 146.88 (100.05, 207.95) | 113.76 | 33.12069506 |
| Sudan | 0.541949735 | 143.26 (96.33, 203.89) | 113.77 | 29.48850953 |
| Suriname | 0.633665739 | 132.12 (89.08, 187.26) | 113.78 | 18.33907835 |
| Sweden | 0.886880299 | 201.08 (137.47, 282.53) | 113.77 | 87.31545531 |
| Switzerland | 0.933059111 | 176.75 (122.12, 250.22) | 113.75 | 63.00191161 |
| Syrian Arab Republic | 0.623004075 | 141.51 (94.88, 200.24) | 113.77 | 27.74122702 |
| Taiwan (Province of China) | 0.874747053 | 178.84 (120.50, 253.97) | 113.77 | 65.0721585 |
| Tajikistan | 0.541511187 | 171.77 (117.37, 243.58) | 113.76 | 58.00814977 |
| Thailand | 0.682547933 | 141.24 (96.70, 198.96) | 113.78 | 27.45933574 |
| Timor-Leste | 0.444667619 | 132.37 (89.91, 188.90) | 113.79 | 18.58414538 |
| Togo | 0.408533695 | 175.38 (118.16, 245.61) | 113.78 | 61.6017718 |
| Tokelau | 0.686425621 | 140.86 (94.44, 201.96) | 113.77 | 27.09610981 |
| Tonga | 0.626349936 | 136.18 (92.55, 194.01) | 113.77 | 22.41495946 |
| Trinidad and Tobago | 0.768763254 | 137.78 (93.01, 195.85) | 113.77 | 24.00971439 |
| Tunisia | 0.682432216 | 152.32 (101.35, 215.38) | 113.77 | 38.5558889 |
| Turkey | 0.712692673 | 154.38 (104.94, 217.69) | 113.77 | 40.6068055 |
| Turkmenistan | 0.682160776 | 177.05 (121.43, 250.86) | 113.77 | 63.27843973 |
| Tuvalu | 0.576620529 | 136.90 (91.93, 194.25) | 113.75 | 23.14411805 |
| Uganda | 0.423261181 | 176.81 (120.76, 248.77) | 113.76 | 63.04154273 |
| Ukraine | 0.760773913 | 182.65 (123.01, 257.04) | 113.76 | 68.88812431 |
| United Arab Emirates | 0.849317734 | 163.73 (109.64, 231.46) | 113.76 | 49.9662533 |
| United Kingdom | 0.859000182 | 146.38 (99.36, 205.64) | 113.76 | 32.61255608 |
| United Republic of Tanzania | 0.446568273 | 175.59 (118.75, 247.24) | 113.76 | 61.82672583 |
| United States of America | 0.862448354 | 212.04 (146.73, 296.71) | 113.77 | 98.26985814 |
| United States Virgin Islands | 0.821830853 | 139.38 (94.49, 197.11) | 113.77 | 25.6064657 |
| Uruguay | 0.719283445 | 208.73 (140.71, 298.18) | 113.76 | 94.97155585 |
| Uzbekistan | 0.662621694 | 173.82 (116.04, 246.64) | 113.77 | 60.0510818 |
| Vanuatu | 0.473100706 | 131.87 (89.18, 188.68) | 113.77 | 18.10649013 |
| Venezuela (Bolivarian Republic of) | 0.596513059 | 147.61 (99.83, 208.98) | 113.77 | 33.83710448 |
| Viet Nam | 0.627933721 | 144.34 (98.97, 202.61) | 113.76 | 30.58589148 |
| Yemen | 0.450376375 | 141.93 (95.53, 202.92) | 113.77 | 28.16107204 |
| Zambia | 0.505948954 | 175.04 (119.61, 248.79) | 113.76 | 61.27462471 |
| Zimbabwe | 0.473819486 | 172.08 (116.96, 244.94) | 113.76 | 58.32380214 |

**Table S9.** Region‑ and sex‑specific ASD burden in AYA: prevalent cases, DALYs, and age‑standardised rates (per 100 000).

|  |  | Prevalence (95% UI) | |  | Prevalence (95% UI) | |  | EAPC of ASPR (95% CI) |  | DALY (95% UI) | |  | DALY (95% UI) | |  | EAPC of ASDR (95% CI) |
| --- | --- | --- | --- | --- | --- | --- | --- | --- | --- | --- | --- | --- | --- | --- | --- | --- |
| Location | Sex | Cases, 1990 | ASPR (per 100,000), 1990 |  | Cases, 2021 | ASPR (per 100,000), 2021 |  | 1990-2021 |  | Cases, 1990 | ASDR (per 100,000), 1990 |  | Cases, 2021 | ASDR (per 100,000), 2021 |  | 1990-2021 |
| Global | Male | 11970707.05 (10080976.45 to 14129590.8) | 1079.86 (909.54 to 1275.04) | | 16403053.80 (13838920.58 to 19196491.73) | 1086.79 (917.38 to 1271.80) | | 0.03(0.02,0.03) |  | 2266550.00 (1548642.32 to 3190069.86) | 204.34 (139.65 to 287.86) | | 3108150.79 (2115017.90 to 4400144.59) | 205.98 (140.38 to 290.83) | | 0.04(0.03,0.04) |
| Global | Female | 5544427.11 (4627825.75 to 6598055.48) | 511.65 (426.86 to 608.80) | | 7727919.93 (6455282.83 to 9180412.06) | 528.10 (441.21 to 627.57) | | 0.12(0.10,0.14) |  | 1034226.88 (709973.05 to 1458909.62) | 95.37 (65.57 to 134.74) | | 1439310.35 (985289.87 to 2033299.41) | 98.40 (67.33 to 138.76) | | 0.13(0.11,0.14) |
| Global | Both | 17515134.16 (14741746.93 to 20719335.39) | 799.04 (672.13 to 945.55) | | 24130973.74 (20300728.74 to 28329702.55) | 811.67 (683.34 to 952.87) | | 0.07(-0.61,0.75) |  | 3300776.87 (2242510.64 to 4654828.48) | 150.49 (102.43 to 212.28) | | 4547461.14 (3083617.95 to 6410493.83) | 153.00 (103.77 to 215.64) | | 0.07(0.06,0.07) |
| High SDI | Male | 2632114.51 (2228927.43 to 3081152.57) | 1491.47 (1263.15 to 1746.56) | | 2710273.36 (2285344.95 to 3183009.16) | 1488.05 (1254.55 to 1747.50) | | -0.02(-0.05, -0.00) |  | 497614.41 (344413.67 to 704531.72) | 282.14 (194.70 to 397.50) | | 511180.00 (353710.78 to 722253.51) | 280.96 (194.04 to 396.24) | | -0.03(-0.05, -0.00) |
| High SDI | Female | 1133353.68 (946313.96 to 1341436.44) | 666.92 (556.84 to 789.03) | | 1131092.07 (942060.34 to 1340207.66) | 665.14 (554.10 to 788.28) | | -0.03(-0.04, -0.01) |  | 211418.38 (144684.83 to 295789.08) | 124.51 (85.45 to 174.44) | | 209975.88 (142062.60 to 295581.49) | 123.64 (83.77 to 173.94) | | -0.04(-0.05, -0.02) |
| High SDI | Both | 3765468.19 (3176485.19 to 4417070.77) | 1087.20 (917.07 to 1275.93) | | 3841365.42 (3228669.62 to 4523230.07) | 1090.72 (916.84 to 1284.41) | | -0.02(-0.75,0.72) |  | 709032.80 (488102.73 to 996570.01) | 204.86 (140.98 to 287.73) | | 721155.88 (495831.83 to 1010022.00) | 205.00 (140.90 to 286.86) | | -0.01(-0.02,0.01) |
| High-middle SDI | Male | 2545079.83 (2141859.08 to 3007849.83) | 1106.39 (930.87 to 1308.58) | | 2611211.47 (2195009.41 to 3088438.90) | 1144.12 (962.02 to 1350.47) | | 0.12(0.11,0.12) |  | 483170.79 (332732.29 to 687530.27) | 210.03 (144.58 to 297.64) | | 496093.16 (340681.41 to 701729.60) | 217.71 (149.23 to 306.23) | | 0.13(0.13,0.13) |
| High-middle SDI | Female | 1075343.22 (895145.89 to 1278180.52) | 483.51 (402.59 to 574.52) | | 1020329.12 (849453.07 to 1214947.69) | 485.75 (404.73 to 578.75) | | 0.05(0.02,0.08) |  | 201678.63 (136935.00 to 284113.80) | 90.68 (61.76 to 127.84) | | 191171.22 (128720.38 to 268637.12) | 91.20 (61.91 to 128.27) | | 0.06(0.03,0.09) |
| High-middle SDI | Both | 3620423.05 (3036584.73 to 4290455.09) | 800.17 (671.15 to 948.73) | | 3631540.59 (3046097.52 to 4283748.51) | 828.72 (695.58 to 976.54) |  | 0.10(-0.68,0.89) |  | 684849.42 (466836.44 to 972202.37) | 151.35 (103.64 to 214.26) | | 687264.39 (468815.63 to 971264.29) | 157.11 (107.17 to 221.23) | | 0.14(0.13,0.15) |
| Middle SDI | Male | 3618681.48 (3035430.42 to 4271937.74) | 946.37 (793.92 to 1116.64) | | 4668771.87 (3936037.35 to 5481051.57) | 990.04 (834.58 to 1162.88) | | 0.15(0.13,0.16) |  | 687622.82 (468284.96 to 969528.09) | 179.67 (122.71 to 252.87) | | 886678.10 (601620.44 to 1255126.14) | 188.13 (127.87 to 265.87) | | 0.16(0.14,0.17) |
| Middle SDI | Female | 1628475.66 (1350687.90 to 1946734.14) | 438.33 (363.50 to 524.07) | | 2140922.59 (1781983.87 to 2558350.70) | 471.18 (392.34 to 562.88) | | 0.27(0.26,0.28) |  | 305042.84 (209911.98 to 429987.75) | 82.01 (56.37 to 115.82) | | 399827.36 (272370.51 to 566422.26) | 88.08 (60.03 to 124.70) | | 0.27(0.26,0.29) |
| Middle SDI | Both | 5247157.15 (4413255.64 to 6215754.85) | 696.13 (584.65 to 824.70) | | 6809694.45 (5717071.41 to 8027773.05) | 735.45 (617.53 to 866.96) | | 0.20(-0.50,0.90) |  | 992665.66 (676728.49 to 1399744.73) | 131.56 (89.75 to 185.44) | | 1286505.46 (870596.76 to 1812846.11) | 139.04 (94.10 to 196.68) | | 0.19(0.18,0.20) |
| Low-middle SDI | Male | 2184780.12 (1835299.25 to 2589468.18) | 954.94 (802.00 to 1132.15) | | 3953128.59 (3329526.85 to 4616008.02) | 979.03 (823.96 to 1144.41) | | 0.08(0.07,0.08) |  | 412221.74 (283764.91 to 581116.46) | 179.98 (124.21 to 252.89) | | 748836.65 (508186.74 to 1054208.02) | 185.38 (126.19 to 260.36) | | 0.10(0.10,0.10) |
| Low-middle SDI | Female | 1147615.68 (958231.66 to 1371014.91) | 507.76 (423.92 to 606.30) | | 2062975.82 (1729748.28 to 2454054.68) | 516.30 (432.33 to 614.88) | | 0.05(0.04,0.05) |  | 212819.21 (146565.04 to 301271.25) | 94.01 (64.50 to 132.97) | | 383469.22 (263780.83 to 539780.46) | 95.92 (66.05 to 135.47) | | 0.07(0.06,0.07) |
| Low-middle SDI | Both | 3332395.80 (2798201.61 to 3955523.21) | 732.82 (614.83 to 869.93) | | 6016104.41 (5057111.45 to 7047007.05) | 748.84 (629.59 to 877.53) | | 0.06(-0.52,0.65) |  | 625040.95 (431019.40 to 882897.74) | 137.28 (94.90 to 193.77) | | 1132305.86 (770154.50 to 1594819.60) | 140.88 (96.11 to 198.36) | | 0.09(0.08,0.10) |
| Low SDI | Male | 979279.33 (821896.20 to 1154648.05) | 1073.48 (900.90 to 1265.87) | | 2447334.68 (2052713.92 to 2873067.35) | 1099.67 (922.86 to 1291.68) | | 0.08(0.07,0.08) |  | 183882.90 (127476.09 to 258305.74) | 201.33 (140.01 to 282.16) | | 463025.08 (315753.97 to 652341.72) | 207.77 (142.60 to 291.95) | | 0.11(0.10,0.12) |
| Low SDI | Female | 554052.76 (464523.24 to 659059.00) | 589.75 (494.10 to 700.89) | | 1366481.26 (1145839.28 to 1616156.75) | 598.02 (501.06 to 708.60) | | 0.04(0.04,0.05) |  | 102222.51 (70883.39 to 144501.62) | 108.59 (74.82 to 153.09) | | 253724.15 (176827.90 to 361861.73) | 110.81 (76.86 to 155.81) | | 0.07(0.07,0.08) |
| Low SDI | Both | 1533332.09 (1290487.95 to 1810303.92) | 827.85 (695.97 to 978.44) | | 3813815.94 (3199662.18 to 4484005.20) | 845.15 (708.88 to 993.42) | | 0.06(-0.49,0.61) |  | 286105.41 (198781.26 to 404156.98) | 154.23 (107.11 to 217.23) | | 716749.23 (494308.14 to 1008941.11) | 158.57 (109.42 to 222.58) | | 0.10(0.09,0.10) |
| Andean Latin America | Male | 69541.15 (58208.75 to 82578.27) | 914.79 (766.00 to 1086.04) | | 128149.74 (106745.03 to 151499.04) | 938.26 (781.05 to 1110.12) | | 0.09(0.08,0.09) |  | 13180.49 (9118.78 to 18515.29) | 173.26 (117.78 to 244.72) | | 24324.38 (16541.04 to 34014.17) | 178.08 (120.83 to 251.55) | | 0.10(0.09,0.11) |
| Andean Latin America | Female | 38219.65 (31689.11 to 46010.76) | 483.60 (401.07 to 582.20) | | 65278.98 (54235.34 to 78194.89) | 486.38 (403.86 to 582.50) | | 0.01(0.01,0.02) |  | 7161.81 (4859.01 to 9903.06) | 90.49 (60.61 to 128.12) | | 12192.28 (8181.94 to 17195.37) | 90.87 (60.44 to 128.48) | | 0.02(0.02,0.03) |
| Andean Latin America | Both | 107760.80 (90428.17 to 128865.33) | 694.86 (582.66 to 830.33) | | 193428.73 (161784.27 to 229617.21) | 714.31 (597.61 to 847.97) | | 0.06(-0.53,0.66) |  | 20342.30 (13949.98 to 28364.73) | 131.04 (89.79 to 183.91) | | 36516.66 (24776.46 to 51114.35) | 134.86 (91.68 to 189.30) | | 0.10(0.09,0.10) |
| Australasia | Male | 68311.51 (56529.78 to 81054.64) | 1671.83 (1383.69 to 1984.37) | | 90122.91 (75046.15 to 108380.28) | 1713.11 (1426.79 to 2059.51) | | 0.09(0.08,0.09) |  | 12799.97 (8873.86 to 17896.65) | 313.33 (213.75 to 439.46) | | 16946.28 (11894.71 to 23879.88) | 322.40 (223.13 to 452.07) | | 0.10(0.09,0.11) |
| Australasia | Female | 29776.37 (24554.89 to 35863.30) | 732.44 (604.01 to 880.94) | | 38205.23 (31685.63 to 46468.83) | 734.40 (609.13 to 892.99) | | 0.01(0.01,0.02) |  | 5490.47 (3806.80 to 7721.59) | 135.11 (91.02 to 193.67) | | 7062.66 (4664.34 to 9939.67) | 135.93 (90.43 to 195.29) | | 0.02(0.01,0.03) |
| Australasia | Both | 98087.88 (81810.16 to 115828.15) | 1203.52 (1003.48 to 1421.00) | | 128328.14 (107238.00 to 153271.48) | 1227.71 (1025.41 to 1466.40) | | 0.06(-0.71,0.82) |  | 18290.45 (12656.36 to 25510.38) | 224.49 (154.46 to 313.33) | | 24008.94 (16554.07 to 33999.53) | 229.93 (158.85 to 323.18) | | 0.08(0.08,0.09) |
| Caribbean | Male | 69208.19 (58129.74 to 82034.40) | 949.28 (797.36 to 1125.05) | | 85018.30 (71766.91 to 100829.54) | 935.01 (788.39 to 1109.44) | | -0.05(-0.05, -0.04) |  | 13095.54 (8988.56 to 18354.52) | 179.45 (121.82 to 251.29) | | 16097.73 (11001.37 to 22403.15) | 177.05 (121.60 to 246.70) | | -0.04(-0.04, -0.03) |
| Caribbean | Female | 37793.62 (31328.32 to 45159.56) | 497.14 (411.77 to 594.08) | | 44134.44 (36460.28 to 52916.22) | 484.71 (400.34 to 580.99) | | -0.08(-0.09, -0.07) |  | 7050.33 (4859.06 to 9948.26) | 92.64 (63.60 to 131.09) | | 8201.21 (5768.42 to 11674.18) | 90.10 (61.96 to 127.41) | | -0.08(-0.09, -0.08) |
| Caribbean | Both | 107001.81 (89574.26 to 127324.03) | 718.22 (601.11 to 854.35) | | 129152.74 (108337.01 to 153643.30) | 709.73 (595.65 to 844.12) | | -0.05(-0.64,0.54) |  | 20145.88 (13702.06 to 28318.79) | 135.09 (91.92 to 189.85) | | 24298.94 (16549.69 to 33983.08) | 133.55 (91.85 to 186.70) | | -0.03(-0.03, -0.02) |
| Central Asia | Male | 169051.52 (142074.80 to 201083.04) | 1192.17 (1001.50 to 1417.96) | | 226133.48 (190224.10 to 268200.39) | 1196.56 (1005.84 to 1418.55) | | 0.03(0.02,0.04) |  | 32096.58 (21597.00 to 45391.01) | 226.21 (154.14 to 319.12) | | 42926.44 (29478.01 to 61283.34) | 227.29 (155.83 to 320.40) | | 0.04(0.03,0.05) |
| Central Asia | Female | 93959.86 (78311.40 to 112447.35) | 657.02 (547.59 to 786.14) | | 120061.97 (99780.61 to 141585.69) | 650.86 (541.25 to 767.90) | | -0.02(-0.02, -0.01) |  | 17621.57 (12013.45 to 24463.00) | 123.16 (84.23 to 172.79) | | 22458.75 (15356.20 to 32050.39) | 121.89 (83.23 to 172.45) | | -0.01(-0.01, -0.00) |
| Central Asia | Both | 263011.39 (220699.71 to 311702.22) | 923.33 (774.89 to 1094.60) | | 346195.45 (290543.68 to 408914.55) | 927.34 (777.96 to 1094.49) | | 0.01(-0.53,0.56) |  | 49718.15 (33474.08 to 69817.48) | 174.45 (119.08 to 245.59) | | 65385.19 (44817.53 to 93237.39) | 175.29 (119.85 to 248.64) | | 0.04(0.03,0.06) |
| Central Europe | Male | 300455.37 (253688.27 to 351982.84) | 1263.88 (1066.38 to 1481.19) | | 233547.50 (196203.15 to 275380.73) | 1301.72 (1093.39 to 1534.72) | | 0.11(0.10,0.12) |  | 56795.13 (39082.16 to 80100.60) | 239.09 (164.38 to 336.07) | | 44279.47 (30711.65 to 62307.55) | 247.20 (170.87 to 346.90) | | 0.13(0.12,0.13) |
| Central Europe | Female | 159176.64 (133142.09 to 188168.33) | 690.99 (577.73 to 817.08) | | 117363.10 (97751.13 to 139380.52) | 689.64 (574.76 to 818.13) | | -0.01(-0.02, -0.01) |  | 29871.67 (20515.11 to 41720.22) | 129.77 (89.31 to 181.73) | | 22027.58 (14980.43 to 30562.54) | 129.67 (88.37 to 180.37) | | -0.00(-0.01,0.00) |
| Central Europe | Both | 459632.01 (388490.86 to 541667.50) | 982.06 (830.14 to 1157.31) | | 350910.60 (294467.41 to 413692.06) | 1003.92 (842.39 to 1183.58) | | 0.06(-0.51,0.63) |  | 86666.81 (59253.81 to 122685.62) | 185.31 (127.18 to 260.76) | | 66307.05 (45780.06 to 92848.56) | 190.02 (130.54 to 265.76) | | 0.09(0.09,0.10) |
| Central Latin America | Male | 344804.93 (289597.88 to 409151.87) | 1037.35 (870.89 to 1231.41) | | 525408.65 (441567.94 to 618252.98) | 1057.90 (889.87 to 1245.33) | | 0.06(0.06,0.06) |  | 65178.76 (45229.55 to 91377.62) | 195.81 (135.82 to 274.04) | | 99581.46 (66753.80 to 141347.46) | 200.47 (135.30 to 283.06) | | 0.07(0.06,0.08) |
| Central Latin America | Female | 188933.17 (157194.04 to 225026.03) | 536.72 (446.68 to 639.42) | | 275260.00 (228647.89 to 328687.79) | 534.49 (444.28 to 638.40) | | -0.01(-0.02, -0.01) |  | 35406.27 (24238.34 to 50068.78) | 100.40 (68.59 to 141.93) | | 51391.86 (35191.57 to 72535.58) | 99.81 (68.84 to 142.15) | | -0.02(-0.02, -0.01) |
| Central Latin America | Both | 533738.09 (448251.75 to 634135.07) | 779.64 (654.01 to 926.75) | | 800668.65 (672230.73 to 948479.29) | 791.44 (665.12 to 936.53) | | 0.03(-0.58,0.65) |  | 100585.04 (69226.32 to 141402.48) | 146.70 (101.12 to 206.45) | | 150973.31 (101851.59 to 214063.65) | 149.23 (101.02 to 211.33) | | 0.06(0.05,0.06) |
| Central Sub-Saharan Africa | Male | 122721.37 (102945.11 to 146415.78) | 1184.09 (993.60 to 1412.21) | | 326588.72 (274140.93 to 387665.84) | 1205.68 (1011.55 to 1430.81) | | 0.05(0.04,0.06) |  | 22879.11 (15732.98 to 32247.62) | 220.45 (149.73 to 311.41) | | 61391.26 (42084.10 to 86807.03) | 226.32 (155.98 to 321.27) | | 0.09(0.08,0.11) |
| Central Sub-Saharan Africa | Female | 68239.28 (56600.65 to 81559.88) | 650.80 (540.29 to 777.60) | | 179403.31 (148854.82 to 213909.35) | 659.76 (547.65 to 787.02) | | 0.03(0.03,0.04) |  | 12495.32 (8628.10 to 17840.95) | 118.88 (78.90 to 170.07) | | 33183.28 (22717.91 to 47368.42) | 121.76 (81.03 to 174.93) | | 0.07(0.07,0.08) |
| Central Sub-Saharan Africa | Both | 190960.64 (161208.78 to 227735.77) | 915.59 (772.02 to 1090.79) | | 505992.03 (422558.68 to 599187.73) | 932.05 (779.67 to 1104.18) | | 0.05(-0.50,0.59) |  | 35374.43 (24389.86 to 50091.96) | 169.31 (115.88 to 238.88) | | 94574.54 (64773.38 to 133683.75) | 173.91 (119.31 to 248.12) | | 0.09(0.08,0.09) |
| East Asia | Male | 2709672.77 (2266803.20 to 3209826.90) | 931.02 (778.87 to 1102.14) | | 2502173.84 (2086961.16 to 2969259.53) | 999.11 (833.58 to 1185.60) | | 0.24(0.21,0.26) |  | 517709.05 (355930.60 to 733589.42) | 177.79 (122.08 to 251.51) | | 477814.40 (326579.77 to 674255.91) | 191.11 (131.27 to 270.54) | | 0.24(0.22,0.27) |
| East Asia | Female | 922810.98 (757430.89 to 1108159.13) | 335.56 (275.54 to 402.99) | | 766376.73 (631452.96 to 923873.55) | 336.39 (277.04 to 405.88) | | 0.14(0.09,0.18) |  | 174266.28 (118530.10 to 244504.88) | 63.32 (42.84 to 89.18) | | 145187.55 (96846.50 to 203904.01) | 63.86 (43.01 to 89.45) | | 0.16(0.11,0.20) |
| East Asia | Both | 3632483.76 (3036433.59 to 4297718.61) | 641.92 (536.24 to 759.37) | | 3268550.58 (2724979.06 to 3890853.98) | 684.64 (570.87 to 814.95) | | 0.20(-0.79,1.19) |  | 691975.33 (470325.20 to 977738.00) | 122.22 (83.20 to 173.53) | | 623001.96 (420495.36 to 878183.38) | 130.73 (88.95 to 185.10) | | 0.24(0.22,0.26) |
| Eastern Europe | Male | 532508.80 (449023.40 to 626210.19) | 1235.64 (1041.99 to 1451.83) | | 422662.51 (355657.54 to 501843.01) | 1265.67 (1064.70 to 1502.94) | | 0.09(0.08,0.11) |  | 100386.84 (68639.12 to 142371.24) | 233.25 (159.78 to 327.73) | | 79709.59 (54629.67 to 112244.72) | 239.39 (165.07 to 334.92) | | 0.12(0.10,0.13) |
| Eastern Europe | Female | 287820.20 (240220.90 to 342716.89) | 676.79 (564.78 to 805.51) | | 219643.32 (182497.76 to 262051.62) | 674.33 (560.67 to 804.39) | | -0.01(-0.01, -0.01) |  | 53840.22 (36805.79 to 75395.61) | 126.80 (86.74 to 177.52) | | 40965.11 (27944.67 to 57273.50) | 126.18 (86.44 to 176.08) | | -0.01(-0.01, -0.00) |
| Eastern Europe | Both | 820329.00 (690401.67 to 971100.44) | 958.41 (806.80 to 1134.21) | | 642305.83 (539335.32 to 763864.55) | 974.54 (818.15 to 1158.89) | | 0.05(-0.51,0.61) |  | 154227.07 (104677.55 to 218652.98) | 180.45 (123.41 to 255.73) | | 120674.70 (82508.06 to 169639.54) | 183.66 (126.07 to 258.36) | | 0.08(0.07,0.10) |
| Eastern Sub-Saharan Africa | Male | 409126.66 (343283.30 to 480851.38) | 1194.84 (1003.07 to 1405.97) | | 1045542.30 (883557.53 to 1224294.72) | 1218.98 (1029.22 to 1428.82) | | 0.06(0.06,0.07) |  | 76932.61 (53001.56 to 108405.53) | 224.36 (154.99 to 315.10) | | 197686.89 (135459.63 to 278005.14) | 230.17 (158.40 to 321.41) | | 0.10(0.09,0.10) |
| Eastern Sub-Saharan Africa | Female | 242794.24 (203524.76 to 287850.01) | 656.11 (549.37 to 777.86) | | 598068.97 (503745.29 to 704997.75) | 663.91 (557.97 to 784.90) | | 0.03(0.03,0.04) |  | 44938.46 (31099.39 to 63342.71) | 121.15 (83.33 to 170.99) | | 111269.60 (77610.54 to 158980.39) | 123.24 (85.46 to 173.55) | | 0.07(0.07,0.08) |
| Eastern Sub-Saharan Africa | Both | 651920.90 (547998.34 to 765768.41) | 914.77 (769.43 to 1076.97) | | 1643611.27 (1384143.42 to 1926037.38) | 934.06 (786.37 to 1095.37) | | 0.05(-0.49,0.60) |  | 121871.07 (84125.55 to 172306.77) | 170.70 (118.29 to 240.79) | | 308956.50 (212569.35 to 434777.39) | 175.28 (120.76 to 246.35) | | 0.10(0.09,0.10) |
| High-income Asia Pacific | Male | 713237.95 (602459.83 to 834083.82) | 2076.90 (1754.15 to 2428.89) | | 577079.11 (485525.75 to 676784.82) | 2217.83 (1866.59 to 2600.91) | | 0.22(0.19,0.24) |  | 135621.50 (93632.29 to 189527.98) | 394.91 (274.58 to 551.88) | | 109745.30 (76136.10 to 155475.89) | 422.16 (291.58 to 592.64) | | 0.22(0.20,0.25) |
| High-income Asia Pacific | Female | 301765.91 (253391.85 to 357220.28) | 910.30 (764.14 to 1077.11) | | 238664.26 (200788.55 to 281065.16) | 975.85 (820.85 to 1149.25) | | 0.21(0.19,0.22) |  | 56724.49 (39164.74 to 79617.17) | 171.12 (116.93 to 239.91) | | 44812.29 (30690.35 to 62274.29) | 183.46 (125.61 to 256.17) | | 0.21(0.19,0.23) |
| High-income Asia Pacific | Both | 1015003.85 (858023.29 to 1186945.45) | 1503.79 (1270.90 to 1759.18) | | 815743.38 (686285.79 to 957390.09) | 1616.19 (1360.84 to 1896.81) | | 0.22(-0.54,0.98) |  | 192345.99 (132883.65 to 269085.72) | 284.97 (197.01 to 399.17) | | 154557.59 (107016.19 to 218056.87) | 306.53 (210.96 to 430.00) | | 0.24(0.22,0.26) |
| High-income North America | Male | 857149.68 (725660.87 to 1007492.36) | 1504.52 (1273.62 to 1768.81) | | 955192.74 (801693.07 to 1120499.58) | 1536.85 (1289.11 to 1802.91) | | 0.10(0.08,0.13) |  | 161191.62 (112221.42 to 226776.49) | 283.18 (195.04 to 398.13) | | 178762.11 (123628.13 to 251443.42) | 287.79 (199.38 to 402.93) | | 0.09(0.06,0.12) |
| High-income North America | Female | 411358.69 (341894.49 to 488468.00) | 731.88 (608.32 to 868.77) | | 449621.77 (374915.14 to 532590.67) | 737.82 (615.25 to 873.64) | | 0.05(0.04,0.07) |  | 76353.46 (52585.57 to 106109.62) | 136.02 (92.84 to 188.83) | | 82672.92 (56491.72 to 115984.09) | 135.79 (93.39 to 189.24) | | 0.03(0.01,0.05) |
| High-income North America | Both | 1268508.37 (1069884.72 to 1493518.81) | 1121.39 (946.14 to 1320.38) | | 1404814.52 (1181455.22 to 1654240.49) | 1141.60 (960.14 to 1344.64) | | 0.08(-0.58,0.75) |  | 237545.08 (163612.71 to 333559.86) | 210.21 (144.41 to 294.40) | | 261435.04 (180887.77 to 367389.12) | 212.60 (147.00 to 298.12) | | 0.07(0.05,0.10) |
| North Africa and Middle East | Male | 703168.59 (589729.21 to 830440.33) | 1024.24 (858.37 to 1210.18) | | 1372586.11 (1159506.99 to 1616165.36) | 1037.51 (876.47 to 1222.34) | | 0.05(0.04,0.05) |  | 133008.68 (91732.69 to 187530.77) | 193.54 (134.08 to 271.33) | | 259597.68 (175922.22 to 368609.36) | 196.30 (133.51 to 277.78) | | 0.06(0.05,0.06) |
| North Africa and Middle East | Female | 359760.19 (299831.00 to 427957.67) | 549.45 (457.69 to 653.93) | | 667572.38 (554905.33 to 793434.72) | 547.72 (455.32 to 651.67) | | -0.01(-0.01, -0.00) |  | 66828.76 (46067.63 to 93795.82) | 101.84 (70.01 to 142.95) | | 123669.94 (83844.48 to 174239.03) | 101.52 (69.32 to 143.37) | | -0.00(-0.01,0.00) |
| North Africa and Middle East | Both | 1062928.78 (892289.67 to 1254882.78) | 792.67 (665.19 to 936.82) | | 2040158.49 (1716454.72 to 2409406.08) | 802.48 (674.69 to 947.62) | | 0.03(-0.54,0.61) |  | 199837.44 (137780.41 to 280994.26) | 148.82 (102.98 to 208.65) | | 383267.62 (258236.36 to 543988.44) | 150.82 (102.16 to 213.57) | | 0.06(0.05,0.07) |
| Oceania | Male | 12848.25 (10774.75 to 15237.63) | 941.77 (790.10 to 1117.72) | | 26898.60 (22554.22 to 32219.94) | 934.25 (782.23 to 1119.28) | | -0.03(-0.04, -0.03) |  | 2429.80 (1661.77 to 3442.67) | 177.94 (121.89 to 252.42) | | 5097.62 (3562.13 to 7172.75) | 176.95 (119.86 to 251.28) | | -0.02(-0.03, -0.02) |
| Oceania | Female | 5999.68 (4980.62 to 7185.79) | 462.01 (383.51 to 553.76) | | 12562.68 (10337.88 to 15127.25) | 455.00 (374.31 to 547.94) | | -0.05(-0.05, -0.05) |  | 1119.27 (761.88 to 1567.83) | 86.12 (56.80 to 122.30) | | 2344.32 (1580.93 to 3300.21) | 84.88 (55.85 to 121.72) | | -0.04(-0.05, -0.03) |
| Oceania | Both | 18847.93 (15795.27 to 22317.46) | 707.83 (592.88 to 838.64) | | 39461.29 (33208.12 to 47321.38) | 699.31 (587.82 to 838.97) | | -0.04(-0.69,0.61) |  | 3549.07 (2424.87 to 4986.36) | 133.16 (90.87 to 188.28) | | 7441.94 (5134.54 to 10420.11) | 131.82 (90.08 to 186.14) | | -0.03(-0.03, -0.03) |
| South Asia | Male | 2074302.29 (1744071.41 to 2452149.49) | 929.04 (780.79 to 1098.24) | | 3788573.75 (3187941.64 to 4415737.37) | 938.84 (790.42 to 1094.41) | | 0.03(0.03,0.04) |  | 391125.36 (269615.37 to 550041.72) | 175.01 (120.91 to 245.30) | | 717341.14 (487014.42 to 1010491.91) | 177.70 (121.36 to 249.21) | | 0.05(0.05,0.06) |
| South Asia | Female | 1030372.46 (860758.39 to 1229562.32) | 492.12 (411.00 to 587.21) | | 1905252.90 (1590448.36 to 2271424.39) | 491.36 (410.43 to 586.00) | | -0.01(-0.02, -0.01) |  | 190505.75 (130809.96 to 269467.58) | 90.86 (62.45 to 128.68) | | 353457.04 (245035.09 to 500373.75) | 91.13 (62.96 to 128.33) | | 0.01(0.01,0.01) |
| South Asia | Both | 3104674.75 (2609082.14 to 3683160.66) | 717.69 (602.50 to 850.99) | | 5693826.65 (4796024.79 to 6653290.45) | 719.54 (605.54 to 841.06) | | 0.01(-0.58,0.60) |  | 581631.12 (401396.99 to 818102.32) | 134.30 (92.83 to 188.78) | | 1070798.18 (730999.05 to 1501889.33) | 135.28 (92.41 to 190.38) | | 0.03(0.02,0.03) |
| Southeast Asia | Male | 886795.65 (745178.70 to 1045395.84) | 913.33 (767.03 to 1077.50) | | 1314735.53 (1108383.84 to 1551244.32) | 933.79 (786.47 to 1101.72) | | 0.07(0.06,0.09) |  | 168314.25 (115922.62 to 235767.97) | 173.21 (119.26 to 242.21) | | 250619.02 (172008.98 to 354052.45) | 178.04 (122.65 to 249.90) | | 0.10(0.08,0.11) |
| Southeast Asia | Female | 481132.82 (400168.61 to 575953.62) | 479.78 (399.06 to 574.22) | | 652937.99 (542495.92 to 783824.88) | 478.69 (398.07 to 574.61) | | 0.00(-0.00,0.01) |  | 90431.88 (62351.37 to 128126.91) | 90.10 (61.71 to 127.24) | | 122873.84 (83921.20 to 176031.34) | 90.12 (61.25 to 128.14) | | 0.02(0.01,0.03) |
| Southeast Asia | Both | 1367928.46 (1149830.73 to 1618769.77) | 693.10 (582.36 to 820.35) | | 1967673.51 (1654010.32 to 2329423.76) | 709.87 (596.32 to 840.39) | | 0.05(-0.55,0.66) |  | 258746.13 (177063.53 to 364997.05) | 130.99 (90.12 to 184.40) | | 373492.86 (254569.24 to 531084.93) | 134.78 (92.14 to 190.07) | | 0.11(0.09,0.12) |
| Southern Latin America | Male | 141112.39 (118336.79 to 166373.71) | 1493.77 (1250.75 to 1760.59) | | 195643.54 (163514.67 to 231181.90) | 1516.32 (1266.97 to 1792.23) | | 0.02(0.02,0.03) |  | 26675.86 (18631.95 to 37546.97) | 282.27 (194.24 to 397.19) | | 37052.89 (25194.63 to 52465.73) | 287.28 (193.39 to 405.13) | | 0.03(0.02,0.04) |
| Southern Latin America | Female | 66963.49 (55599.09 to 80583.79) | 694.39 (576.88 to 835.48) | | 89436.20 (73983.53 to 106630.81) | 694.43 (574.35 to 828.02) | | -0.00(-0.01, -0.00) |  | 12477.32 (8311.97 to 17468.49) | 129.33 (85.84 to 182.59) | | 16630.17 (11244.42 to 23305.69) | 129.23 (86.69 to 182.17) | | -0.00(-0.01,0.00) |
| Southern Latin America | Both | 208075.88 (174283.13 to 247384.14) | 1089.85 (912.50 to 1296.09) | | 285079.74 (239035.58 to 334904.66) | 1106.01 (926.99 to 1300.30) | | 0.01(-0.69,0.72) |  | 39153.17 (27067.46 to 55140.01) | 204.99 (140.24 to 287.75) | | 53683.06 (36598.74 to 75626.07) | 208.38 (142.11 to 293.13) | | 0.03(0.02,0.04) |
| Southern Sub-Saharan Africa | Male | 128123.58 (107684.28 to 151005.22) | 1222.72 (1026.98 to 1441.78) | | 209185.53 (175809.18 to 245540.37) | 1234.47 (1037.31 to 1450.27) | | 0.04(0.03,0.05) |  | 24196.75 (16717.76 to 33967.56) | 230.54 (158.45 to 323.60) | | 39303.82 (26837.80 to 55507.94) | 232.02 (158.23 to 327.51) | | 0.04(0.02,0.05) |
| Southern Sub-Saharan Africa | Female | 74760.95 (62495.08 to 88905.24) | 666.98 (556.70 to 792.58) | | 114527.57 (95828.49 to 136898.97) | 670.79 (561.14 to 801.86) | | 0.02(0.01,0.02) |  | 13887.20 (9624.83 to 19429.61) | 123.66 (84.62 to 172.70) | | 21071.93 (14560.32 to 29510.02) | 123.50 (85.50 to 173.69) | | 0.00(-0.01,0.01) |
| Southern Sub-Saharan Africa | Both | 202884.53 (170330.69 to 239470.83) | 935.65 (786.40 to 1103.80) | | 323713.10 (271766.75 to 384442.18) | 951.59 (800.08 to 1128.47) | | 0.04(-0.51,0.59) |  | 38083.95 (26455.61 to 53571.87) | 175.33 (120.78 to 246.20) | | 60375.76 (41218.02 to 85322.95) | 177.56 (121.98 to 250.15) | | 0.07(0.05,0.09) |
| Tropical Latin America | Male | 267494.33 (222749.31 to 317305.59) | 844.21 (703.00 to 1001.28) | | 375499.47 (314389.52 to 445895.20) | 856.00 (717.41 to 1016.03) | | 0.04(0.04,0.05) |  | 50419.51 (34778.15 to 70473.37) | 158.95 (109.71 to 222.39) | | 70717.06 (47598.59 to 99735.13) | 161.31 (109.40 to 227.21) | | 0.06(0.05,0.06) |
| Tropical Latin America | Female | 144776.74 (120256.30 to 172213.18) | 442.22 (367.28 to 526.10) | | 192459.78 (159041.72 to 231784.17) | 434.16 (358.73 to 522.80) | | -0.05(-0.06, -0.04) |  | 26802.45 (18695.92 to 38322.73) | 81.80 (55.85 to 117.30) | | 35475.37 (23877.59 to 49923.78) | 80.12 (54.18 to 112.87) | | -0.04(-0.06, -0.02) |
| Tropical Latin America | Both | 412271.07 (343243.75 to 489770.66) | 639.82 (532.74 to 760.40) | | 567959.25 (475255.43 to 675651.01) | 644.25 (538.79 to 766.62) | | 0.01(-0.60,0.61) |  | 77221.96 (53630.02 to 108924.38) | 119.73 (82.95 to 168.97) | | 106192.43 (71399.37 to 148782.67) | 120.56 (81.47 to 169.48) | | 0.04(0.03,0.04) |
| Western Europe | Male | 978171.44 (825944.90 to 1149693.92) | 1339.77 (1132.09 to 1574.65) | | 893550.39 (751030.56 to 1046443.23) | 1354.97 (1139.10 to 1586.80) | | 0.05(0.03,0.06) |  | 184786.73 (126782.13 to 262061.30) | 253.20 (172.79 to 356.80) | | 168744.32 (116594.41 to 234045.24) | 256.06 (177.21 to 356.23) | | 0.05(0.04,0.07) |
| Western Europe | Female | 354195.99 (293753.91 to 421150.14) | 498.68 (413.79 to 593.06) | | 317423.83 (264728.65 to 379959.87) | 498.37 (415.62 to 596.38) | | 0.01(0.00,0.02) |  | 65876.35 (45053.53 to 91535.50) | 92.80 (63.28 to 130.58) | | 58892.76 (40013.05 to 83024.00) | 92.56 (62.35 to 130.46) | | 0.00(-0.00,0.01) |
| Western Europe | Both | 1332367.44 (1122177.50 to 1568407.39) | 925.20 (779.21 to 1089.42) | | 1210974.23 (1018903.76 to 1421418.11) | 934.76 (786.41 to 1097.25) | | 0.03(-0.88,0.95) |  | 250663.08 (171196.06 to 354414.94) | 174.14 (118.82 to 245.44) | | 227637.08 (155549.81 to 317352.56) | 175.86 (120.72 to 245.63) | | 0.04(0.03,0.05) |
| Western Sub-Saharan Africa | Male | 412900.64 (346875.00 to 488255.28) | 1194.31 (1003.07 to 1412.55) | | 1108761.06 (933076.03 to 1304855.85) | 1214.88 (1022.43 to 1430.43) | | 0.06(0.06,0.07) |  | 77725.83 (53283.30 to 109332.17) | 224.63 (154.75 to 315.48) | | 210411.95 (143584.32 to 295868.45) | 230.28 (157.76 to 323.18) | | 0.10(0.09,0.10) |
| Western Sub-Saharan Africa | Female | 243816.17 (203431.23 to 288831.51) | 653.96 (545.34 to 775.19) | | 663664.51 (556540.58 to 784740.84) | 659.68 (552.47 to 780.15) | | 0.03(0.03,0.04) |  | 45077.53 (31116.45 to 63552.39) | 120.65 (83.52 to 170.25) | | 123469.85 (86077.79 to 174867.54) | 122.46 (84.99 to 172.57) | | 0.06(0.06,0.07) |
| Western Sub-Saharan Africa | Both | 656716.81 (551057.77 to 772413.29) | 915.26 (767.72 to 1078.01) | | 1772425.56 (1491590.81 to 2087365.27) | 923.31 (776.25 to 1087.66) | | 0.04(-0.50,0.60) |  | 122803.36 (84575.30 to 173699.50) | 170.93 (117.85 to 241.48) | | 333881.80 (230059.73 to 468286.39) | 173.66 (120.11 to 243.58) | | 0.07(0.06,0.08) |

**ASDR, age-standardised DALY rates; ASPR, age-standardised prevalence rates; CI, confidence interval; DALY, disability-adjusted life years; EAPC, estimated annual percentage change;** **AYA, adolescents and young adults; SDI, sociodemographic index; UI, uncertainty intervals.**
